# Temporal entry of pesticides through pollen into the bee hive and their fate in beeswax

**DOI:** 10.1007/s11356-024-35224-3

**Published:** 2024-10-15

**Authors:** Christina Kast, Jan Müller, Marion Fracheboud

**Affiliations:** Swiss Bee Research Centre, Agroscope, Schwarzenburgstrasse 161, 3003 Bern, Switzerland

**Keywords:** *Apis mellifera*, Honey bees, Pesticides, Pollen, Bee bread, Beeswax

## Abstract

Honey bees are often exposed to a variety of contaminants, including pesticides from agricultural use. The aim of this study was to investigate the temporal entry of pesticides into the hive by examining the seasonal timing of honey bees bringing pesticide-contaminated pollen into their colonies and the subsequent accumulation of these pesticides in beeswax. Pollen and beeswax samples were collected biweekly from five colonies situated in an agricultural environment in Switzerland. In pollen, 23 pesticides (out of 50) were quantified, including 4 insecticides, 4 herbicides, 12 fungicides, a transformation product, an acaricide, and a synergist. The maximal insecticide concentration levels measured in individual pollen samples were 69.4 μg/kg (thiacloprid), 48.3 μg/kg (acetamiprid), 13.8 μg/kg (spinosad), and 11.1 μg/kg (indoxacarb), while fungicide levels ranged up to 2212.7 μg/kg (cyprodinil), and herbicides were up to 71.9 μg/kg (prosulfocarb). Eighteen of the pesticides found in pollen were also quantifiable in beeswax. Among these were 17 lipophilic pesticides with logarithmic octanol water coefficients (log *K*_ow_) equal or above 2.5, which showed similar temporal profiles and order of accumulation magnitude as in pollen. For example, maximal concentrations measured in individual beeswax samples were 12.4 μg/kg for indoxacarb (insecticide), 986.4 μg/kg for cyprodinil (fungicide), and 21.6 μg/kg for prosulfocarb (herbicide). Furthermore, pesticides with log *K*_ow_ between 2.5 and 7.0 remained in the beeswax during wax purification. Our study shows that a large variety of pesticides brought into the hive through pollen potentially stay in the beeswax during recycling, thus constantly exposing honey bees to pesticides.

**Supplementary Information:**

The online version contains supplementary material available at 10.1007/s11356-024-35224-3.

## Introduction

As pollinators, honey bees play an important role in plant biodiversity as well as in agriculture. While foraging, they are often exposed to environmental pollutants, including pesticides used as plant protection products in agriculture (e.g., Porrini et al. 2016; Schaad et al. 2023), heavy metals from traffic or industry (Bogdanov 2006), and other contaminants, such as microplastics (Edo et al. 2021). Other important contaminants include pesticides authorized as veterinary drugs for use in apiculture to treat *Varroa destructor* (Bogdanov 2006). Honey bees’ foraging range depends on the availability of plants as food sources and their abundance around the hives, but they preferably forage within a distance of two kilometers from their nests and occasionally forage at longer distances up to 6 km (Visscher and Seeley 1982).

When bees collect pollen, nectar, water, or propolis, they bring pollutants into their hive. Nectar contains mainly hydrophilic substances, while pollen can contain a large variety of lipophilic and hydrophilic pesticides (Sanchez-Bayo and Goka 2014; Ostiguy et al. 2019). Consequently, these contaminants accumulate in various compartments of the hive (Végh et al. 2021; 2023). The distribution within the various hive compartments depends on the chemical properties of the contaminants. Lipophilic substances with a high logarithmic octanol water coefficient (log *K*_ow_) mainly accumulate in beeswax (Albero et al. 2023; Bogdanov 2004; Lozano et al. 2019; Murcia Morales et al. 2020), while relatively hydrophilic compounds, such as neonicotinoids, are frequently found in honey (Johnson et al. 2010; Sanchez-Bayo and Goka 2014). A variety of pesticides used as plant protection products in agriculture is lipophilic and tends to accumulate in beeswax (e.g., Calatayud-Vernich et al. 2017; El Agrebi et al. 2020; Marti et al. 2022; Mullin et al. 2010).

It is a common beekeeping practice to recycle beeswax from old combs for the production of new foundation sheets from which bees construct the final combs. A previous laboratory study in which old combs contaminated with acaricides from beekeeping were melted to produce new beeswax showed that bromopropylate, *tau*-fluvalinate, and coumaphos withstood high temperatures. These acaricides remained in the recycled wax at levels comparable to those of the comb wax before melting (Bogdanov et al. 1998). Thus, when old brood combs are recycled, a large number of pesticides from agricultural use may show a similar behavior as these acaricides and may remain in newly manufactured wax foundation sheets. Thus, it is not surprising that many studies have reported high pesticide contamination levels not only in brood combs but also in foundation beeswax (Calatayud-Vernich et al. 2017; Végh et al. 2023).

Studying the entrance and behavior of pesticides in beeswax is of interest, since honey bee larvae as well as adult bees are constantly exposed to these pesticides in wax*.* Adult bees come in contact with contaminated beeswax during their activity in the bee hive, for example, when building the cells. The developing bees are exposed from egg to emergence through contact with contaminants in the beeswax. As previously illustrated with coumaphos, pesticides can also migrate from wax into the larval jelly, thus exposing larvae orally, in addition to exposure through contact (Kast and Kilchenmann 2022).

Many studies analyzed pesticide residues in beeswax, but little is known about the seasonal changes of pesticide levels. Although the behavior of a few veterinary drugs used in apiculture has been previously studied in beeswax, there is little information on the long-term behavior of various pesticides from agriculture. However, this information is important for estimating the exposure risks for bees through contaminants in beeswax, since some of these pesticides, especially insecticides, might be highly toxic to bees even at low concentrations. In the current study, we show a pathway that leads to the accumulation of agrochemicals in beeswax and their long-term fate in wax. We examined a variety of pesticides, such as fungicides, herbicides, and insecticides, that are brought into the hive when bees collect pollen in an agricultural environment in Switzerland. In a realistic field scenario, we studied their occurrence in pollen, their storage in bee bread (pollen stored in cells), and finally their temporal distribution in beeswax.

Recently, we developed several methods using ultra-high performance liquid chromatographic analysis to quantitate pesticides in beeswax (21 pesticides; Marti et al. 2022) and bee bread (51 pesticides; Schaad et al. 2023). In the current study, we first validated the procedures for the quantitation of pesticides in pollen (50 pesticides) and beeswax (51 pesticides in total). In the second step, 48 pollen and 60 beeswax samples collected biweekly throughout the agricultural season were analyzed to study the time of entry of the pesticides into the hive through pollen and the subsequent temporal profile of their accumulation in beeswax. We also included previously published results for pesticides in matrix bee bread (Schaad et al. 2023) to complement the study. Third, we determined the proportion of pesticides that remain in beeswax when spiked beeswax is purified by melting wax in water. Together, this information helps us understand the time points during which pesticides from agriculture are incorporated into beeswax and explain their fate during the subsequent months. Finally, our study may also allow for predicting levels and the duration of the exposure of bees to pesticides in beeswax.

## Materials and methods

### Materials

The pesticides used as reference standards were purchased from LGC Standards GmbH (Wesel, Germany) or Merck (Darmstadt, Germany), as previously described (Schaad et al. 2023). More details can be found in the supplementary material (Section S1.1 Reference standards, solvents, and chemicals). The deuterated internal standard Clothianidin-D3 (C11691710) was purchased from LGC Standards GmbH (Wesel, Germany), while cyproconazole-D3 (91796), fluopyram-D4 (06899), terbuthylazine-D5 (91799), and thiacloprid-D4 (30673) were obtained from Merck (Darmstadt, Germany). The solvents and chemicals used for extraction and chromatography (Schaad et al. 2023) are also listed in the supplementary material (Section S1.1)*.*

### Honey bee colonies

The honey bee (*Apis mellifera*) colonies were located in an agricultural region (46°58′57.6″ N, 7°08′40.2″ E) in northwestern Switzerland with cultivations of oil seed rape, maize, sunflowers, and various vegetables (Schaad et al. 2023). The colonies were overwintered in 12-frame Dadant-Blatt hives on 7 to 8 frames. The combs were up to 3 years old. All colonies were treated against *Varroa destructor* infestation using formic and oxalic acids the years preceding the study (e.g. August and December 2021). In 2022, treatment against *V. destructor* infestation was performed with a Nassenheider Pro (290 mL formic acid 60%, wick 2). Four colonies were treated from August 19 to August 31, 2022, while treatment was not necessary for one of the colonies. The colonies were fed 5–7 L of syrup (60% sugar) from July 22 to September 6, 2022.

### Sampling of pollen, bee bread, and wax

Pollen and comb pieces consisting of bee bread and wax were sampled from five colonies throughout the crop season. Using a pollen trap (produced at a craft workshop) installed at the entrance of the hives, pollen was collected for a single day every second week from April 29 until August 18, 2022. After the formic acid treatment, a final sampling took place on October 4, 2022. On August 18 and October 4, 2022, pollen was obtained only from four colonies due to the lack of pollen in one of the colonies. In total, 48 pollen samples were collected on a total of 10 sampling dates during the crop season of 2022. Pollen was dried in a lyophilizator (Christ alpha 1–4, Kühner AG, Birsfelden, Switzerland) for 15 h at − 50 °C and 0.5 mbar. All samples were stored at − 20 °C until further use. The bee bread and wax samples were taken on 12 sampling dates as previously described (Schaad et al. 2023). The biweekly sampling started on March 29, 2022, and lasted until August 18, 2022. An additional and final sampling took place in the fall on October 4, 2022. A rectangle of approximately 30 cm^2^ containing fresh bee bread and wax was cut from two separate combs per colony (Schaad et al. 2023). Next, the bee bread was separated from the combs using a tool designed by Gürle Aricilik (Nilüfer Bursa, Turkey; www.gurlearicilik.com.tr). The bee bread from the two comb pieces of the same colony collected at each sampling date was combined and subsequently homogenized in a petri dish using a custom 3D-printed pestle (Schaad et al. 2023). In total, 60 bee bread samples were obtained. To obtain the wax samples, the two comb pieces from which all bee bread was removed as much as possible were wrapped in small silk organza cloth bags to extract the beeswax (Kast et al. 2020). The bags were placed for 1 h in a beaker containing 50 mL of distilled water at a temperature of 80 °C. Next, the wax was squeezed out from the bags, and the water was allowed to cool. Subsequently, hardened wax was collected from the surface of the water. The wax was melted once more at 80 °C for 10 min (without water) and shaken by hand for homogenization. In total, 60 wax samples were obtained.

### Pollen, bee bread, and wax for blank extracts

The pollen used as a blank extract or for spiking the pesticides to obtain recovery values was chosen for its overall low contamination level of pesticides. The pollen was produced in 2014 and was obtained from Bienen Roth GmbH (Wila, Switzerland). Nevertheless, the pollen contained low levels of the following pesticides: azoxystrobin (approx. 0.5 μg/kg), chlorpyrifos (approx. 4 μg/kg), cyproconazole (approx. 1 μg/kg), desthio-prothioconazole (approx. 2 μg/kg), difenoconazole (approx. 1 μg/kg), fluopyram (approx. 2 μg/kg), terbuthylazine (approx. 0.5 μg/kg), and thiacloprid (approx. 3 μg/kg). The bee bread used as a blank extract was collected in 2015 and 2017 from several honey bee colonies owned by Agroscope, located in Liebefeld, Switzerland. Although the colonies were located in an urban environment, the bee bread contained low residue levels of the following pesticides: azoxystrobin (approx. 3 μg/kg), trifloxystrobin (approx. 2 μg/kg), and difenoconazole (approx. 10 μg/kg). The beeswax used as a blank extract was from newly constructed combs produced in 2012. It contained low levels of the following pesticides: azoxystrobin (approx. 4 μg/kg), cyproconazole (approx. 4 μg/kg), difenoconazole (approx. 4 μg/kg), and trifloxystrobin (approx. 1 μg/kg). Due to these residue levels, the limits of quantifications (LOQs) were set accordingly, while the limits of detections (LODs) were not determined for the above-mentioned pesticides in the corresponding matrices. More details on how LOD and LOQ values were set are given in “Analysis by liquid chromatography and mass spectrometry (UHPLC-MS/MS)”.

### Extraction of pesticides

The extraction of pesticides followed a modified QUECHERS (quick, easy, cheap, efficient, rugged, safe) method in principle, as previously described by Schaad et al. (2023) and Marti et al. (2022). Two pollen or wax samples, respectively, with all pesticides at spiking levels of 20 µg/kg and 1000 µg/kg were included in each extraction series to control the extraction efficiency. Some minor modifications from the previously published extraction procedures concerned the use of internal standards. Previously, the pesticides in bee bread and wax were extracted with acetonitrile containing 50 µg/L clothianidin-D3 (Schaad et al. 2023) or caffeine 50 µg/L (Marti et al. 2022), respectively. In this study, other internal standards were included. The acetonitrile used for the extraction of pesticides from 1 g pollen and 0.5 g wax, respectively, contained 5 µg/L azoxystrobin-D4, 10 µg/L clothianidin-D3, 10 µg/L cyproconazole-D3, 5 µg/L fluopyram-D4, 5 µg/L terbuthylazine-D5, and 5 µg/L thiacloprid-D4. The detailed extraction procedure is described in the supplementary material (Section S1.2 Extraction of pesticides from pollen and wax).

### Analysis by liquid chromatography and mass spectrometry (UHPLC-MS/MS)

Liquid chromatography (LC) was performed with an Agilent 1290 Infinity II equipped with an autosampler and coupled with an Agilent 6495C tandem quadrupole mass spectrometer (MS) (Marti et al. 2022; Schaad et al. 2023). The injection volume was 1 µL. Chromatographic separation was performed on a C18 reverse phase column (Acquity UPLC HSS T3 Column, 100 Å, 1.8 µm, 2.1 mm × 100 mm) from Waters (Milford, Massachusetts, USA) at a temperature of 40 °C. The mobile phase A was 95% water + 5% acetonitrile + 0.01% formic acid + 5 mM ammonium formate, and the mobile phase B was 5% water + 95% acetonitrile + 0.01% formic acid + 5 mM ammonium formate. Three methods (M1, M2, and M3) with variable eluent gradients (Table S1) and ion source conditions of MS (Table S2) were used, as previously described for the analysis of the pesticides in bee bread (Schaad et al. 2023). The selected ion transitions used for the quantitation and identification of the pesticides in pollen, bee bread, and wax are listed in the supplementary material Table S3. One transition was used for quantitation and two additional transitions for identification (supplementary material Sects. 1.4 and 1.5). For some pesticides, the selected ion transitions used for quantitation (quantifiers) and identification (qualifiers) differed between the three matrices due to the various background contaminations of the matrices (supplementary material Table S3).

External matrix-matched calibration with nine concentration levels, ranging from 0.1 to 1000 µg/L, was used for quantitation of the pesticides. The concentrations of pesticides for which we did not include a deuterated internal standard were calculated based on the linear regression (1/x) of the calibration samples. The Agilent MassHunter quantitative software Version B.08.00 (Basel, Switzerland) was used for the calculations. Deuterated substances were used as internal standards for the quantitation of azoxystrobin, clothianidin, cyproconazole, fluopyram, terbuthylazine, and thiacloprid in pollen and wax (only clothianidin in bee bread; Schaad et al. 2023). The ratios of the areas of the concentration levels to the areas of the internal standards were used for creating a matrix-matched calibration curve as well as for the quantitation of the pesticides in the samples.

The LOD levels for each pesticide were experimentally determined by diluting spiked blank extracts (signal-to-noise ratio (s/n) at least 3:1). Recoveries were determined at pesticide spiking levels ranging from 0.5 to 1000 µg/kg (pollen) or 1 to 1000 µg/kg (wax) with at least five repetitions per spiking level. The lowest spiking level of an individual pesticide that showed a recovery of at least 75% and good linearity was set as its LOQ (except for acrinathrin [69%] and spirodiclofen [68%] in wax). The resulting LOD and LOQ values, as well as the recoveries at the quantification limits of each pesticide, are listed in Table 1. Further details regarding multiple spiking levels can be found in the supplementary material Section S1.3 Recoveries of pesticides in pollen at various spiking levels. The LOQs for pesticides that were present in the blank extracts were set to levels with acceptable recoveries, while the LODs for these compounds were not determined (Table 1).

**Table 1:** Detection and quantification limits for the pesticides as well as recoveries at the quantification limits

Pesticide	Class^1^	log *K*_ow_^2^	LOD^3^ [µg/kg]			LOQ^4^ [µg/kg]			Rec^5^ [%]		
			Pollen	Beebread	Wax	Pollen	Beebread	Wax	Pollen	Beebread	Wax
Acetamiprid	i	0.8^a^	0.4	0.4	1	0.5	1	2	108	99	106
Aclonifen	h	4.4^a^	4	5	50	10	5	100	110	124	84
Acrinathrin	a, i	6.3^a^	2	8	50	20	10	100	91	105	69
Azoxystrobin	f	2.5^a^	n.a.^6^	n.a.^6^	n.a.^6^	1	10	5	106	124	85
Bendiocarb	i	1.7^a^	0.4	0.4	1	0.5	1	2	120	106	108
Boscalid	f	3.0^a^	2	8	5	5	10	20	108	110	97
Bromopropylate	a, bk	5.4^a^	20	40	50	100	100	100	121	98	92
Chlorfenvinphos	i, a	3.8^a^	0.8	0.4	0.5	1	2	2	104	117	105
Chlorpyrifos	i	4.7^a^	n.a.^6^	2	2	20	10	5	101	95	90
Clothianidin	i	0.9^a^	0.8	2	1	2	2	2	110	103	125
Coumaphos	a, i, bk	4.1^b^	1	2	1	2	5	2	98	102	107
Lambda-cyhalothrin	i	5.5^a^	20	20	50	20	20	100	103	112	90
Zeta-cypermethrin	i	6.6^a^	20	20	100	20	20	100	98	106	90
Cyproconazole	f	3.1^a^	n.a.^6^	0.4	n.a.^6^	1	2.5	5	126	95	116
Cyprodinil	f	4.0^a^	0.8	0.4	0.5	2	2	5	100	99	90
Deltamethrin	i	4.6^a^	20	40	50	40	100	100	86	87	92
Difenoconazole	f	4.4^a^	n.a.^6^	n.a.^6^	n.a.^6^	2	10	5	101	72	127
Dimethoate	a, i	0.8^a^	0.4	0.5	1	1	1	2	111	117	108
Dimoxystrobin	f	3.6^a^	0.4	0.4	0.5	0.5	1	2	115	101	106
DMF (Amitraz)	TP, bk	1.5^b^	2	1	1	2	2	1	108	103	126
Fenhexamid	f	3.5^a^	5	4	50	20	5	100	89	93	88
Fenitrothion	i	3.3^a^	4	4	10	5	5	20	94	118	110
(E)-Fenpyroximate	a, i	5.7^a^	0.4	0.8	0.5	1	2	2	90	110	94
Fipronil	i	4.0^b^	0.2	0.2	0.2	0.5	0.2	1	79	85	89
Fludioxonil	f	4.1^a^	0.4	2	2	1	10	5	118	92	102
Flufenacet	h	3.5^a^	0.4	0.4	1	1	1	2	115	103	112
Flumethrin	a, i, bk	6.2^c^	n.a.^6^	2	25	n.a.^6^	5	40	n.a.^6^	89	103
Fluopyram	f	3.3^a^	n.a.^6^	0.4	0.5	5	2	2	133	111	112
Flupyradifurone	i	1.2^a^	0.4	0.4	1	1	1	2	126	116	109
Tau-fluvalinate	a, i, bk	7.0^a^	4	2	5	5	5	10	106	101	93
Hexythiazox	a, i	5.6^d^	2	0.8	2	2	2.5	5	86	90	87
Imidacloprid	i	0.6^a^	0.8	2	2	1	5	5	116	124	105
Indoxacarb	i	4.7^a^	0.8	0.4	2	2	5	5	101	101	110
Iprovalicarb	f	3.2^a^	2	0.8	1	2	1	5	99	96	129
Mandipropamid	f	3.2^a^	0.4	2	0.5	1	2	2	90	124	119
Mepanipyrim	f	3.3^a^	0.8	0.8	2	1	1	5	99	91	81
Metconazole	f	3.9^a^	0.4	2	1	0.5	5	2	88	107	98
Methoxyfenozide	i	3.7^a^	0.8	0.4	1	1	0.5	2	105	120	117
Permethrin	i	6.1^a^	2	4	10	5	5	40	94	102	103
Piperonyl butoxide	s	4.8^a^	0.2	2	0.5	0.5	2	1	112	108	127
Propoxur	a, i	1.5^b^	0.4	0.4	0.2	0.5	1	1	117	107	123
Prosulfocarb	h	4.5^a^	0.8	0.4	1	2	2	1	91	110	87
Desthio-prothioconazole	TP	3.0^a^	n.a.^6^	0.4	5	5	5	20	83	100	89
Pyraclostrobin	f	4.0^a^	0.8	0.4	0.2	1	2	1	107	108	128
Spinosad^e^	i	4.0^f^, 4.5^g^	2	2	2	5	5	5	76	89	103
Spirodiclofen	a, i	5.1^a^	2	2	10	5	2.5	20	93	94	68
Tebuconazole	f	3.7^a^	0.8	2	2	2	5	5	98	69	95
Terbuthylazine	h	3.4^a^	n.a.^6^	0.4	0.5	1	0.5	1	131	89	124
Thiacloprid	i	1.3^a^	n.a.^6^	2	1	5	5	2	126	100	109
Thiamethoxam	i	-0.1^a^	0.4	0.4	0.5	0.5	2	1	85	115	120
Trifloxystrobin	f	4.5^a^	1	n.a.^6^	n.a.^6^	2	5	2	100	103	125

^1^*a* acaricide, *i* insecticide, *f* fungicide, *s* synergist, *TP* transformation product, *bk* use as acaricide in beekeeping

^2^Octanol-water partition coefficient (log *K*_ow_) and the respective literature sources

^3^Limits of detection (LOD)

^4^Limits of quantification (LOQ)

^5^Recoveries (Rec) at the quantification limits

^6^Not available (n.a.)

^a^Pesticide Properties Database (PPDB), Lewis et al. (2016)

^b^https://pubchem.ncbi.nlm.nih.gov

^c^ https://www.fao.org/3/w5897e/w5897e2f.htm

^d^https://www.smolecule.com/products/s529927

^e^Spinosad composed of Spinosyn A and D (reference standard used 84:16)

^f^Spinosyn A: https://www3.epa.gov/pesticides/chem_search/reg_actions/registration/fs_PC-110003_19-Jul-99.pdf

^g^Spinosyn D: https://www3.epa.gov/pesticides/chem_search/reg_actions/registration/fs_PC-110003_19-Jul-99.pdf

### Determination of the loss of water-soluble pesticides due to purification of the beeswax

Blank beeswax was spiked with 51 pesticides (reference standards listed in supplementary material Section S1.1) at concentration levels of 500 µg/kg. Next, the wax was melted at 80 °C and shaken by hand for homogenization. For the quantitation of the pesticides before purification, 0.5 g of the spiked wax samples were extracted in triplicate and analyzed as described above. For the quantitation of the pesticides after purification, 5 g of wax was weighed in triplicate. Subsequently, each wax sample was wrapped in small bags of silk organza cloth before placing each bag in a separate beaker containing 50 mL of water at a temperature of 80 °C. The water was kept at 80 °C for 1 h before cooling to room temperature. Subsequently, the wax was collected from the surface of the water, melted once more at 80 °C for 10 min (without water; closed jar), shaken by hand for homogenization, followed by extraction and analysis by UHPLC-MS/MS.

To determine a possible loss of pesticides in the water during purification, the water was collected into 50-mL falcon tubes. An aliquot of 1 mL of water used for the purification of each wax sample was extracted with 4 mL of acetonitrile. Next, 0.2 g sodium chloride, 0.6 magnesium sulfate, 0.25 tri-sodium citrate dihydrate, and 0.12 g sodium hydrogencitrate sesquihydrate were added. The samples were shaken for 10 min. After centrifugation, the supernatants were filtered and analyzed by UHPLC-MS/MS.

## Results

### Method validation

Analytical methods based on the previously described procedure for bee bread (Schaad et al. 2023) were validated for the quantitation of 50 pesticides in pollen and 51 pesticides in beeswax. All matrices were tested for the same compounds while achieving different levels of sensitivity. The analytical procedures for pollen achieved high sensitivity, with LOQs ranging between 0.5 and 5 µg/kg for the quantitation of 42 pesticides, while the described methods were less sensitive for eight of the tested pesticides with LOQs between 10 and 100 µg/kg (Table 1). The recovery rates in pollen were all above 75% at the corresponding LOQ levels (Table 1). For bees wax, high sensitivity was achieved for the quantitation of 37 pesticides, with LOQs ranging between 1 and 5 µg/kg, while lower sensitivity was obtained for 14 of the tested pesticides, with LOQs ranging between 10 and 100 µg/kg (Table 1). The recovery rates in wax were all above 80% at the corresponding LOQ levels (except for acrinathrin and spirodiclofen, with recovery rates at LOQ levels of 69% and 68%, respectively; Table 1). We also included previously published results for pesticides in the matrix bee bread (Schaad et al. 2023) to complement the study of their occurrence in pollen and temporal distribution in beeswax.

### Prevalence of pesticides in pollen, bee bread, and beeswax

As shown in Table 2, 29 of the analyzed pesticides were quantifiable (> LOQ) in at least one sample. We quantitated (> LOQ) 23 of the tested pesticides in pollen, 26 in bee bread, and 20 in beeswax samples (Table 2). The pesticides azoxystrobin, boscalid, cyprodinil, difenoconazole, fludioxonil, flufenacet, fluopyram, indoxacarb, mandipropamid, metconazole, prosulfocarb, pyraclostrobin, tebuconazole, terbuthylazine, thiacloprid, and trifloxystrobin as well as the synergist piperonyl butoxide were quantifiable in samples of all the tested matrices (Table 2).

**Table 2:** Prevalence and maximal concentrations of pesticides in individual pollen, bee bread and wax samples

		Pollen		Bee bread		Wax	
Pesticide	Class	Prevalence ^1^ (%)	Max. conc.^2^ (μg/kg)	Prevalence ^1^ (%)	Max. conc.^2^ (μg/kg)	Prevalence ^1^ (%)	Max. conc.^2^ (μg/kg)
Acetamiprid	i	52	48.3	63	16.0	0	< LOQ
Aclonifen	h	13	20.7	8	11.4	0	< LOQ
Azoxystrobin	f	73	110.6	22	71.8	35	52.7
Boscalid	f	10	49.2	12	50.4	3	41.3
Coumaphos	a, i	0	< LOQ	0	< LOQ	2	2.8
Lambda-cyhalothrin	i	0	< LOQ	2	21.0	0	< LOQ
Cyproconazole	f	2	1.3	0	< LOQ	0	< LOQ
Cyprodinil	f	31	2212.7	28	1964.5	82	986.4
Difenoconazole	f	50	47.5	12	72.9	42	36.5
Dimethoate	a, i	0	< LOQ	3	1.2	2	3.4
Fenhexamid	f	0	< LOQ	2	11.9	0	< LOQ
(E)-Fenpyroximate	a, i	4	2.2	0	< LOQ	5	4.9
Fludioxonil	f	42	189.9	3	42.5	27	21.7
Flufenacet	h	2	1.1	3	8.9	2	3.1
Fluopyram	f	4	14.4	8	28.0	97	14.5
Indoxacarb	i	8	11.1	3	25.7	12	12.4
Iprovalicarb	f	0	< LOQ	2	2.5	0	< LOQ
Mandipropamid	f	50	9.4	32	32.9	42	24.4
Metconazole	f	10	10.0	2	5.1	10	3.3
Permethrin	i	0	< LOQ	8	21.0	0	< LOQ
Piperonyl butoxide	s	6	0.6	2	2.8	2	1.1
Prosulfocarb	h	27	71.9	67	38.2	97	21.6
Desthio-prothioconazole	TP	8	9.6	2	5.6	0	< LOQ
Pyraclostrobin	f	6	4.8	10	7.6	18	3.8
Spinosad	i	4	13.8	7	15.2	0	< LOQ
Tebuconazole	f	17	17.4	7	59.7	13	22.2
Terbuthylazine	h	23	9.5	38	25.9	70	5.2
Thiacloprid	i	6	69.4	20	36.6	8	3.0
Trifloxystrobin	f	21	23.0	15	38.3	75	21.1

^1^Prevalence of samples containing a pesticide above LOQ (%) calculated from 60 bee bread, 48 pollen, and 60 wax samples

^2^Maximal concentration (max. conc.) measured in the individual pollen, bee bread, and wax samples

### Time of pesticide entry into the bee colony

**Fig. 1:**
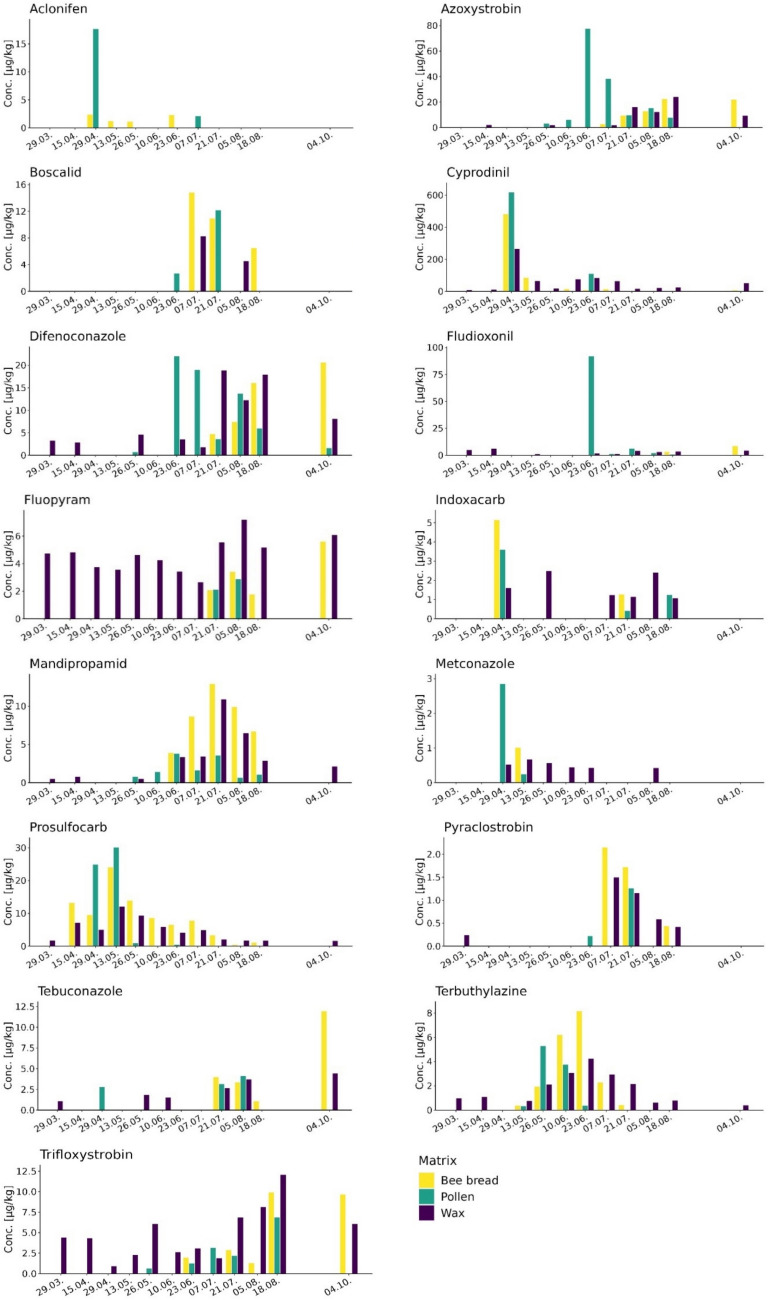
Temporal profiles of 15 lipophilic pesticides (log *K*_ow_ 2.5–4.7) with a minimal prevalence of 10% (values > LOQ) in one of the tested matrices. The *x*-axis shows the dates when the samples were taken during the year 2022, and the *y*-axis shows the mean concentrations of the five samples on a given sampling day representing a composite sample

**Fig. 2:**
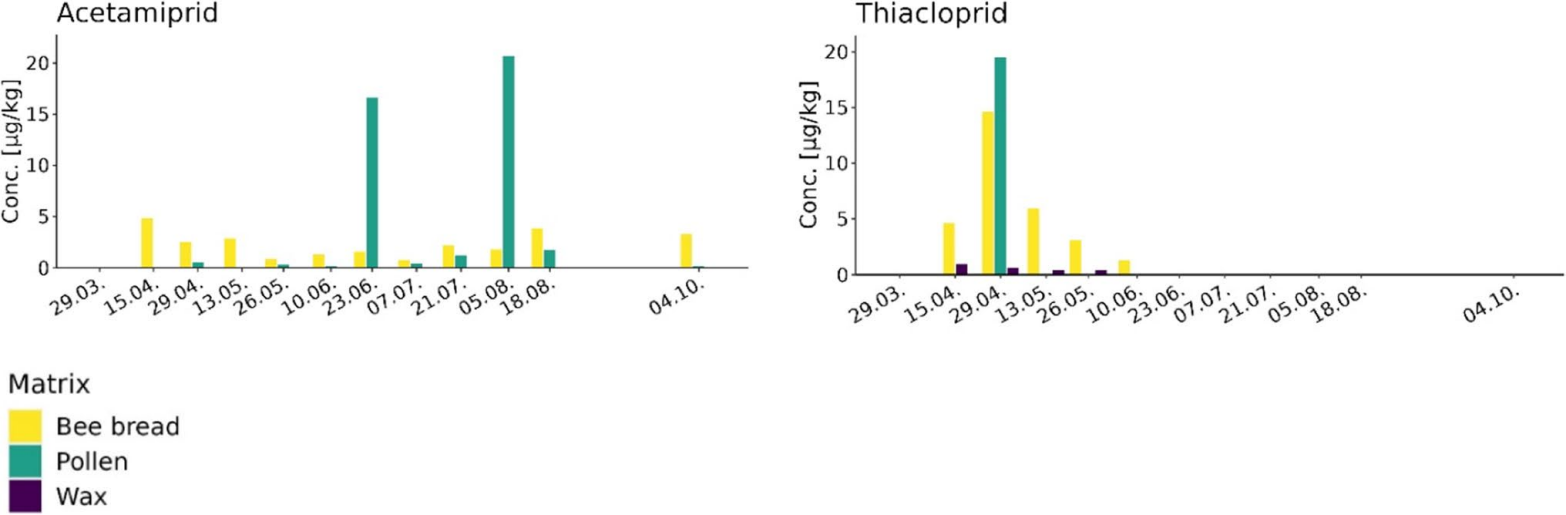
Temporal profiles of 2 hydrophilic pesticides (log *K*_ow_ 0.8; 1.3) with a minimal prevalence of 10% (values > LOQ) in one of the tested matrices. The x-axis shows the dates when the samples were taken during the year 2022, and the y-axis shows the mean concentrations of the five samples on a given sampling day

Figures 1 and 2 show the entry of the 17 most prevalent pesticides into the hive through pollen and their subsequent fate in beeswax. Multiple colonies were included in the study, since the types of pollen collected at a given date can vary considerably between different colonies of the same apiary (Keller et al. 2005) and, as a consequence, the type and concentrations of the pesticides collected by the bees (Schaad et al. 2023). Thus, the mean concentrations of the five individual samples collected each sampling day representing a composite sample were calculated for all the collection days throughout the season and are shown in Fig. 1 (lipophilic pesticides) and Fig. 2 (hydrophilic pesticides). The pesticides appeared in the hives at different points in time during the agricultural season and subsequently reached variable concentration levels in the sampled matrices. As shown in Fig. 1, aclonifen, cyprodinil, indoxacarb, and metconazole were quantified in pollen and bee bread (aclonifen, cyprodinil, and indoxacarb) on April 29, 2022. Other pesticides (e.g., azoxystrobin, difenoconazole, and trifloxystrobin) were quantified for the first time in pollen samples taken on May 26, 2022. Yet others (e.g., boscalid, fludioxonil, and pyraclostrobin) were quantified in pollen samples starting at the beginning of summer on June 23 or July 21, 2022 (fluopyram), most likely in relation to their application on crops. As shown in Fig. 2, two insecticides, acetamiprid and thiacloprid, were quantifiable for the first time in bee bread at the second sample date on April 15, while the highest concentrations were measured in pollen samples on April 29 (thiacloprid) and August 5, 2022 (acetamiprid).

### Temporal profiles of lipophilic pesticides

Pesticides were grouped according to their lipophilic properties. Figure 1 lists 15 prominent lipophilic pesticides with logarithmic octanol water coefficients (log *K*_ow_) between 2.5 and 4.7; thus, these pesticides have the potential to accumulate in beeswax. In fact, our study supports this hypothesis, as the levels of the lipophilic pesticides in beeswax correlated well with their appearance in pollen and/or bee bread (Fig. 1). For example, mandipropamid (log *K*_ow_ = 3.2) was brought into the hive through pollen starting at the end of May. The increasing concentrations in pollen/bee bread during June and July led to increasing concentrations in beeswax up to levels similar to those in bee bread. Other examples were difenoconazole, prosulfocarb, pyraclostrobin, or terbuthylazine, which demonstrated simultaneous increases in pesticide levels in pollen/bee bread and the levels in beeswax. Taken together, 14 of the 15 pesticides in Fig. 1 exhibited parallel temporal profiles of pesticide levels in pollen or bee bread and their profiles in beeswax. Increased or decreased concentrations of these pesticides in pollen or bee bread were followed by corresponding levels in beeswax. Aclonifen was not detected in wax, probably due to the fact that the analytical procedure was not sensitive enough (in wax LOQ = 100).

Cyprodinil, difenoconazole, fludioxonil, fluopyram, mandipropamid, prosulfocarb, pyraclostrobin, tebuconazole, terbuthylazine, and trifloxystrobin were already quantifiable in beeswax on March 29, 2022 (Fig. 1), exhibiting mean values between 0.2 and 8.3 µg/kg (Table S4). These residues in beeswax were most likely related to exposure in previous years, since the corresponding bee bread of these samples did not contain any of these pesticides above the LOQ. With the exception of pyraclostrobin, all these pesticides were still quantifiable in beeswax at the last sample date in October as well as during the subsequent agricultural season (Table S4). These results show that lipophilic pesticides in beeswax remain in beeswax beyond the agricultural production season.

### Temporal profiles of hydrophilic pesticides

**Fig. 3:**
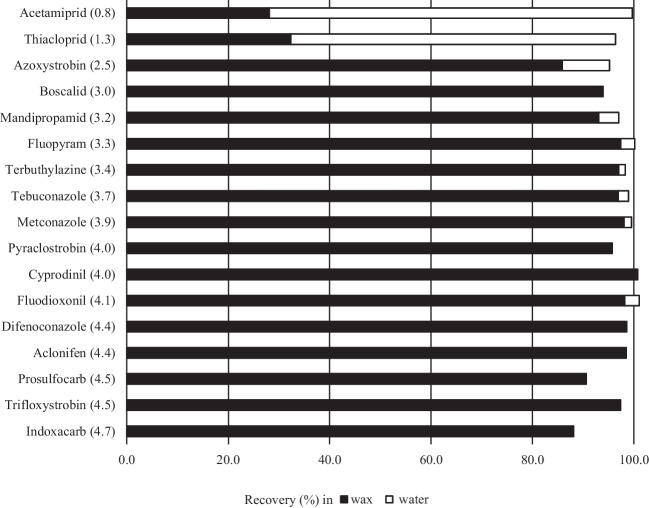
Ratio of pesticide in beeswax to water subsequent to wax purification in hot water. Recoveries (%) of the pesticides in beeswax are shown as black bars, while the recoveries (%) in water are listed as white bars. Pesticides were grouped according to their log *K*_ow_ shown in parentheses

Figure 2 lists two more hydrophilic pesticides, the neonicotinoids acetamiprid (log *K*_ow_ = 0.8) and thiacloprid (log *K*_ow_ = 1.3). These insecticides were most prevalent in pollen and bee bread, with mean maximal pollen concentrations close to 20 µg/kg (Fig. 2). Whereas acetamiprid was not detected in beeswax above the LOD of 1 µg/kg, a maximal mean concentration of 1.7 µg/kg was obtained for thiacloprid in beeswax on April 15, 2022 (Fig. 2), which was well below the concentrations in pollen, suggesting that these pesticides may migrate into beeswax to a lower extent than the more lipophilic pesticides. However, due to the purification process preceding the analytical extraction, to some extent, we underestimated their real concentration in beeswax (thiacloprid: 64%, acetamiprid: 72% loss into the water phase during purification; Fig. 3, Table S5).

### Prevalence of pesticides according to field of use and maximal concentrations in individual samples

The pesticides that were quantifiable (> LOQ) in at least one of the samples of any of the matrices included 6 insecticides, 3 acaricides/insecticides, 14 fungicides, 1 transformation product of a fungicide, 4 herbicides, and 1 synergist (Table 2).

In total, nine insecticides or acaricides/insecticides were quantifiable. The insecticide acetamiprid was found in 52% of the pollen (at maximal concentration in an individual sample of 48.3 µg/kg) and 63% of the bee bread samples, but it was not detected in the beeswax. Thiacloprid was most prevalent in bee bread (20%), while the highest concentration was measured in a pollen sample (69.4 µg/kg). In the beeswax, a maximal concentration of 3.0 µg/kg was measured. Indoxacarb was present in 8% of pollen samples, 3% of bee bread, and 12% of beeswax samples, with a maximal concentration of 25.7 µg/kg being measured in a bee bread sample. The prevalence values of the insecticides/acaricides (coumaphos, lambda-cyhalothrin, dimethoate, (E)-fenpyroxymate, permethrin, and spinosad) were below 10% in all of the tested matrices. Maximal concentrations of lambda-cyhalothrin (21.0 µg/kg), permethrin (21.0 µg/kg), and spinosad (15.2 µg/kg) were found in bee bread samples, while for coumaphos (2.8 µg/kg), dimethoate (3.4 µg/kg), and (E)-fenpyroxymate (4.9 µg/kg), the maximal concentration levels were in beeswax. Apart from low levels of coumaphos, acaricides with current authorization for beekeeping (flumethrin), previous authorization (bromopropylate*, tau*-fluvalinate), or the transformation product of amitraz (DMF; no authorization in Switzerland) were not detected.

We quantitated 14 fungicides and 1 transformation product of the fungicide prothioconazole. Of these fungicides, azoxystrobin, cyproconazole, difenoconazole, fludioxonil, mandipropamid, desthio-prothioconazole, and tebuconazole were most prevalent in pollen samples, while boscalid, fenhexamid, and iprovalicarb were most prevalent in bee bread. Metconazole was quantified equally frequently in pollen and beeswax. The remaining fungicides (cyprodinil, fluopyram, pyraclostrobin, and trifloxystrobin) were most prevalent in the beeswax samples. Maximal concentrations of azoxystrobin (110.6 µg/kg), cyprodinil (2212.7 µg/kg), and fludioxonil (189.9 µg/kg) were measured in individual pollen samples, while maximal concentrations of boscalid (50.4 µg/kg), difenoconazole (72.9 µg/kg), fluopyram (28.0 µg/kg), mandipropamid (32.9 µg/kg), tebuconazole (59.7 µg/kg), and trifloxystrobin (38.3 µg/kg) were measured in individual bee bread samples. For most fungicides, the maximal levels in beeswax were mostly in the same order of magnitude as the maximal levels in pollen or bee bread (Table 2).

All four of the analyzed herbicides were quantitated in at least one of the analyzed samples (Table 2). With a prevalence of 97% in beeswax, 67% in bee bread, and 27% in pollen, prosulfocarb was the most prevalent herbicide in the study at hand. Even though prosulfocarb was most prevalent in wax, the highest concentration was measured in pollen with 71.9 µg/kg. Terbuthylazine was also most prevalent in beeswax samples (70%), while its prevalence in pollen and bee bread was 23% and 38%, respectively. The remaining herbicides were less prevalent (prevalence of aclonifen in pollen 13%; prevalence of flufenacet in bee bread 3%). The highest concentrations of aclonifen (20.7 µg/kg) and flufenacet (8.9 µg/kg) were measured in the pollen and bee bread samples, respectively.

### Ability of the tested pesticides to remain in beeswax during wax purification

As shown in Fig. 3 and for additional pesticides in Table S5, lipophilic pesticides with a log *K*_ow_ above 2.5 remained in wax, with a recovery above 85% (except spirodiclofen and piperonyl butoxide) during the purification process in hot water. The pesticide loss into the water phase was below 10% for all tested pesticides with a log *K*_ow_ above 2.5 (Fig. 3; Table S5). The sum of the recoveries in wax and water was below 85% for two of the tested pesticides, spirodiclofen (42%) and piperonyl butoxide (67%), suggesting that these two pesticides might have been partially degraded during the process. By contrast, substantial loss into water was observed for pesticides with a lower log *K*_ow_, such as thiacloprid (log *K*_ow_ = 1.3) and acetamiprid (log *K*_ow_ = 0.8), for which 64% or 72% of the initial pesticide was lost during purification into the water phase (Fig. 3).

## Discussion

In the current study, we showed the entry route of a variety of plant protection products from pollen into beeswax and their fate over time. In a realistic field scenario, we revealed at what time point of the season pesticides in pollen are brought into the hive, to what extent they were stored in the bee bread, and how they eventually accumulated in the beeswax. Our experiments allowed us to study the detailed temporal profiles of a variety of pesticides throughout the agricultural season, which included insecticides, fungicides, and herbicides. Lipophilic pesticides demonstrated comparable temporal profiles in all tested matrices, with increased levels of pesticides in pollen and/or bee bread leading to increased levels in beeswax, while hydrophilic pesticides accumulated in beeswax to a much lower extent. Furthermore, melting wax in hot water showed that a large number of lipophilic pesticides remained in the purified beeswax. These pesticides have the potential to accumulate during the wax cycle in the beekeeping praxis, thus leading to the long-term exposure of honey bees to a multitude of contaminants in beeswax.

In total, 23 out of the 50 analyzed pesticides could be quantitated in pollen and 4 additional pesticides in bee bread from an apiary located in an agricultural environment. These pesticides (except spinosad) have previously been detected in bee bread or pollen collected in Germany (Deutsches Bienenmonitoring 2021; Friedle et al. 2021) and/or in pollen or bee bread from other European countries (Végh et al. 2021, 2023).

During April and May, the fungicides and insecticides may be related to oilseed rape cultivations or fruit trees and later in the season to maize, sunflower, and vegetable cultivations around the studied apiaries (Schaad et al. 2023). A large majority of the detected pesticides were lipophilic pesticides. In our study, 18 of the 23 pesticides quantitated in pollen were also found in beeswax, exposing bees to a variety of pesticides in beeswax. Our results show that lipophilic pesticides can remain in beeswax beyond the agricultural season (Fig. 1 and Table S4). Since pesticides were still present in autumn, the honey bees would have been exposed to a substantial variety of residues in the beeswax, not only during the crop season in spring and summer but also during winter, a period that is most critical for colony survival.

Increased brood mortality and delayed adult emergence have been previously observed in bees reared in combs containing high levels of a variety of pesticides (Wu et al. 2011). The pesticides investigated in previous studies were acaricides used in beekeeping. The significance of the exposure route through wax for bees has been shown for *tau*-fluvalinate, since bees accumulate pesticide in their bodies when exposed to *tau*-fluvalinate residues in wax (Fulton et al. 2019). Further, the transfer of several pesticides (*tau*-fluvalinate, coumaphos, and some transformation products of amitraz) from wax into bee brood has been previously described (Alkassab et al. 2022; Murcia Morales et al. 2020; Luna et al. 2023). Previous studies have also shown that in free-flying colonies, high coumaphos residue levels in foundation sheets above 60 mg/kg drastically increased brood mortality (Kast et al. 2023) and that coumaphos residues in wax affected queen development (Collins et al. 2004; Pettis et al. 2004). Furthermore, pesticides can also migrate from the wax into the larval jelly, thus exposing the developing bees orally to contaminated jelly, resulting in increased larval mortality, as previously demonstrated in an in vitro assay using the example of coumaphos (Kast and Kilchenmann 2022). So far, very little data is available on the impact of chronic exposure of honey bees to sub-lethal doses of pesticides in beeswax and on possible adverse synergistic effects of low levels of pesticides on honey bees (Wilmart et al. 2021). In this sense, further studies are still needed.

It is good beekeeping practice to exchange old brood combs after three to four years of use. In Switzerland, beekeepers usually recuperate wax from old combs for the production of new foundation sheets, which later serve as templates for bees to construct new combs. Lipophilic pesticides may withstand elevated temperatures during the wax recycling process and thus remain in commercial beeswax for decades, even if no longer in use, as previously shown for some acaricides, which were used as veterinary drugs for apiculture (Kast et al. 2021). Our study shows that this can be true for a large variety of agricultural pesticides. Subsequent to a purification process in hot water, simulating a possible recycling process performed by beekeepers, 38 out of 40 tested lipophilic pesticides with a (log *K*_ow_ ≥ 2.5) withstood the purification process as they remained in wax at comparable levels as before purification. Therefore, they are likely to be recycled together with wax and might also be present in newly produced foundation wax (Ostiguy et al. 2019; Mullin et al. 2010). In this sense, it is not surprising that similar levels of the lipophilic pesticides acrinathrin, chlorpyrifos, chlorfenvinphos coumaphos, fluvalinate, and flumethrin measured in the old comb were also found in newly produced foundation wax (Calatayud-Vernich et al. 2017). The continuous recycling of lipophilic compounds leads to a large number of pesticide residues in beeswax, thus constantly exposing honey bees to a large number of contaminants.

In contrast to lipophilic pesticides, hydrophilic pesticides tend to accumulate in the water during purification and are less likely to be present in new foundations at high concentration levels. In our study, 10 pesticides with log *K*_ow_ in the range of − 0.1 to 1.7 showed significant losses in the water phase. During wax purification, hydrophilic pesticides are mostly removed and washed into the water phase. This is in line with the observation that neonicotinoid insecticides (log *K*_ow_ of − 0.1 to 1.3), although very frequently measured in pollen (Végh et al. 2021), have been detected in beeswax only occasionally at low concentration levels (Végh et al. 2023).

For larvae and young honey bees, pollen or bee bread serves as a protein source. Newly emerged bees consume pollen to develop their muscles and glands, while nurse bees consume large quantities of pollen to produce protein-rich larval jelly (Keller et al. 2005). Larvae are better protected from the pesticides in pollen since they mostly consume jelly, which in general contains substantially fewer toxins (Lucchetti et al. 2018; Wueppenhorst et al. 2022).

Although adult honey bees mostly consume freshly collected pollen, part is stored as bee bread for later consumption when new pollen is not available. Approximately 70% of bee bread is consumed within the first 3 to 5 days and the remaining 30% within the next 2 to 3 weeks after collection (Roessink and van der Steen 2021). In this respect, it is not surprising that at any given time, the concentration levels of the pesticides in pollen and bee bread did not always match in our study. Especially early in the season when a lot of brood needs to be reared, pollen collected on a specific day might be immediately consumed. Thus, the collected pollen of a given day may not reflect the composition of the stored pollen as bee bread, which can represent a period of up to two weeks.

For the last 30 years, the Swiss Bee Research Center has monitored the levels of acaricides in beeswax (Kast et al. 2021). This project has been useful for the long-term observation of the use of acaricides as veterinary drugs in apiculture. It also allowed appropriate measures to be taken promptly, avoiding the critical concentration levels of acaricides in beeswax, which could have been problematic for honey bee health or honey quality (Kast et al. 2021). As our current study shows, most lipophilic pesticides were present in beeswax in the same order of magnitude as in pollen or bee bread. Therefore, beeswax can be an ideal matrix for monitoring not only acaricides but also pesticides used in agriculture. While pollen mirrors the immediate contamination level on a specific collection day, beeswax reflects a longer exposure and might also be more complete regarding the type of pesticides detected. A combination of pollen and beeswax might serve as an environmental monitoring program, allowing the monitoring of risk reduction measures with respect to the use of plant protection agents, as currently requested by policymakers (e.g., European Union 2020; Swiss Government 2017, 2021).

## Supplementary Information

Below is the link to the electronic supplementary material.

Supplementary file1 (DOCX 2.44 MB)

## Data Availability

The data generated during this study is included in this article and is also available from the corresponding author (christina.kast@agroscope.admin.ch) upon reasonable request. Samples are not available.
