# Supplementary material for: Temporal entry of pesticides through pollen into the bee hive and their fate in beeswax
Source: Environ Sci Pollut Res Int. 2024 Oct 15;31(51):61060–72. doi: 10.1007/s11356-024-35224-3 (PMC11534837; doi:10.1007/s11356-024-35224-3)

**Supplementary Material**

**Table S1** Liquid chromatography gradients for methods M1, M2 and M3 used for the quantitation of pesticides in pollen, bee bread and wax

|  | **Method M1** | | | **Method M2** | | | **Method M3** | | |
| --- | --- | --- | --- | --- | --- | --- | --- | --- | --- |
|  |  | **A**^a^  (v%) | **B^b^**  (v%) |  | **A**^a^  (v%) | **B^b^**  (v%) |  | **A**^a^  (v%) | **B^b^**  (v%) |
| 1 | 0.25 min | 100 | 0 | 0.25 min | 100 | 0 | 0.25 min | 100 | 0 |
| 2 | 6.60 min | 20 | 80 | 6.60 min | 0 | 100 | 4.00 min | 60 | 40 |
| 3 | 13.00 min | 0 | 100 | 12.00 min | 0 | 100 | 6.60 min | 0 | 100 |
| 4 | 13.01 min | 100 | 0 | 12.01 min | 100 | 0 | 9.01 min | 100 | 0 |

^a^ mobile phase A: 95% water + 5% acetonitrile + 0.01% formic acid + 5 mM ammonium formate

^b^ mobile phase B: 5% water + 95% acetonitrile + 0.01% formic acid + 5 mM ammonium formate

**Table S2** Ion source conditions of methods M1, M2 and M3 (pollen, bee bread and wax)

| **Method** | **M1** | **M2** | **M3** |
| --- | --- | --- | --- |
| Gas Temp. (°C) | 130 | 180 | 250 |
| Gas Flow (L/min) | 20 | 20 | 20 |
| Nebulizer (psi) | 30 | 30 | 60 |
| Sheath Gas Temp. (°C) | 120 | 150 | 320 |
| Sheath Gas Flow (L/min) | 10 | 6 | 10 |
| Capillary positive (V) | 6000 | 6000 | 6000 |
| Capillary negative (V) | - | 3500 | - |
| Nozzle voltage positive (V) | 2000 | 2000 | 2000 |
| Nozzle voltage negative (V) | - | 0 | - |

**Table S3:** Selected ion transitions used for qualification and quantification in each method.

|  |  |  | **Quantifier** |  | **Qualifier 1** |  | | **Qualifier 2** |  |
| --- | --- | --- | --- | --- | --- | --- | --- | --- | --- |
| **Pesticide** | **Method** | **Precursor  [m/z]** | **Product Ion  [m/z]** | **CE ^a^  [V]** | **Product Ion  [m/z]** | | **CE ^a^  [V]** | **Product Ion  [m/z]** | **CE ^a^  [V]** |
| Acetamiprid | M3 | 223.1 | 56.1 | 14 | 99 | | 46 | 90 | 42 |
| Aclonifen | M1 | 265 | 194 | 22 | 80 **^d,e^** | | 38 | 51.1 | 80 |
| Acrinathrin | M1 | 559.2 | 208.2 | 14 | 181.2 | | 38 | 83.2 | 18 |
| Azoxystrobin | M3 | 404.1 | 372.3 | 14 | 344.3 | | 26 | 329.3 | 34 |
| Azoxystrobin-D4 ^b^ | M3 | 408.1 | 348.1 | 26 | 376.3 **^d,e^** | | 14 | - | - |
| Bendiocarb | M3 | 224.1 | 167.2 | 6 | 109.1 | | 18 | 81.2 | 42 |
| Boscalid | M3 | 343 | 271.3 | 38 | 272.3 | | 34 | 140.1 **^e^** | 18 |
| Bromopropylate | M2 | 444 | 208.9 | 42 | 408.7 | | 6 | 152.9 | 66 |
| Chlorfenvinphos | M3 | 359 | 155.1 | 10 | 205.1 | | 22 | 170.1 | 50 |
| Chlorpyrifos | M1 | 349.9 | 198 | 18 | 125.1 | | 18 | 97.1 | 34 |
| Clothianidin | M3 | 250 | 131.9 | 18 | 113.0 **^f^** | | 30 | 110 **^f^** | 30 |
| Clothianidin-D3 | M1-M3 | 253 | 131.9 | 18 | - | | - | - | - |
| Coumaphos | M2 | 363 | 226.8 | 30 | 306.7 | | 18 | 210.8 | 34 |
| Lambda-cyhalothrin | M1 | 467.1 | 225 | 14 | 450.1 | | 6 | 141 | 58 |
| Zeta-cypermethrin | M1 | 433.1 | 191.1 | 14 | 416.3 | | 6 | 127.1 | 34 |
| Cyproconazole | M3 | 292.1 | 70 | 16 | 125 | | 32 | 89 | 60 |
| Cyproconazole-D3 **^b^** | M3 | 295.1 | 124.9 | 42 | - | | - | - | - |
| Cyprodinil | M3 | 226.1 | 108.0 | 30 | 77.0 **^d^** | | 66 | 39.1 | 80 |
| Deltamethrin | M1 | 521 | 279.1 | 14 | 504.1 | | 6 | 172.1 | 34 |
| Difenoconazole | M3 | 406.1 | 251 | 30 | 337 | | 18 | 188 | 54 |
| Dimethoate | M3 | 230 | 125 | 16 | 198.8 | | 0 | 79 | 32 |
| Dimoxystrobin | M3 | 327.2 | 205 | 10 | 238.1 | | 10 | 89 | 70 |
| DMF (Amitraz) | M3 M2 **^c^** | 150.1 | 106.9 | 22 | 106 **^d^** | | 38 | 77 | 50 |
| Fenhexamid | M3 | 302.1 | 97.1 | 26 | 55.1 | | 46 | 29.2 | 80 |
| Fenitrothion | M1 | 278 | 246.2 | 18 | 109.1 | | 18 | 125.1 **^e^** | 22 |
| (E)-Fenpyroximate | M3 | 422.2 | 366.4 | 18 | 138.1 | | 34 | 135.1 | 34 |
| Fipronil | M2 | 435 | 330 | 12 | 250 | | 28 | 183 | 40 |
| Fludioxonil | M1 | 266.1 | 229 | 6 | 185 | | 26 | 158 | 38 |
| Flufenacet | M1 | 364.1 | 152 | 22 | 194.1 **^e^** | | 10 | 77 | 80 |
| Flumethrin | M1 | 527.1 | 266.8 | 14 | 509.9 | | 6 | 238.8 | 22 |
| Fluopyram | M3 | 397.1 | 173 | 34 | 145 | | 70 | 95 | 80 |
| Fluopyram-D4 **^b^** | M3 | 401.1 | 177.0 | 30 | - | | - | - | - |
| Flupyradifurone | M3 | 289.1 | 126.1 | 22 | 90.1 | | 50 | 73.1 | 80 |
| Tau-fluvalinate | M2 | 503.1 | 180.9 | 38 | 207.9 **^e^** | | 10 | 151.9 | 80 |
| Hexythiazox | M3 | 353.1 | 227.9 | 8 | 168.1 **^d,e^** | | 24 | 151 | 32 |
| Imidacloprid | M3 | 256.1 | 209 | 14 | 175.1 **^d^** | | 18 | 84 | 22 |
| Indoxacarb | M3 | 528.1 | 218 | 22 | 161.9 | | 62 | 56.1 | 42 |
| Iprovalicarb | M3 | 321.2 | 119 | 16 | 91.1 | | 56 | 202.9 | 0 |
| Mandipropamid | M3 | 412.1 | 328.1 | 8 | 356.1 | | 4 | 125 | 40 |
| Mepanipyrim | M3 | 224.1 | 106.1 | 24 | 77 | | 40 | 209 | 36 |
| Metconazole | M3 | 320.1 | 70.1 | 24 | 125 | | 48 | 89 | 60 |
| Methoxyfenozide | M1 | 369.2 | 149 | 10 | 313.1 | | 0 | 133 | 24 |
| Permethrin | M1 | 408.1 | 183.2 | 6 | 355.3 | | 6 | 165.1 | 54 |
| Piperonyl butoxide | M3 | 356.2 | 177.2 | 14 | 119.2 | | 42 | 91.2 | 62 |
| Propoxur | M3 | 210.1 | 111.1 | 14 | 168.2 | | 2 | 93.1 | 26 |
| Prosulfocarb | M3 | 252.1 | 91.2 | 20 | 65.1 | | 60 | 43.1 | 16 |
| Desthio-prothioconazole | M3 | 312.1 | 70 | 20 | 125 | | 40 | 89.1 | 80 |
| Pyraclostrobin | M3 | 388.1 | 193.8 | 8 | 164.1 | | 12 | 163.1 | 20 |
| Spinosad (Spinosyn A) | M3 | 732.1 | 142.1 | 30 | 98.1 | | 80 | 97 | 74 |
| Spinosad (Spinosyn D) | M3 | 746.5 | 142 | 30 | 98.1 | | 80 | 97.3 | 78 |
| Spirodiclofen | M3 | 411 | 313.1 | 15 | 295 | | 35 | 71 **^d^** | 13 |
| Tebuconazole | M1 | 308.1 | 70 | 40 | 124.9 | | 47 | 59 | 36 |
| Terbuthylazine | M3 | 230.1 | 174.1 | 15 | 132 | | 25 | 104 | 35 |
| Terbuthylazine-D5 **^b^** | M3 | 235.1 | 179.0 | 18 | - | | - | - | - |
| Thiacloprid | M3 | 253 | 126.1 | 22 | 90.2 | | 46 | 73.1 | 78 |
| Thiacloprid-D4 **^b^** | M3 | 257.1 | 126.0 | 22 | - | | - | - | - |
| Thiamethoxam | M3 | 292 | 211.1 | 8 | 181.1 | | 20 | 132 | 24 |
| Trifloxystrobin | M3 | 409.1 | 186 | 12 | 206.1 | | 8 | 145 | 52 |

^a^Collision energy; ^b^Deuterated pesticides used for quantitation of the corresponding pesticides in wax and pollen (not in bee bread); ^c^Analysis of wax with method M2 and pollen and bee bread with method M3; ^d^Product ion used for quantitation in bee bread; ^e^Product ion used for quantitation in wax; ^f^Quantitation of clothianidin in bread with product ion 113.0 (m/z) and product ions 110.0 and 71.0 (m/z) used as qualifiers

| **Table S4**: Occurrence of pesticides in beeswax throughout the season tested in the same five colonies | | | | | | | | | | | | | | | | | | | | | | | | | | | | | | |  |  |  |  |  |  |
| --- | --- | --- | --- | --- | --- | --- | --- | --- | --- | --- | --- | --- | --- | --- | --- | --- | --- | --- | --- | --- | --- | --- | --- | --- | --- | --- | --- | --- | --- | --- | --- | --- | --- | --- | --- | --- |
|  | | | | | | | | | |  |  |  |  |  |  |  |  |  | |  | | | | |  | | | | |  |  |  |  |  |  |  |
| Pesticide | | | | | | | | | | spring (29.03.2022) | | | | maximal mean conc. (March to October 2022) | | | | | | autumn (04.10.2022) | | | | | | | | | | | | next season (21.04.2023) | | | |  |
|  | | | | | | | | | | Mean^1^ | SD^2^ | n^3^ >LOQ | n^4^ >LOD | Date | Mean^1^ | SD^2^ | n^3^ >LOQ | n^4^ >LOD | | Mean^1^ | | | | | SD^2^ | | | | | n^3^ >LOQ | n^4^ >LOD | Mean^1^ | SD^2^ | n^3^ >LOQ | n^4^ >LOD |  |
|  | | | | | | | | | | [µg/kg] | [µg/kg] | [µg/kg] | [µg/kg] |  | [µg/kg] | [µg/kg] | [µg/kg] | [µg/kg] | | [µg/kg] | | | | | [µg/kg] | | | | | [µg/kg] | [µg/kg] | [µg/kg] | [µg/kg] | [µg/kg] | [µg/kg] |  |
| Azoxystrobin | | | | | | | | | | n.d.^6^ |  |  |  | 18.08.2022 | **23.9** | 17.3 | 5 | n.a^7^ | | **9.2** | | | | | 4.5 | | | | | 5 | n.a^7^ | **21.9** | 25.5 | 3 | n.a^7^ |  |
| Boscalid | | | | | | | | | | n.d.^6^ |  |  |  | 07.07.2022 | **8.3** | 18.5 | 1 | 1 | | n.d.^6^ | | | | |  | | | | |  |  | n.d.^6^ |  |  |  |  |
| Cypronidil | | | | | | | | | | **8.3** | 10.2 | 3 | 2 | 29.04.2022 | **264.8** | 419.9 | 4 | 1 | | **49.6** | | | | | 57.1 | | | | | 4 | 1 | **18.7** | 28.2 | 4 | 1 |  |
| Difenconazole | | | | | | | | | | **3.2** | 4.5 | 2 | n.a^7^ | 21.07.2022 | **18.9** | 11.3 | 4 | n.a^7^ | | **8.1** | | | | | 6.4 | | | | | 4 | n.a^7^ | **19.7** | 23.0 | 3 | n.a^7^ |  |
| Fludioxonil | | | | | | | | | | 4.8 | 4.9 | 3 | 0 | 15.04.2022 | **6.0** | 5.8 | 3 | 0 | | **4.3** | | | | | 9.7 | | | | | 1 | 1 | **1.4** | 3.1 | 1 | 2 |  |
| Fluopyram | | | | | | | | | | **4.7** | 1.0 | 5 | 0 | 05.08.2022 | **7.2** | 4.6 | 5 | 0 | | **6.1** | | | | | 3.3 | | | | | 5 | 0 | **6.2** | 3.3 | 5 | 0 |  |
| Indoxacarb | | | | | | | | | | <LOQ ^5^ |  | 0 | 4 | 26.05.2022 | **2.5** | 5.6 | 1 | 2 | | n.d.^6^ | | | | |  | | | | |  |  | **2.5** | 3.5 | 2 | 1 |  |
| Mandipropamid | | | | | | | | | | **0.5** | 1.1 | 1 | 4 | 21.07.2022 | **10.9** | 8.1 | 5 | 0 | | **2.1** | | | | | 2.0 | | | | | 3 | 2 | **2.5** | 2.5 | 3 | 2 |  |
| Metconazol | | | | | | | | | | n.d.^6^ |  |  |  | 13.05.2022 | **0.7** | 1.5 | 1 | 2 | | <LOQ ^5^ | | | | |  | | | | | 0 | 1 | <LOQ ^5^ |  | 0 | 1 |  |
| Prosulfocarb | | | | | | | | | | **1.7** | 0.6 | 5 | 0 | 13.05.2022 | **12.1** | 5.5 | 5 | 0 | | **1.6** | | | | | 0.6 | | | | | 5 | 0 | **1.5** | 0.4 | 5 | 0 |  |
| Pyraclostrobin | | | | | | | | | | **0.2** | 0.5 | 1 | 3 | 07.07.2022 | **1.5** | 1.6 | 3 | 2 | | <LOQ ^5^ | | | | |  | | | | | 0 | 5 | <LOQ ^5^ |  | 0 | 3 |  |
| Tebuconazole | | | | | | | | | | **1.1** | 2.4 | 1 | 2 | 04.10.2022 | **4.4** | 9.9 | 1 | 2 | | **4.4** | | | | | 9.9 | | | | | 1 | 2 | **9.7** | 6.6 | 4 | 0 |  |
| Terbuthylazine | | | | | | | | | | **1.0** | 0.6 | 4 | 0 | 23.06.2022 | **4.2** | 0.8 | 5 | 0 | | **0.4** | | | | | 0.9 | | | | | 1 | 1 | **0.5** | 0.6 | 2 | 1 |  |
| Trifloxystrobin | | | | | | | | | | **4.4** | 1.4 | 5 | n.a^7^ | 18.08.2022 | **12.1** | 7.7 | 5 | n.a^7^ | | **6.1** | | | | | 3.9 | | | | | 5 | n.a^7^ | **12.1** | 12.4 | 4 | n.a^7^ |  |
| 1) Mean of 5 samples representing a composite sample; values <LOQs taken as 0 for the calculation of the mean  2) Standard deviation (SD)  3) Number (n) of samples (out of five samples analysed) above the limit of quantitation (LOQ)  4) Number (n) of samples (out of five samples analysed) above the limit of detection (LOD) and below the LOQ  5) Values of all samples below the limit of quantitation  6) Not detected (n.d.) in any of the samples  7) LOD not available (n.a.) | | | | | | | | | | | | | | | | | | | | | | | | | | | | | |  |  |  |  |  |  |  |
|  |  |  |  |  |  |  |  |  |  |  |  |  |  |  |  |  |  |  |  |  |  |  |  |  |  |  |  |  |  |  |  |  |  |  |  |  |
|  |  |  |  |  |  |  |  |  |  |  |  |  |  |  |  |  |  |  |  |  |  |  |  |  |  |  |  |  |  |  |  |  |  |  |  |  |
|  | | | | | | | | | | | | | | | | | | |  | |  |  |  |  |  |  |  |  |  |  |  |  |  |  |  |  |

**Table S5**: Proportion of pesticide remaining in wax after melting in water at 80°C

| **Pesticide** | **Log K*ow*** | **Percentage of** | **SD^1^** | **Percentage of** | **SD^1^** | **Sum of percentages** |
| --- | --- | --- | --- | --- | --- | --- |
|  |  | **pesticide remaining** |  | **pesticide** |  | **in wax and water** |
|  |  | **in wax** |  | **in water** |  |  |
|  |  | (%) | (%) | (%) | (%) | (%) |
| Tau-fluvalinate | 7.0 | 95.6 | 1.2 | n.d.^2^ |  | 96 |
| Zeta-cypermethrin | 6.6 | 96.7 | 1.9 | n.d.^2^ |  | 97 |
| Acrinathrin | 6.3 | 99.9 | 2.0 | n.d.^2^ |  | 100 |
| Flumethrin | 6.2 | 101.1 | 1.5 | n.d.^2^ |  | 101 |
| Permethrin | 6.1 | 99.5 | 1.9 | n.d.^2^ |  | 100 |
| (E)-Fenpyroximate | 5.7 | 95.0 | 0.7 | n.d.^2^ |  | 95 |
| Hexythiazox | 5.6 | 98.1 | 1.5 | n.d.^2^ |  | 98 |
| Lambda-cyhalothrin | 5.5 | 97.8 | 3.0 | n.d.^2^ |  | 98 |
| Bromopropylate | 5.4 | 94.9 | 4.1 | n.d.^2^ |  | 95 |
| Spirodiclofen | 5.1 | 42.4 | 4.9 | n.d.^2^ |  | 42 |
| Piperonyl butoxide | 4.8 | 66.7 | 3.8 | 0.2 | 0.01 | 67 |
| Chlorpyrifos | 4.7 | 99.3 | 0.3 | n.d.^2^ |  | 99 |
| Indoxacarb | 4.7 | 88.1 | 3.6 | n.d.^2^ |  | 88 |
| Deltamethrin | 4.6 | 101.7 | 1.2 | n.d.^2^ |  | 102 |
| Trifloxystrobin | 4.5 | 97.2 | 0.9 | 0.1 | 0.01 | 97 |
| Prosulfocarb | 4.5 | 90.6 | 1.0 | n.d.^2^ |  | 91 |
| Aclonifen | 4.4 | 98.5 | 3.2 | n.d.^2^ |  | 98 |
| Difenoconazole | 4.4 | 98.6 | 0.8 | n.d.^2^ |  | 99 |
| Desthio-prothioconazole | 4.3 | 98.5 | 1.1 | 2.8 | 0.2 | 101 |
| Coumaphos | 4.1 | 91.1 | 0.4 | 0.3 | 0.04 | 91 |
| Fludioxonil | 4.1 | 98.2 | 0.9 | 2.9 | 0.2 | 101 |
| Spinosad | 4.0 | 88.0 | 1.1 | n.d.^2^ |  | 88 |
| Cyprodinil | 4.0 | 100.7 | 0.9 | n.d.^2^ |  | 101 |
| Fipronil | 4.0 | 97.5 | 0.9 | 1.6 | 0.1 | 99 |
| Pyraclostrobin | 4.0 | 95.5 | 1.5 | 0.3 | 0.03 | 96 |
| Metconazole | 3.9 | 98.1 | 1.3 | 1.5 | 0.1 | 100 |
| Chlorfenvinphos | 3.8 | 97.8 | 1.7 | 1.0 | 0.0 | 99 |
| Methoxyfenozide | 3.7 | 96.0 | 1.4 | 3.0 | 0.2 | 99 |
| Tebuconazole | 3.7 | 96.9 | 1.2 | 2.1 | 0.2 | 99 |
| Dimoxystrobin | 3.6 | 97.3 | 0.6 | 2.2 | 0.1 | 99 |
| Fenhexamid | 3.5 | 98.8 | 5.3 | n.d.^2^ |  | 99 |
| Flufenacet | 3.5 | 97.0 | 0.2 | 2.2 | 0.2 | 99 |
| Terbuthylazine | 3.4 | 97.0 | 0.2 | 1.3 | 0.1 | 98 |
| Fenitrothion | 3.3 | 99.7 | 3.4 | n.d.^2^ |  | 100 |
| Fluopyram | 3.3 | 97.4 | 1.6 | 2.8 | 0.3 | 100 |
| Mepanipyrim | 3.3 | 100.9 | 0.7 | 0.9 | 0.1 | 102 |
| Iprovalicarb | 3.2 | 97.4 | 2.3 | 4.1 | 0.6 | 101 |
| Mandipropamid | 3.2 | 93.1 | 1.0 | 3.9 | 0.3 | 97 |
| Cyproconazole | 3.1 | 93.4 | 1.1 | 6.6 | 0.6 | 100 |
| Boscalid | 3.0 | 93.9 | 4.0 | n.d. |  | 94 |
| Azoxystrobin | 2.5 | 85.9 | 1.0 | 9.3 | 0.8 | 95 |
| Bendiocarb | 1.7 | 54.5 | 2.2 | 32.2 | 2.7 | 87 |
| DMF (Amitraz) | 1.5 | 47.9 | 2.1 | 49.9 | 3.8 | 98 |
| Propoxur | 1.5 | 53.6 | 1.9 | 46.3 | 3.8 | 100 |
| Thiacloprid | 1.3 | 32.3 | 4.5 | 64.0 | 5.4 | 96 |
| Flupyradifurone | 1.2 | 33.5 | 5.0 | 63.5 | 5.8 | 97 |
| Clothianidin | 0.9 | 31.2 | 4.7 | 66.7 | 5.4 | 98 |
| Acetamiprid | 0.8 | 28.1 | 4.7 | 71.6 | 6.0 | 100 |
| Dimethoate | 0.8 | 23.0 | 3.8 | 74.2 | 6.0 | 97 |
| Imidacloprid | 0.6 | 27.4 | 4.9 | 72.6 | 6.3 | 100 |
| Thiamethoxam | -0.1 | 29.5 | 4.5 | 71.7 | 7.5 | 101 |

^1^Standard deviation (SD) ^2^Not detected (n.d.)

***Section S1.1 Reference standards, solvents, and chemicals***

The following pesticides, used as standards, were purchased from LGC Standards GmbH (Wesel, Germany): Acetamiprid (C10013000), aclonifen (C10042000), azoxystrobin (C10413000), bendiocarb (C10460000), boscalid (C10663000), bromopropylate (C10762000), chlorfenvinphos (C11290000), chlorpyrifos (C11600000), clothianidin (C11691700), clothianidin-D3 (C11691710), lambda-cyhalothrin (C11860000), zeta-cypermethrin (C11890500), cyproconazole (C11908000), cyprodinil (C11909000), deltamethrin (C12120000), dimethoate (C12700000), N-(2,4-dimethylphenyl)formamide (DMF) (C12737000), difenoconazole (C12609000), dimoxystrobin (C12775000), fenhexamid (C13476000), fenitrothion (C13480000), (E)-fenpyroximate (C13545000), fipronil (C13645000), fludioxonil (C13705000), flufenacet (C13711000), tau-fluvalinate (C13870000), flumethrin (C13719000), fluopyram (C13743000), hexythiazox (C14210000), imidacloprid (C14283700), indoxacarb (C14325500), iprovalicarb (C14371000), mandipropamid (C14745000), mepanipyrim (C14867000), metconazole (C14955000), methoxyfenozide (C15080500), permethrin (C15990000), piperonyl butoxide (C16240000), propoxur (C16500000), prosulfocarb (C16545000), desthio-prothioconazole (C16555500), pyraclostrobin (C16595000), spirodiclofen (C16972950), tebuconazole (C17178700), terbuthylazine (C17300000), thiamethoxam (C17453000) and trifloxystrobin (C17842000). Acrinathrin (46415), azoxystrobin-D4 (51949), coumaphos (45403), cyproconazole-D3 (91796), fluopyram-D4 (06899), flupyradifurone (37050), terbuthylazine-D5 (91799), thiacloprid (37905), and thiacloprid-D4 (30673) were obtained from Merck (Darmstadt, Germany).

Acetonitrile SupraSolv (1.00017) was obtained from Merck (Darmstadt, Germany). Formic acid solution 50% for HPLC (09676) was purchased from Honeywell Fluka (Buchs, Switzerland) and ammonium formate (70221) was purchased from Merck (Darmstadt, Germany). Magnesium-sulfate (63136) and sodium hydrogencitrate sesquihydrate (359084) were obtained from Sigma-Aldrich (Buchs, Switzerland), sodium chloride (1.06404) and tri-sodium citrate dihydrate (1.06448.0500) from Merck (Darmstadt, Germany), and Bondesil PSA 40 µm (12213024) and Bondesil C18 40 µm (12213012) from Agilent Technologies (Santa Clara, California, USA). The 0.45 μm polyamide filters (729049) were purchased from Machery-Nagel (Düren, Germany). The water used for the mobile phases was purified with a Milli-Q IQ 7000 system.

***Section S1.2 Extraction of pesticides from pollen and wax***

Pollen (1 g) or bee bread (1 g) was weighed into a 50 mL centrifugation tube before 1 mL of MilliQ water was added. The pesticides were extracted with 4 mL of acetonitrile containing the internal standards (5 µg/L azoxystrobin-D4, 10 µg/L clothianidin-D3, 10 µg/L cyproconazole-D3, 5 µg/L fluopyram-D4, 5 µg/L terbuthylazine-D5 and 5 µg/L thiacloprid-D4). The tubes were shaken four times for 30 s by hand. Next, 0.2 g sodium chloride and 0.6 g magnesium sulfate were added to salt out the aqueous phase, as well as 0.25 tri-sodium citrate dihydrate and 0.12 g sodium hydrogencitrate sesquihydrate serving as buffers. The tubes were shaken for 10 min on a UNIMAX 2010 sample shaker by Heidolph (Schwabach, Germany). Lipids were frozen out by placing the tubes for 1 h in the freezer at -20°C before centrifugation (10000 g; 4°C; 20 min.). Next, 1 mL aliquots were purified with 50 mg Bondesil PSA and 50 mg Bondesil C18 (for most pesticides). Only Bondesil PSA (50 mg) was used for the extraction of cyprodinil and spinosad, and only Bondesil C18 (50 mg) was used for the extraction of fenhexamid and spirodiclofen (Schaad et al., 2023). After centrifugation (10000 g; room temperature; 20 min.), the supernatants were filtered into a 1.5 mL auto-sampler vial using a single-use polyamide filter with a pore size of 0.45 µm.

For extraction of pesticides in wax, 0.5 g wax was weighed into a 50 mL centrifugation tube. Acetonitrile (5 mL) containing the internal standards (5 µg/L azoxystrobin-D4, 10 µg/L clothianidin-D3, 10 µg/L cyproconazole-D3, 5 µg/L fluopyram-D4, 5 µg/L terbuthylazine-D5 and 5 µg/L thiacloprid-D4) was added before placing the tubes in a hot water bath at 80 °C for melting the wax. The subsequent step, in which the samples were vigorously shaken for 30 s and placed back in the hot water bath for 10 min, was repeated four times. The samples were then allowed to cool to room temperature before placing them at −20 °C overnight. After centrifugation (10000 g; 4°C; 20 min.), 1 mL of the supernatant was purified with 25 mg Bondesil PSA and 25 mg Bondesil C18 (for most pesticides). Alternatively, 1 mL of the supernatant was purified with Bondesil PSA (25 mg) only for the extraction of cyprodinil and spinosad or 1 mL of the supernatant with Bondesil C18 (25 mg) only for the extraction of fenhexamid and spirodiclofen. The tubes were mixed twice on a vortex stirrer for 30 s. Subsequently the tubes were placed overnight in a freezer at −20°C. After centrifugation (10000 g; 4°C; 20 min.), the supernatants were filtered into 1.5 mL vials using a polyamide filter with a pore size of 0.45 µm.

***Section S1.3 Recoveries of pesticides in pollen at various spiking levels***

|  | **Acetamiprid** | | | | |
| --- | --- | --- | --- | --- | --- |
| Spiking levels |  | mean | SD^2^ | SD^2^ | Recovery |
| (µg/kg) | n^1^ | (µg/kg) | (µg/kg) | (%) | (%) |
|  |  |  |  |  |  |
| 0.5 | 8 | 0.54 | 0.06 | 11.8 | 108.0 |
| 1 | 5 | 0.98 | 0.07 | 6.8 | 97.5 |
| 2 | 8 | 1.8 | 0.17 | 9.3 | 90.0 |
| 5 | 8 | 4.6 | 0.37 | 8.2 | 91.3 |
| 20 | 8 | 18.7 | 1.3 | 6.7 | 93.3 |
| 1’000 | 8 | 978.3 | 61.3 | 6.3 | 97.8 |

^1^ number of independent determinations including extraction (n)

^2^ standard deviation (SD)

|  | **Aclonifen** | | | | |
| --- | --- | --- | --- | --- | --- |
| Spiking levels |  | mean | SD^2^ | SD^2^ | Recovery |
| µg/kg | n^1^ | (µg/kg) | (µg/kg) | (%) | (%) |
|  |  |  |  |  |  |
| 5 | 8 | 5.5 | 0.79 | 14.3 | 110.2 |
| 20 | 8 | 20.8 | 2.0 | 9.7 | 103.8 |
| 1’000 | 8 | 1002.3 | 76.6 | 7.6 | 100.2 |

^1^ number of independent determinations including extraction (n)

^2^ standard deviation (SD)

|  | **Acrinathrin** | | | | |
| --- | --- | --- | --- | --- | --- |
| Spiking levels |  | mean | SD^2^ | SD^2^ | Recovery |
| µg/kg | n^1^ | (µg/kg) | (µg/kg) | (%) | (%) |
|  |  |  |  |  |  |
| 20 | 8 | 18.1 | 2.6 | 14.1 | 90.6 |
| 1’000 | 8 | 929.8 | 89.5 | 9.6 | 93.0 |

^1^ number of independent determinations including extraction (n)

^2^ standard deviation (SD)

|  | **Azoxystrobin** | | | | |
| --- | --- | --- | --- | --- | --- |
| Spiking levels |  | mean | SD^2^ | SD^2^ | Recovery |
| µg/kg | n^1^ | (µg/kg) | (µg/kg) | (%) | (%) |
|  |  |  |  |  |  |
| 1 | 5 | 1.1 | 0.14 | 13.3 | 106.2 |
| 2 | 8 | 1.8 | 0.15 | 8.5 | 88.1 |
| 5 | 8 | 4.4 | 0.28 | 6.5 | 87.1 |
| 20 | 8 | 18.3 | 1.6 | 8.6 | 91.5 |
| 1’000 | 8 | 956.5 | 129.8 | 13.6 | 95.6 |

^1^ number of independent determinations including extraction (n)

^2^ standard deviation (SD)

|  | **Bendiocarb** | | | | |
| --- | --- | --- | --- | --- | --- |
| Spiking levels |  | mean | SD^2^ | SD^2^ | Recovery |
| µg/kg | n^1^ | (µg/kg) | (µg/kg) | (%) | (%) |
|  |  |  |  |  |  |
| 0.5 | 8 | 0.60 | 0.03 | 4.7 | 120.4 |
| 1 | 5 | 1.0 | 0.03 | 3.0 | 104.4 |
| 2 | 8 | 1.8 | 0.12 | 6.5 | 90.0 |
| 5 | 8 | 4.4 | 0.40 | 9.1 | 87.3 |
| 20 | 8 | 18.9 | 1.2 | 6.6 | 94.4 |
| 1’000 | 8 | 1012.1 | 56.2 | 5.6 | 101.2 |

^1^ number of independent determinations including extraction (n)

^2^ standard deviation (SD)

|  | **Boscalid** | | | | |
| --- | --- | --- | --- | --- | --- |
| Spiking levels |  | mean | SD^2^ | SD^2^ | Recovery |
| µg/kg | n^1^ | (µg/kg) | (µg/kg) | (%) | (%) |
|  |  |  |  |  |  |
| 5 | 7 | 5.4 | 1.0 | 18.2 | 107.5 |
| 20 | 8 | 18.0 | 2.7 | 15.0 | 90.2 |
| 1’000 | 8 | 998.2 | 59.3 | 5.9 | 99.8 |

^1^ number of independent determinations including extraction (n)

^2^ standard deviation (SD)

|  | **Bromopropylate** | | | | |
| --- | --- | --- | --- | --- | --- |
| Spiking levels |  | mean | SD^2^ | SD^2^ | Recovery |
| µg/kg | n^1^ | (µg/kg) | (µg/kg) | (%) | (%) |
|  |  |  |  |  |  |
| 20 | 6 | 24.2 | 2.8 | 11.5 | 121.1 |
| 1’000 | 8 | 814.2 | 78.7 | 9.7 | 81.4 |

^1^ number of independent determinations including extraction (n)

^2^ standard deviation (SD)

|  | **Chlorfenvinphos** | | | | |
| --- | --- | --- | --- | --- | --- |
| Spiking levels |  | mean | SD^2^ | SD^2^ | Recovery |
| µg/kg | n^1^ | (µg/kg) | (µg/kg) | (%) | (%) |
|  |  |  |  |  |  |
| 1 | 5 | 1.0 | 0.14 | 13.4 | 103.8 |
| 2 | 8 | 1.7 | 0.14 | 8.2 | 86.6 |
| 5 | 8 | 4.2 | 0.41 | 9.8 | 83.6 |
| 20 | 8 | 18.2 | 1.7 | 9.3 | 91.0 |
| 1’000 | 8 | 923.8 | 72.0 | 7.8 | 92.4 |

^1^ number of independent determinations including extraction (n)

^2^ standard deviation (SD)

|  | **Chlorpyrifos** | | | | |
| --- | --- | --- | --- | --- | --- |
| Spiking levels |  | mean | SD^2^ | SD^2^ | Recovery |
| µg/kg | n^1^ | (µg/kg) | (µg/kg) | (%) | (%) |
|  |  |  |  |  |  |
| 20 | 8 | 20.2 | 1.7 | 8.2 | 101.1 |
| 1’000 | 8 | 913.1 | 65.0 | 7.1 | 91.3 |

^1^ number of independent determinations including extraction (n)

^2^ standard deviation (SD)

|  | **Clothianidin** | | | | |
| --- | --- | --- | --- | --- | --- |
| Spiking levels |  | mean | SD^2^ | SD^2^ | Recovery |
| µg/kg | n^1^ | (µg/kg) | (µg/kg) | (%) | (%) |
|  |  |  |  |  |  |
| 2 | 8 | 2.2 | 0.10 | 4.7 | 109.8 |
| 5 | 8 | 5.1 | 0.28 | 5.5 | 102.1 |
| 20 | 8 | 19.1 | 1.2 | 6.3 | 95.6 |
| 1’000 | 8 | 994.2 | 54.8 | 5.5 | 99.4 |

^1^ number of independent determinations including extraction (n)

^2^ standard deviation (SD)

|  | **Coumaphos** | | | | |
| --- | --- | --- | --- | --- | --- |
| Spiking levels |  | mean | SD^2^ | SD^2^ | Recovery |
| µg/kg | n^1^ | (µg/kg) | (µg/kg) | (%) | (%) |
|  |  |  |  |  |  |
| 2 | 8 | 2.0 | 0.27 | 13.6 | 98.3 |
| 5 | 8 | 4.3 | 0.36 | 8.3 | 86.8 |
| 20 | 8 | 17.4 | 1.3 | 7.2 | 87.0 |
| 1’000 | 8 | 947.8 | 81.2 | 8.6 | 94.8 |

^1^ number of independent determinations including extraction (n)

^2^ standard deviation (SD)

|  | **Lambda-cyhalothrin** | | | | |
| --- | --- | --- | --- | --- | --- |
| Spiking levels |  | mean | SD^2^ | SD^2^ | Recovery |
| µg/kg | n^1^ | (µg/kg) | (µg/kg) | (%) | (%) |
|  |  |  |  |  |  |
| 20 | 8 | 20.6 | 2.7 | 13.1 | 103.0 |
| 1’000 | 8 | 836.8 | 91.2 | 10.9 | 83.7 |

^1^ number of independent determinations including extraction (n)

^2^ standard deviation (SD)

|  | **Zeta-cypermethrin** | | | | |
| --- | --- | --- | --- | --- | --- |
| Spiking levels |  | mean | SD^2^ | SD^2^ | Recovery |
| µg/kg | n^1^ | (µg/kg) | (µg/kg) | (%) | (%) |
|  |  |  |  |  |  |
| 20 | 8 | 19.7 | 2.6 | 13.1 | 98.3 |
| 1’000 | 8 | 796.4 | 83.4 | 10.5 | 79.6 |

^1^ number of independent determinations including extraction (n)

^2^ standard deviation (SD)

|  | **Cyproconazole** | | | | |
| --- | --- | --- | --- | --- | --- |
| Spiking levels |  | mean | SD^2^ | SD^2^ | Recovery |
| µg/kg | n^1^ | (µg/kg) | (µg/kg) | (%) | (%) |
|  |  |  |  |  |  |
| 1 | 5 | 1.3 | 0.15 | 12.1 | 125.8 |
| 2 | 8 | 2.0 | 0.15 | 7.8 | 98.9 |
| 5 | 8 | 4.7 | 0.29 | 6.1 | 94.0 |
| 20 | 8 | 18.7 | 1.2 | 6.5 | 93.5 |
| 1’000 | 8 | 939.8 | 55.3 | 5.9 | 94.0 |

^1^ number of independent determinations including extraction (n)

^2^ standard deviation (SD)

|  | **Cyprodinil***** | | | | |
| --- | --- | --- | --- | --- | --- |
| Spiking levels |  | mean | SD^2^ | SD^2^ | Recovery |
| µg/kg | n^1^ | (µg/kg) | (µg/kg) | (%) | (%) |
|  |  |  |  |  |  |
| 2 | 7 | 2.0 | 0.37 | 18.3 | 100.3 |
| 5 | 8 | 3.9 | 0.44 | 11.2 | 77.8 |
| 20 | 8 | 15.2 | 1.4 | 9.1 | 75.9 |
| 1’000 | 8 | 795.9 | 82.0 | 10.3 | 79.6 |

***Extraction only with PSA

^1^ number of independent determinations including extraction (n)

^2^ standard deviation (SD)

|  | **Deltamethrin** | | | | |
| --- | --- | --- | --- | --- | --- |
| Spiking levels |  | mean | SD^2^ | SD^2^ | Recovery |
| µg/kg | n^1^ | (µg/kg) | (µg/kg) | (%) | (%) |
|  |  |  |  |  |  |
| 20 | 8 | 17.2 | 2.8 | 16.0 | 86.0 |
| 1’000 | 8 | 827.3 | 64.7 | 7.8 | 82.7 |

^1^ number of independent determinations including extraction (n)

^2^ standard deviation (SD)

|  | **Difenoconazole** | | | | |
| --- | --- | --- | --- | --- | --- |
| Spiking levels |  | mean | SD^2^ | SD^2^ | Recovery |
| µg/kg | n^1^ | (µg/kg) | (µg/kg) | (%) | (%) |
| 2 | 8 | 2.0 | 0.09 | 4.6 | 100.6 |
| 5 | 8 | 4.2 | 0.36 | 8.6 | 83.1 |
| 20 | 8 | 16.8 | 1.6 | 9.3 | 83.8 |
| 1’000 | 8 | 856.8 | 69.0 | 8.1 | 85.7 |

^1^ number of independent determinations including extraction (n)

^2^ standard deviation (SD)

|  | **Dimethoate** | | | | |
| --- | --- | --- | --- | --- | --- |
| Spiking levels |  | mean | SD^2^ | SD^2^ | Recovery |
| µg/kg | n^1^ | (µg/kg) | (µg/kg) | (%) | (%) |
|  |  |  |  |  |  |
| 1 | 5 | 1.1 | 0.06 | 5.5 | 110.5 |
| 2 | 8 | 1.9 | 0.09 | 4.7 | 94.9 |
| 5 | 8 | 4.6 | 0.41 | 9.0 | 91.0 |
| 20 | 8 | 18.6 | 1.4 | 7.5 | 93.0 |
| 1’000 | 8 | 987.1 | 51.5 | 5.2 | 98.7 |

^1^ number of independent determinations including extraction (n)

^2^ standard deviation (SD)

|  | **Dimoxystrobin** | | | | |
| --- | --- | --- | --- | --- | --- |
| Spiking levels |  | mean | SD^2^ | SD^2^ | Recovery |
| µg/kg | n^1^ | (µg/kg) | (µg/kg) | (%) | (%) |
|  |  |  |  |  |  |
| 0.5 | 8 | 0.57 | 0.05 | 9.2 | 114.5 |
| 1 | 5 | 1.0 | 0.03 | 3.0 | 100.8 |
| 2 | 8 | 1.7 | 0.08 | 4.8 | 85.0 |
| 5 | 8 | 4.2 | 0.31 | 7.4 | 83.6 |
| 20 | 8 | 17.6 | 1.0 | 5.5 | 88.1 |
| 1’000 | 8 | 947.5 | 97.8 | 10.3 | 94.8 |

^1^ number of independent determinations including extraction (n)

^2^ standard deviation (SD)

|  | **DMF (Amitraz)** | | | | |
| --- | --- | --- | --- | --- | --- |
| Spiking levels |  | mean | SD^2^ | SD^2^ | Recovery |
| µg/kg | n^1^ | (µg/kg) | (µg/kg) | (%) | (%) |
|  |  |  |  |  |  |
| 2 | 7 | 2.2 | 0.27 | 12.6 | 107.8 |
| 5 | 8 | 5.4 | 0.33 | 6.1 | 107.6 |
| 20 | 8 | 21.2 | 1.8 | 8.6 | 105.8 |
| 1’000 | 8 | 1106.5 | 63.4 | 5.7 | 110.6 |

^1^ number of independent determinations including extraction (n)

^2^ standard deviation (SD)

|  | **Fenhexamid **** | | | | |
| --- | --- | --- | --- | --- | --- |
| Spiking levels |  | mean | SD^2^ | SD^2^ | Recovery |
| µg/kg | n^1^ | (µg/kg) | (µg/kg) | (%) | (%) |
|  |  |  |  |  |  |
| 20 | 8 | 17.8 | 1.3 | 7.4 | 89.0 |
| 1’000 | 8 | 857.0 | 97.5 | 11.4 | 85.7 |

** Extraction only with C18

^1^ number of independent determinations including extraction (n)

^2^ standard deviation (SD)

|  | **Fenitrothion** | | | | |
| --- | --- | --- | --- | --- | --- |
| Spiking levels |  | mean | SD^2^ | SD^2^ | Recovery |
| µg/kg | n^1^ | (µg/kg) | (µg/kg) | (%) | (%) |
|  |  |  |  |  |  |
| 5 | 8 | 4.7 | 0.71 | 15.1 | 93.5 |
| 20 | 8 | 19.5 | 2.3 | 11.6 | 97.4 |
| 1’000 | 8 | 996.6 | 63.8 | 6.4 | 99.7 |

^1^ number of independent determinations including extraction (n)

^2^ standard deviation (SD)

|  | **(E)-Fenpyroximate** | | | | |
| --- | --- | --- | --- | --- | --- |
| Spiking levels |  | mean | SD^2^ | SD^2^ | Recovery |
| µg/kg | n^1^ | (µg/kg) | (µg/kg) | (%) | (%) |
|  |  |  |  |  |  |
| 1 | 5 | 0.90 | 0.03 | 3.8 | 90.4 |
| 2 | 8 | 1.5 | 0.08 | 5.1 | 74.3 |
| 5 | 8 | 3.6 | 0.32 | 8.9 | 71.8 |
| 20 | 8 | 15.4 | 1.1 | 7.2 | 77.0 |
| 1’000 | 8 | 811.5 | 61.2 | 7.5 | 81.1 |

^1^ number of independent determinations including extraction (n)

^2^ standard deviation (SD)

|  | **Fipronil** | | | | |
| --- | --- | --- | --- | --- | --- |
| Spiking levels |  | mean | SD^2^ | SD^2^ | Recovery |
| µg/kg | n^1^ | (µg/kg) | (µg/kg) | (%) | (%) |
|  |  |  |  |  |  |
| 0.5 | 8 | 0.39 | 0.05 | 11.6 | 78.9 |
| 1 | 5 | 0.85 | 0.07 | 8.2 | 85.0 |
| 2 | 8 | 1.7 | 0.19 | 11.2 | 85.5 |
| 5 | 8 | 4.3 | 0.40 | 9.2 | 86.4 |
| 20 | 8 | 18.4 | 1.3 | 7.3 | 92.1 |
| 1’000 | 8 | 862.2 | 67.5 | 7.8 | 86.2 |

^1^ number of independent determinations including extraction (n)

^2^ standard deviation (SD)

|  | **Fludioxonil** | | | | |
| --- | --- | --- | --- | --- | --- |
| Spiking levels |  | mean | SD^2^ | SD^2^ | Recovery |
| µg/kg | n^1^ | (µg/kg) | (µg/kg) | (%) | (%) |
|  |  |  |  |  |  |
| 1 | 5 | 1.2 | 0.16 | 13.4 | 117.7 |
| 2 | 7 | 2.2 | 0.21 | 9.2 | 112.2 |
| 5 | 8 | 5.5 | 0.50 | 9.1 | 109.5 |
| 20 | 8 | 22.5 | 1.8 | 8.2 | 112.4 |
| 1’000 | 8 | 1001.1 | 42.6 | 4.3 | 100.1 |

^1^ number of independent determinations including extraction (n)

^2^ standard deviation (SD)

|  | **Flufenacet** | | | | |
| --- | --- | --- | --- | --- | --- |
| Spiking levels |  | mean | SD^2^ | SD^2^ | Recovery |
| µg/kg | n^1^ | (µg/kg) | (µg/kg) | (%) | (%) |
|  |  |  |  |  |  |
| 1 | 5 | 1.1 | 0.17 | 14.5 | 114.6 |
| 2 | 8 | 1.9 | 0.16 | 8.1 | 95.7 |
| 5 | 8 | 4.8 | 0.38 | 8.0 | 95.9 |
| 20 | 8 | 19.9 | 1.5 | 7.4 | 99.7 |
| 1’000 | 8 | 1016.2 | 66.7 | 6.6 | 101.6 |

^1^ number of independent determinations including extraction (n)

^2^ standard deviation (SD)

|  | **Fluopyram** | | | | |
| --- | --- | --- | --- | --- | --- |
| Spiking levels |  | mean | SD^2^ | SD^2^ | Recovery |
| µg/kg | n^1^ | (µg/kg) | (µg/kg) | (%) | (%) |
|  |  |  |  |  |  |
| 5 | 8 | 6.6 | 1.2 | 17.8 | 132.9 |
| 20 | 8 | 19.7 | 1.7 | 8.4 | 98.6 |
| 1’000 | 8 | 961.4 | 84.2 | 8.8 | 96.1 |

^1^ number of independent determinations including extraction (n)

^2^ standard deviation (SD)

|  | **Flupyradifurone** | | | | |
| --- | --- | --- | --- | --- | --- |
| Spiking levels |  | mean | SD^2^ | SD^2^ | Recovery |
| µg/kg | n^1^ | (µg/kg) | (µg/kg) | (%) | (%) |
|  |  |  |  |  |  |
| 1 | 5 | 1.3 | 0.09 | 7.3 | 125.9 |
| 2 | 8 | 2.2 | 0.29 | 13.4 | 107.6 |
| 5 | 8 | 4.9 | 0.62 | 12.6 | 98.3 |
| 20 | 8 | 20.1 | 1.8 | 8.7 | 100.7 |
| 1’000 | 7 | 1060.0 | 108.0 | 10.2 | 106.0 |

^1^ number of independent determinations including extraction (n)

^2^ standard deviation (SD)

|  | **Tau-fluvalinate** | | | | |
| --- | --- | --- | --- | --- | --- |
| Spiking levels |  | mean | SD^2^ | SD^2^ | Recovery |
| µg/kg | n^1^ | (µg/kg) | (µg/kg) | (%) | (%) |
|  |  |  |  |  |  |
| 5 | 8 | 5.3 | 0.48 | 9.0 | 105.6 |
| 20 | 8 | 18.8 | 1.5 | 8.0 | 94.0 |
| 1’000 | 8 | 960.8 | 55.5 | 5.8 | 96.1 |

^1^ number of independent determinations including extraction (n)

^2^ standard deviation (SD)

|  | **Hexythiazox** | | | | |
| --- | --- | --- | --- | --- | --- |
| Spiking levels |  | mean | SD^2^ | SD^2^ | Recovery |
| µg/kg | n^1^ | (µg/kg) | (µg/kg) | (%) | (%) |
|  |  |  |  |  |  |
| 2 | 8 | 1.7 | 0.15 | 8.9 | 86.1 |
| 5 | 8 | 4.0 | 0.33 | 8.3 | 80.3 |
| 20 | 8 | 16.9 | 1.2 | 7.2 | 84.6 |
| 1’000 | 8 | 866.3 | 46.7 | 5.4 | 86.6 |

^1^ number of independent determinations including extraction (n)

^2^ standard deviation (SD)

|  | **Imidacloprid** | | | | |
| --- | --- | --- | --- | --- | --- |
| Spiking levels |  | mean | SD^2^ | SD^2^ | Recovery |
| µg/kg | n^1^ | (µg/kg) | (µg/kg) | (%) | (%) |
|  |  |  |  |  |  |
| 1 | 5 | 1.2 | 0.12 | 10.5 | 115.6 |
| 2 | 8 | 1.8 | 0.17 | 9.4 | 90.1 |
| 5 | 8 | 4.3 | 0.39 | 8.9 | 86.4 |
| 20 | 8 | 17.4 | 1.5 | 8.8 | 87.1 |
| 1’000 | 8 | 940.6 | 66.9 | 7.1 | 94.1 |

^1^ number of independent determinations including extraction (n)

^2^ standard deviation (SD)

|  | **Indoxacarb** | | | | |
| --- | --- | --- | --- | --- | --- |
| Spiking levels |  | mean | SD^2^ | SD^2^ | Recovery |
| µg/kg | n^1^ | (µg/kg) | (µg/kg) | (%) | (%) |
|  |  |  |  |  |  |
| 2 | 8 | 2.0 | 0.25 | 12.5 | 101.0 |
| 5 | 8 | 4.7 | 0.52 | 11.1 | 93.3 |
| 20 | 8 | 19.1 | 1.7 | 8.7 | 95.6 |
| 1’000 | 8 | 985.6 | 82.6 | 8.4 | 98.6 |

^1^ number of independent determinations including extraction (n)

^2^ standard deviation (SD)

|  | **Iprovalicarb** | | | | |
| --- | --- | --- | --- | --- | --- |
| Spiking levels |  | mean | SD^2^ | SD^2^ | Recovery |
| µg/kg | n^1^ | (µg/kg) | (µg/kg) | (%) | (%) |
|  |  |  |  |  |  |
| 2 | 8 | 2.0 | 0.23 | 11.6 | 98.9 |
| 5 | 8 | 4.6 | 0.53 | 11.6 | 91.7 |
| 20 | 8 | 18.9 | 1.9 | 9.9 | 94.7 |
| 1’000 | 8 | 945.3 | 66.7 | 7.1 | 94.5 |

^1^ number of independent determinations including extraction (n)

^2^ standard deviation (SD)

|  | **Mandipropamid** | | | | |
| --- | --- | --- | --- | --- | --- |
| Spiking levels |  | mean | SD^2^ | SD^2^ | Recovery |
| µg/kg | n^1^ | (µg/kg) | (µg/kg) | (%) | (%) |
|  |  |  |  |  |  |
| 1 | 5 | 0.90 | 0.13 | 14.7 | 90.3 |
| 2 | 8 | 1.8 | 0.21 | 11.9 | 89.1 |
| 5 | 8 | 4.3 | 0.56 | 12.9 | 86.3 |
| 20 | 8 | 18.9 | 1.8 | 9.4 | 94.5 |
| 1’000 | 8 | 915.0 | 77.8 | 8.5 | 91.5 |

^1^ number of independent determinations including extraction (n)

^2^ standard deviation (SD)

|  | **Mepanipyrim** | | | | |
| --- | --- | --- | --- | --- | --- |
| Spiking levels |  | mean | SD^2^ | SD^2^ | Recovery |
| µg/kg | n^1^ | (µg/kg) | (µg/kg) | (%) | (%) |
|  |  |  |  |  |  |
| 1 | 5 | 0.99 | 0.14 | 14.1 | 99.4 |
| 2 | 8 | 1.7 | 0.21 | 12.2 | 87.4 |
| 5 | 8 | 4.1 | 0.50 | 12.1 | 82.7 |
| 20 | 8 | 17.7 | 1.5 | 8.4 | 88.4 |
| 1’000 | 8 | 873.3 | 63.9 | 7.3 | 87.3 |

^1^ number of independent determinations including extraction (n)

^2^ standard deviation (SD)

|  | **Metconazole** | | | | |
| --- | --- | --- | --- | --- | --- |
| Spiking levels |  | mean | SD^2^ | SD^2^ | Recovery |
| µg/kg | n^1^ | (µg/kg) | (µg/kg) | (%) | (%) |
|  |  |  |  |  |  |
| 0.5 | 8 | 0.44 | 0.04 | 8.7 | 88.3 |
| 1 | 5 | 0.88 | 0.06 | 7.2 | 87.5 |
| 2 | 8 | 1.6 | 0.16 | 10.2 | 80.5 |
| 5 | 8 | 4.0 | 0.41 | 10.4 | 79.5 |
| 20 | 8 | 18.2 | 1.7 | 9.4 | 90.8 |
| 1’000 | 8 | 832.7 | 61.0 | 7.3 | 83.3 |

^1^ number of independent determinations including extraction (n)

^2^ standard deviation (SD)

|  | **Methoxyfenozide** | | | | |
| --- | --- | --- | --- | --- | --- |
| Spiking levels |  | mean | SD^2^ | SD^2^ | Recovery |
| µg/kg | n^1^ | (µg/kg) | (µg/kg) | (%) | (%) |
|  |  |  |  |  |  |
| 1 | 5 | 1.0 | 0.09 | 8.9 | 104.6 |
| 2 | 8 | 1.9 | 0.18 | 9.7 | 93.7 |
| 5 | 8 | 4.4 | 0.44 | 10.1 | 88.2 |
| 20 | 8 | 18.6 | 1.4 | 7.6 | 92.9 |
| 1’000 | 8 | 965.3 | 86.0 | 8.9 | 96.5 |

^1^ number of independent determinations including extraction (n)

^2^ standard deviation (SD)

|  | **Permethrin** | | | | |
| --- | --- | --- | --- | --- | --- |
| Spiking levels |  | mean | SD^2^ | SD^2^ | Recovery |
| µg/kg | n^1^ | (µg/kg) | (µg/kg) | (%) | (%) |
|  |  |  |  |  |  |
| 5 | 8 | 4.7 | 0.57 | 12.1 | 94.1 |
| 20 | 8 | 18.0 | 1.3 | 7.2 | 89.9 |
| 1’000 | 8 | 939.9 | 33.7 | 3.6 | 94.0 |

^1^ number of independent determinations including extraction (n)

^2^ standard deviation (SD)

|  | **Piperonyl butoxide** | | | | |
| --- | --- | --- | --- | --- | --- |
| Spiking levels |  | mean | SD^2^ | SD^2^ | Recovery |
| µg/kg | n^1^ | (µg/kg) | (µg/kg) | (%) | (%) |
|  |  |  |  |  |  |
| 0.5 | 8 | 0.56 | 0.06 | 10.6 | 111.8 |
| 1 | 5 | 1.0 | 0.05 | 5.3 | 100.3 |
| 2 | 8 | 1.7 | 0.16 | 9.5 | 85.5 |
| 5 | 8 | 4.0 | 0.33 | 8.3 | 80.4 |
| 20 | 8 | 16.7 | 0.94 | 5.6 | 83.6 |

^1^ number of independent determinations including extraction (n)

^2^ standard deviation (SD)

|  | **Propoxur** | | | | |
| --- | --- | --- | --- | --- | --- |
| Spiking levels |  | mean | SD^2^ | SD^2^ | Recovery |
| µg/kg | n^1^ | (µg/kg) | (µg/kg) | (%) | (%) |
|  |  |  |  |  |  |
| 0.5 | 8 | 0.59 | 0.04 | 7.2 | 117.1 |
| 1 | 5 | 1.0 | 0.02 | 2.2 | 104.0 |
| 2 | 8 | 1.8 | 0.11 | 5.7 | 92.2 |
| 5 | 8 | 4.3 | 0.59 | 13.8 | 85.5 |
| 20 | 8 | 18.8 | 1.4 | 7.5 | 94.0 |
| 1’000 | 8 | 1028.5 | 57.2 | 5.6 | 102.9 |

^1^ number of independent determinations including extraction (n)

^2^ standard deviation (SD)

|  | **Prosulfocarb** | | | | |
| --- | --- | --- | --- | --- | --- |
| Spiking levels |  | mean | SD^2^ | SD^2^ | Recovery |
| µg/kg | n^1^ | (µg/kg) | (µg/kg) | (%) | (%) |
|  |  |  |  |  |  |
| 2 | 8 | 1.8 | 0.22 | 12.1 | 90.6 |
| 5 | 8 | 4.3 | 0.42 | 9.6 | 86.6 |
| 20 | 8 | 17.8 | 1.1 | 6.3 | 88.9 |
| 1’000 | 8 | 890.3 | 54.8 | 6.2 | 89.0 |

^1^ number of independent determinations including extraction (n)

^2^ standard deviation (SD)

|  | **Desthio-prothioconazole** | | | | |
| --- | --- | --- | --- | --- | --- |
| Spiking levels |  | mean | SD^2^ | SD^2^ | Recovery |
| µg/kg | n^1^ | (µg/kg) | (µg/kg) | (%) | (%) |
|  |  |  |  |  |  |
| 5 | 8 | 4.1 | 0.52 | 12.7 | 82.5 |
| 20 | 8 | 18.2 | 1.2 | 6.9 | 91.0 |

^1^ number of independent determinations including extraction (n)

^2^ standard deviation (SD)

|  | **Pyraclostrobin** | | | | |
| --- | --- | --- | --- | --- | --- |
| Spiking levels |  | mean | SD^2^ | SD^2^ | Recovery |
| µg/kg | n^1^ | (µg/kg) | (µg/kg) | (%) | (%) |
|  |  |  |  |  |  |
| 1 | 5 | 1.1 | 0.06 | 5.3 | 106.6 |
| 2 | 8 | 1.8 | 0.13 | 7.6 | 88.7 |
| 5 | 8 | 4.2 | 0.43 | 10.2 | 83.8 |
| 20 | 8 | 17.8 | 1.6 | 9.1 | 89.2 |
| 1’000 | 8 | 914.9 | 59.9 | 6.5 | 91.5 |

^1^ number of independent determinations including extraction (n)

^2^ standard deviation (SD)

|  |  | **Spinosyn A ***** | | | | |
| --- | --- | --- | --- | --- | --- | --- |
| Spinosad | Spinosyn A Spiking levels |  | mean | SD^2^ | SD^2^ | Recovery |
| µg/kg | (µg/kg) | n^1^ | (µg/kg) | (µg/kg) | (%) | (%) |
|  |  |  |  |  |  |  |
| 5 | 4.2 | 8 | 3.2 | 0.37 | 11.6 | 75.7 |
| 20 | 16.8 | 8 | 12.6 | 1.1 | 8.5 | 75.2 |
| 1’000 | 840 | 8 | 682.4 | 61.7 | 9.0 | 81.2 |

***Extraction only with PSA; Spinosad Lot No G1100667 corresponds to 84% Spinosyn A

^1^ number of independent determinations including extraction (n)

^2^ standard deviation (SD)

|  |  | **Spinosyn D ***** | | | | |
| --- | --- | --- | --- | --- | --- | --- |
| Spinosad | Spinosyn D Spiking levels |  | mean | SD^2^ | SD^2^ | Recovery |
| µg/kg | µg/kg | n^1^ | (µg/kg) | (µg/kg) | (%) | (%) |
|  |  |  |  |  |  |  |
| 5 | 0.8 | 8 | 0.65 | 0.06 | 9.7 | 81.8 |
| 20 | 3.2 | 8 | 2.4 | 0.18 | 7.6 | 75.9 |
| 1’000 | 160 | 8 | 127.8 | 10.9 | 8.5 | 79.9 |

***Extraction only with PSA; Spinosad Lot No G1100667 corresponds to 16% Spinosyn D

^1^ number of independent determinations including extraction (n)

^2^ standard deviation (SD)

|  | **Spirodiclofen **** | | | | |
| --- | --- | --- | --- | --- | --- |
| Spiking levels |  | mean | SD^2^ | SD^2^ | Recovery |
| µg/kg | n^1^ | (µg/kg) | (µg/kg) | (%) | (%) |
|  |  |  |  |  |  |
| 5 | 6 | 4.6 | 0.30 | 6.5 | 92.6 |
| 20 | 8 | 15.7 | 2.3 | 14.8 | 78.6 |
| 1’000 | 8 | 836.3 | 64.8 | 7.8 | 83.6 |

******Extraction only with C18

^1^ number of independent determinations including extraction (n)

^2^ standard deviation (SD)

|  | **Tebuconazole** | | | | |
| --- | --- | --- | --- | --- | --- |
| Spiking levels |  | mean | SD^2^ | SD^2^ | Recovery |
| µg/kg | n^1^ | (µg/kg) | (µg/kg) | (%) | (%) |
|  |  |  |  |  |  |
| 2 | 8 | 2.0 | 0.24 | 12.4 | 97.9 |
| 5 | 8 | 4.6 | 0.49 | 10.5 | 92.6 |
| 20 | 8 | 18.8 | 1.4 | 7.3 | 93.9 |
| 1’000 | 8 | 936.8 | 84.9 | 9.1 | 93.7 |

^1^ number of independent determinations including extraction (n)

^2^ standard deviation (SD)

|  | **Terbuthylazine** | | | | |
| --- | --- | --- | --- | --- | --- |
| Spiking levels |  | mean | SD^2^ | SD^2^ | Recovery |
| µg/kg | n^1^ | (µg/kg) | (µg/kg) | (%) | (%) |
|  |  |  |  |  |  |
| 1 | 5 | 1.3 | 0.21 | 15.8 | 131.1 |
| 2 | 8 | 2.0 | 0.17 | 8.6 | 100.3 |
| 5 | 8 | 4.5 | 0.37 | 8.1 | 90.1 |
| 20 | 8 | 18.0 | 0.86 | 4.8 | 89.8 |
| 1’000 | 8 | 904.2 | 49.9 | 5.5 | 90.4 |

^1^ number of independent determinations including extraction (n)

^2^ standard deviation (SD)

|  | **Thiacloprid** | | | | |
| --- | --- | --- | --- | --- | --- |
| Spiking levels |  | mean | SD^2^ | SD^2^ | Recovery |
| µg/kg | n^1^ | (µg/kg) | (µg/kg) | (%) | (%) |
|  |  |  |  |  |  |
| 5 | 8 | 6.3 | 1.2 | 19.4 | 125.9 |
| 20 | 8 | 20.3 | 1.4 | 6.8 | 101.3 |
| 1’000 | 8 | 988.1 | 56.0 | 5.7 | 98.8 |

^1^ number of independent determinations including extraction (n)

^2^ standard deviation (SD)

|  | **Thiamethoxam** | | | | |
| --- | --- | --- | --- | --- | --- |
| Spiking levels |  | mean | SD^2^ | SD^2^ | Recovery |
| µg/kg | n^1^ | (µg/kg) | (µg/kg) | (%) | (%) |
|  |  |  |  |  |  |
| 0.5 | 8 | 0.43 | 0.03 | 6.4 | 85.3 |
| 1 | 5 | 0.90 | 0.04 | 4.1 | 89.7 |
| 2 | 8 | 1.7 | 0.14 | 8.1 | 83.9 |
| 5 | 8 | 4.4 | 0.41 | 9.4 | 87.0 |
| 20 | 8 | 18.5 | 1.3 | 7.2 | 92.6 |
| 1’000 | 8 | 956.3 | 67.4 | 7.0 | 95.6 |

^1^ number of independent determinations including extraction (n)

^2^ standard deviation (SD)

|  | **Trifloxystrobin** | | | | |
| --- | --- | --- | --- | --- | --- |
| Spiking levels |  | mean | SD^2^ | SD^2^ | Recovery |
| µg/kg | n^1^ | (µg/kg) | (µg/kg) | (%) | (%) |
|  |  |  |  |  |  |
| 2 | 8 | 2.0 | 0.16 | 8.2 | 100.3 |
| 5 | 8 | 4.4 | 0.38 | 8.6 | 88.2 |
| 20 | 8 | 18.6 | 1.6 | 8.7 | 92.9 |
| 1’000 | 8 | 952.0 | 86.7 | 9.1 | 95.2 |

^1^ number of independent determinations including extraction (n)

^2^ standard deviation (SD)

***Section S1.4*** **Multiresidue chromatograms**

Methods M1, M2, M3 for pesticides (20 µg/kg) in blank pollen extract

(Agilent MassHunter Qualitative Analyse)

**Method M1**


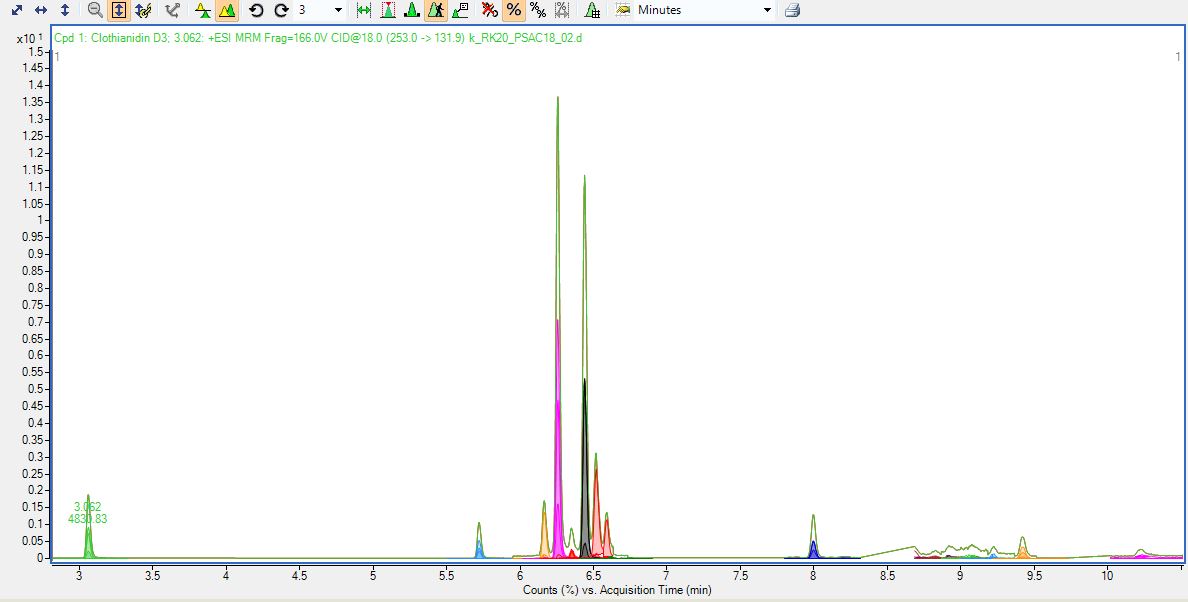


**Method M2**


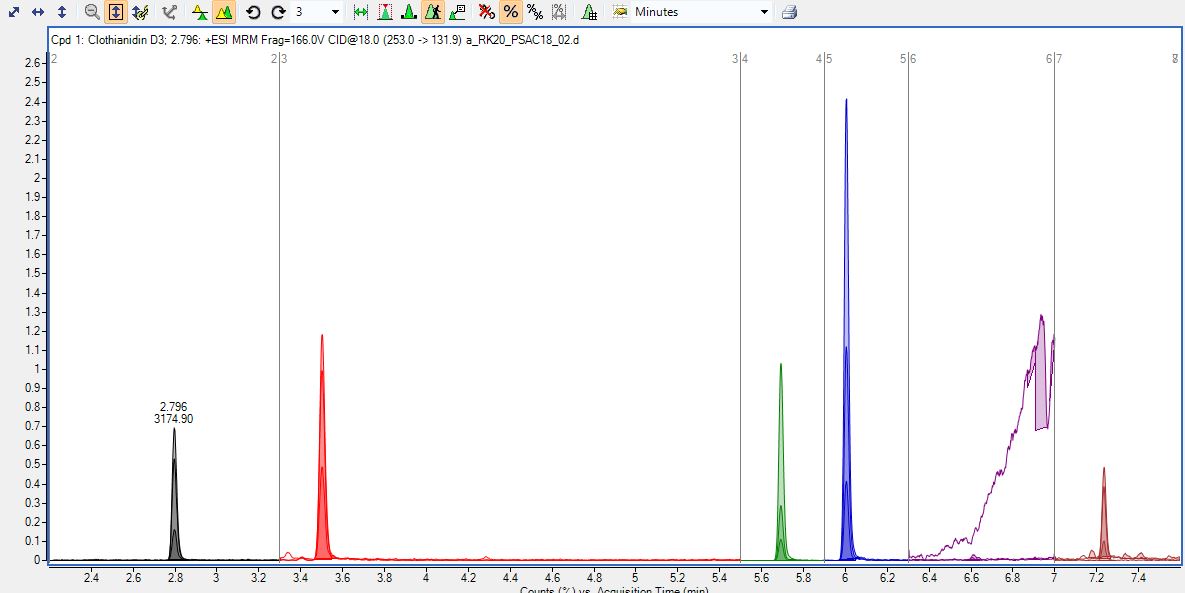


**Method M3**


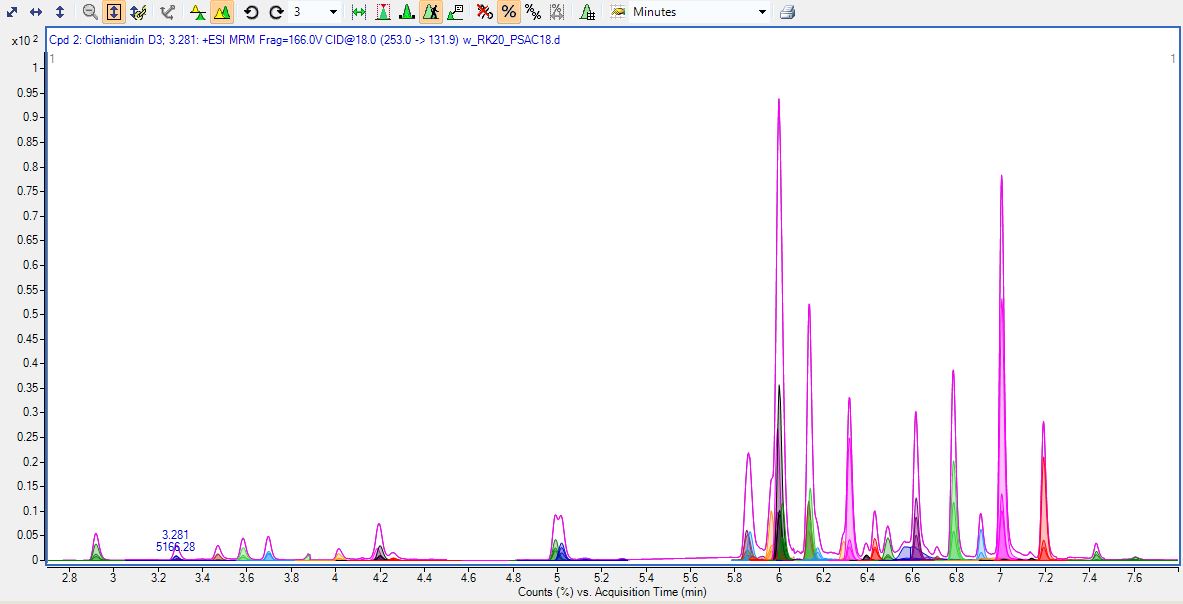


***Section S1.5 Identification and quantitation of pesticides in blank pollen extract***

(QQQ Quantitative Analysis, Quant-My-Way)

Acetamiprid (method M3) 20 µg/kg


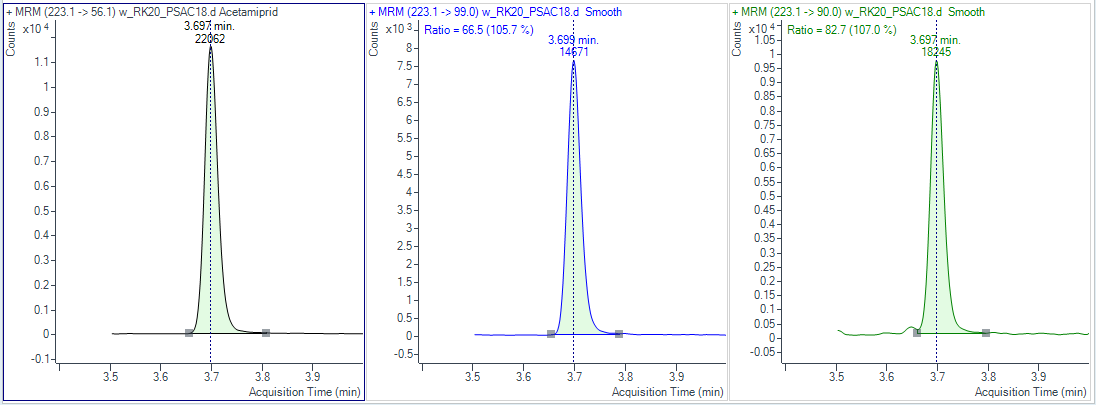


Aclonifen (method M1) 20 µg/kg


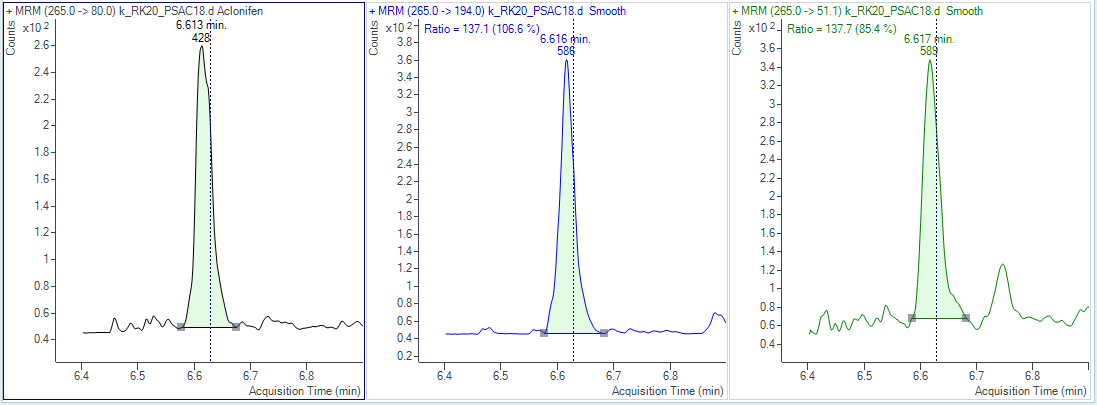


Acrinathrin (method M1) 20 µg/kg


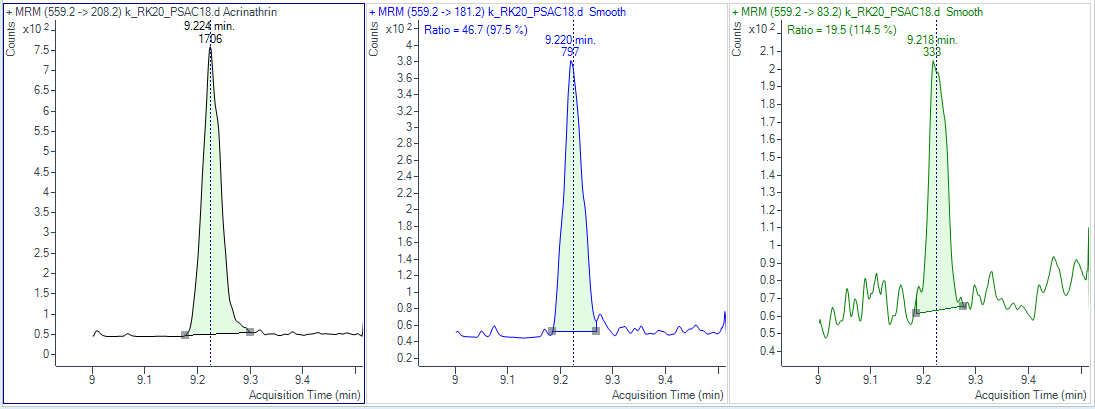


Azoxystrobin (method M3) 20 µg/kg


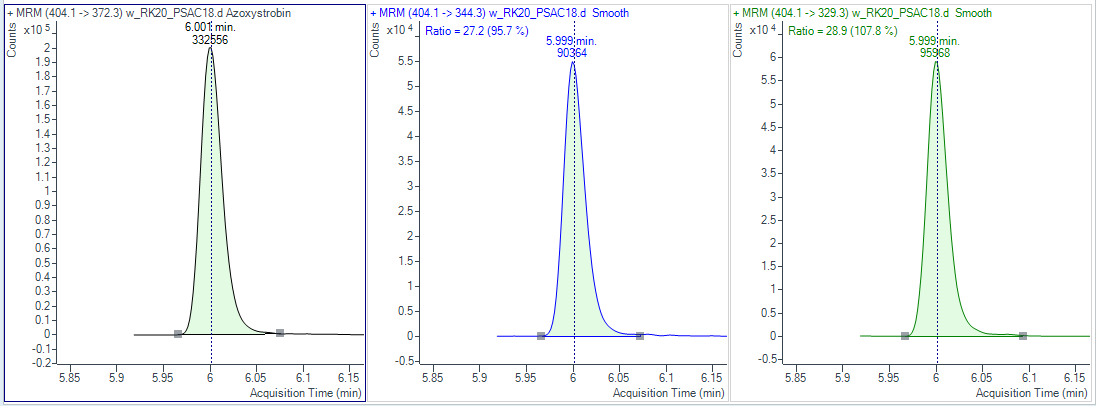


Azoxystrobin-D4 (ISTD)


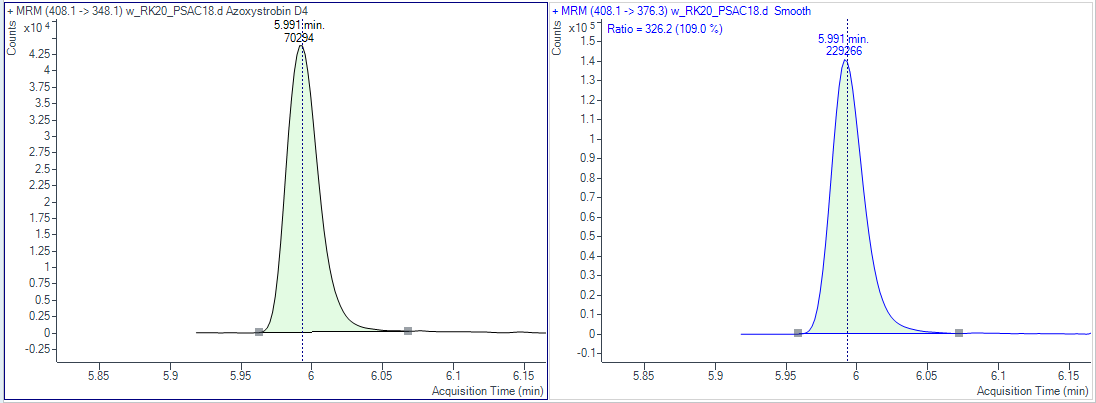


Bendiocarb (method M3) 20 µg/kg


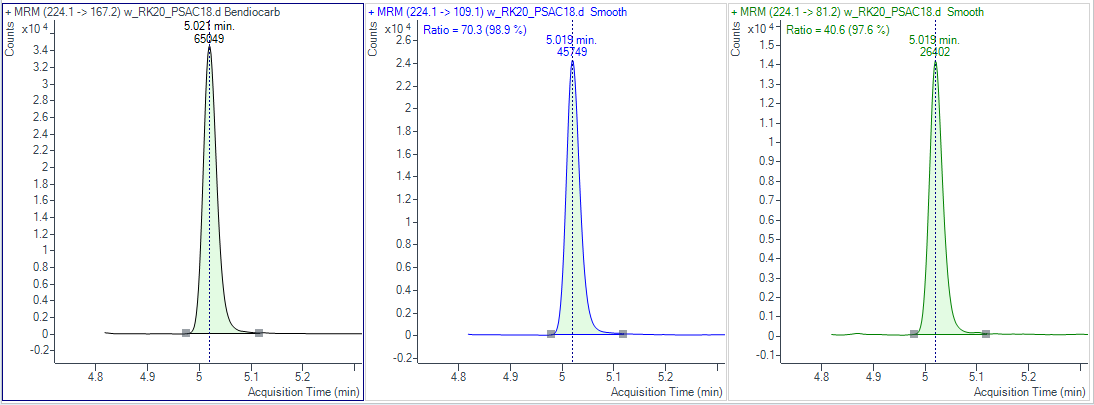


Boscalid (method M3) 20 µg/kg


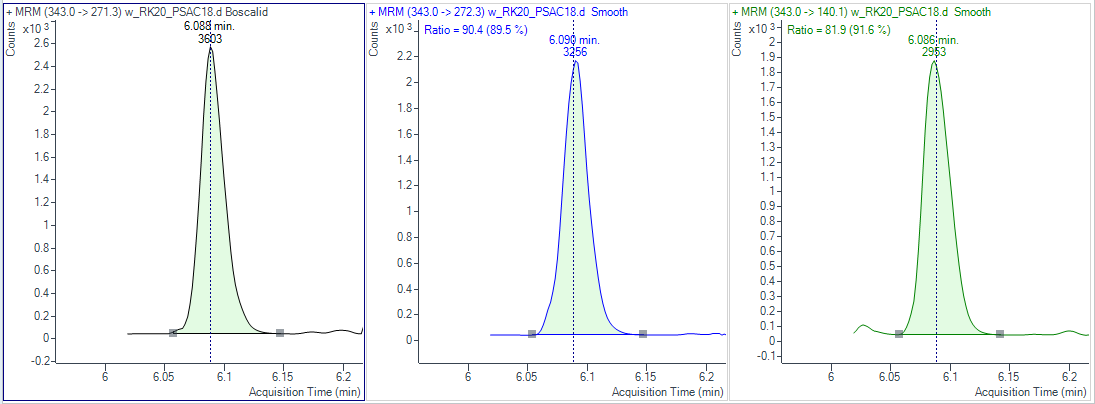


Bromopropylate (method M2) 1000 µg/kg


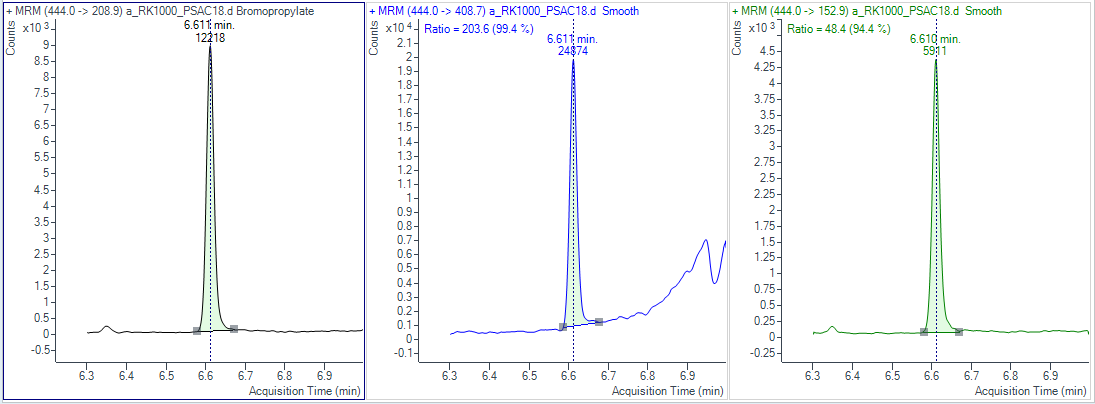


Chlorfenvinphos (method M3) 20 µg/kg


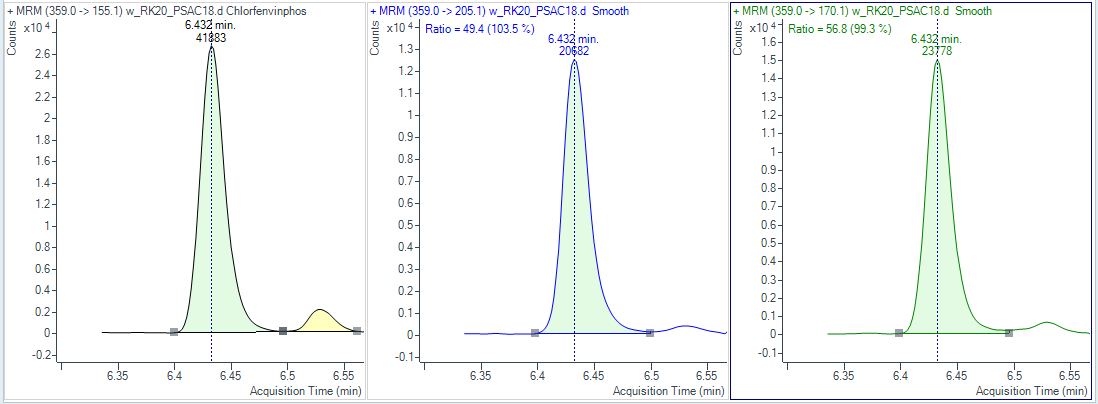


Chlorpyrifos (method M1) 20 µg/kg


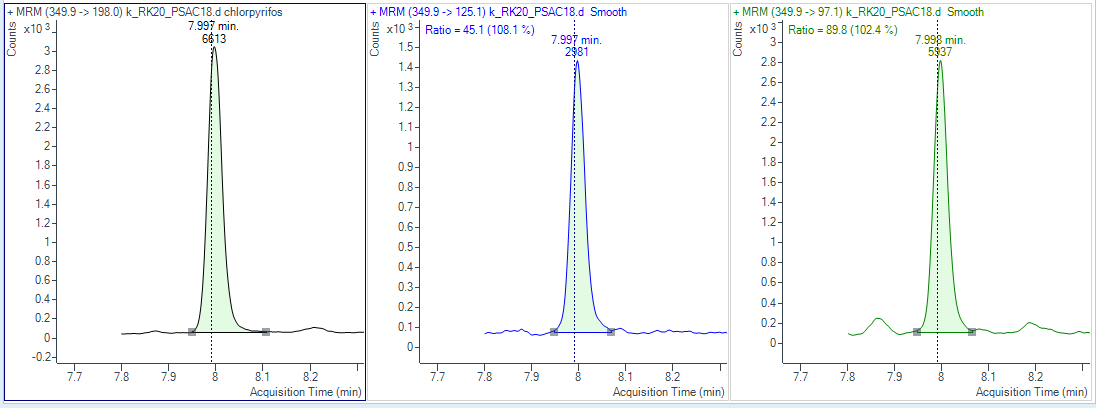


Clothianidin (method M3) 20 µg/kg


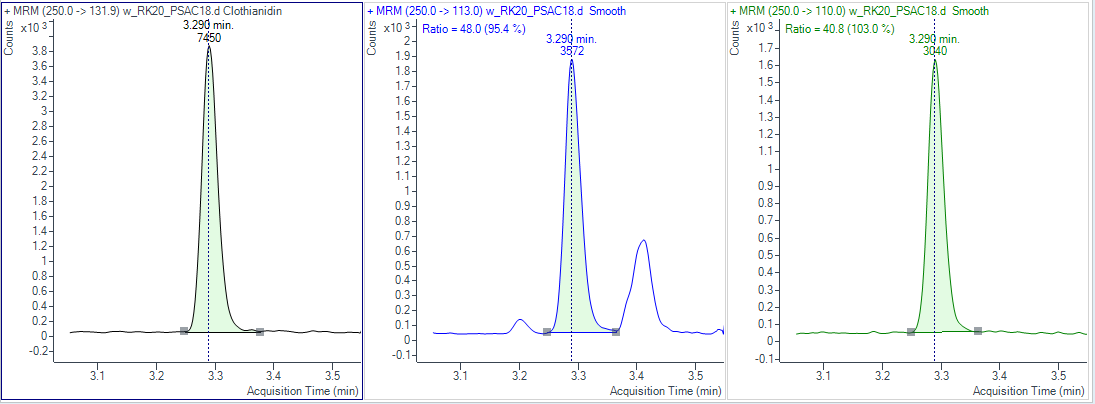


Clothinidin-D3 (ISTD method M1)


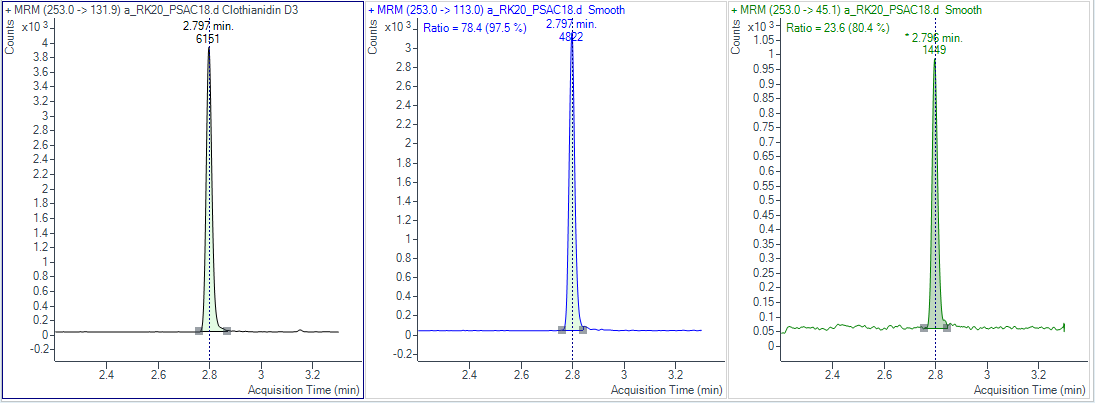


Clothianidin-D3 (ISTD method M2)


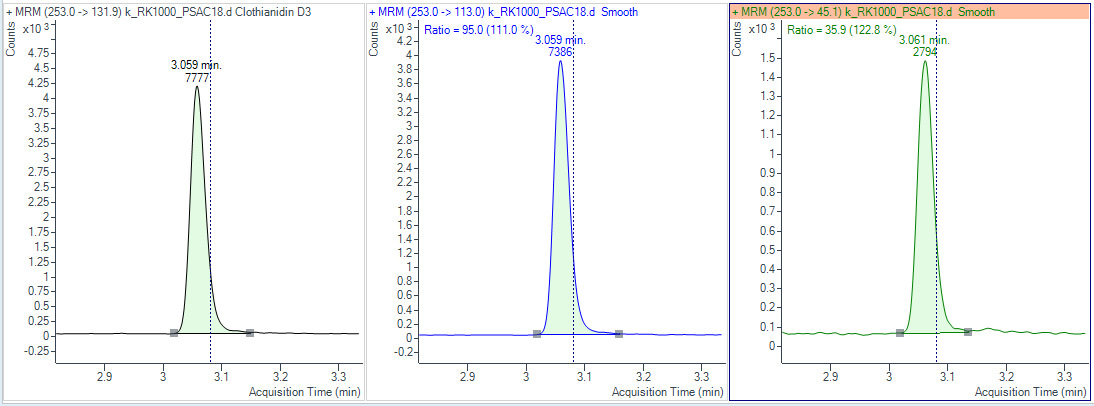


Clothianidin-D3 (method M3)


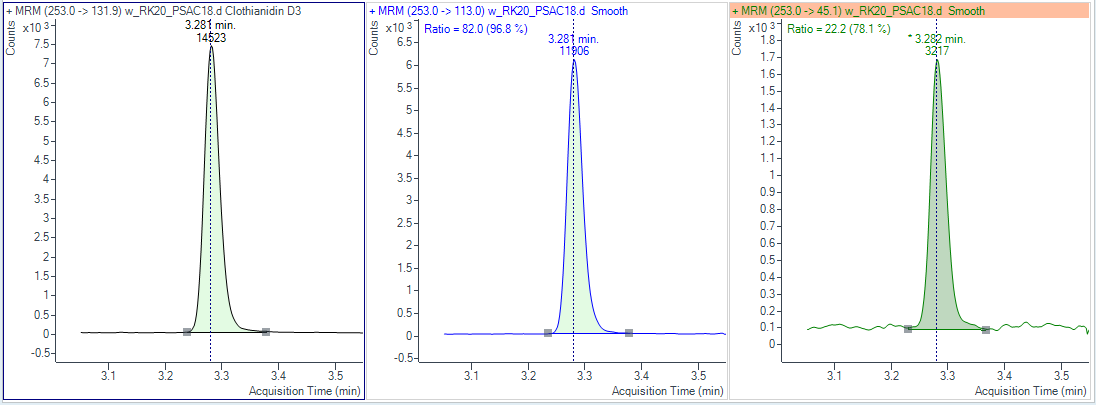


Coumaphos (method M2) 20 µg/kg


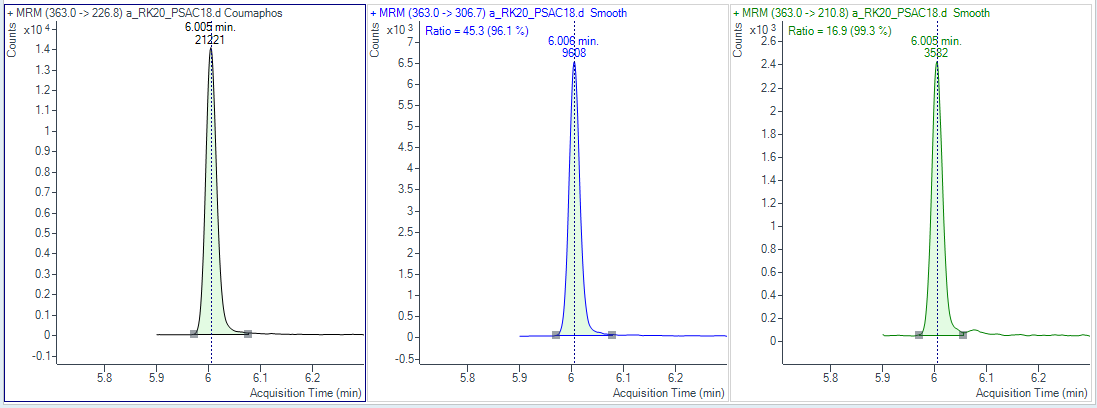


Lambda-cyhalothrin (method M1) 1000 µg/kg


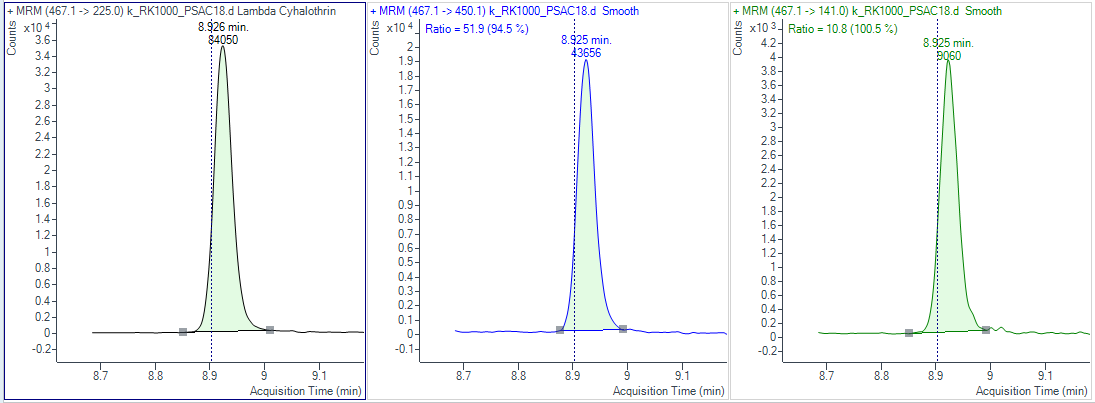


Zeta-cypermethrin (method M1) 1000 µg/kg


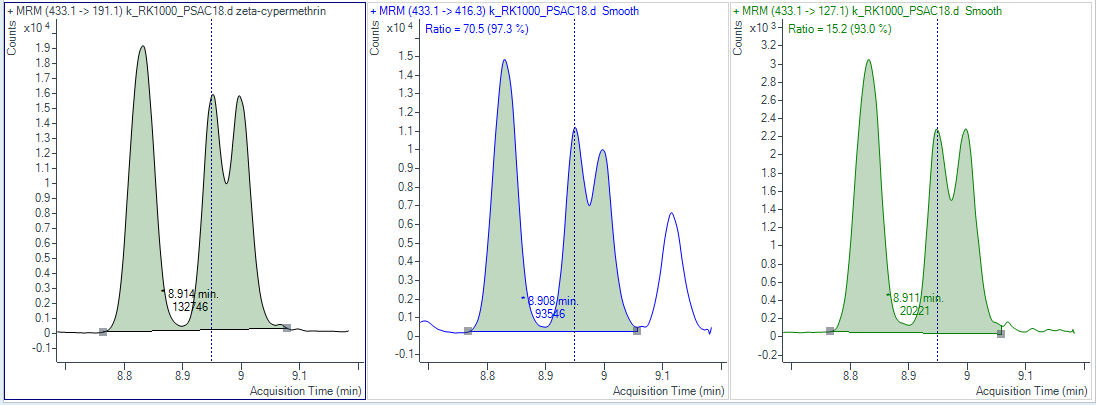


Cyproconazole (method M3) 20 µg/kg


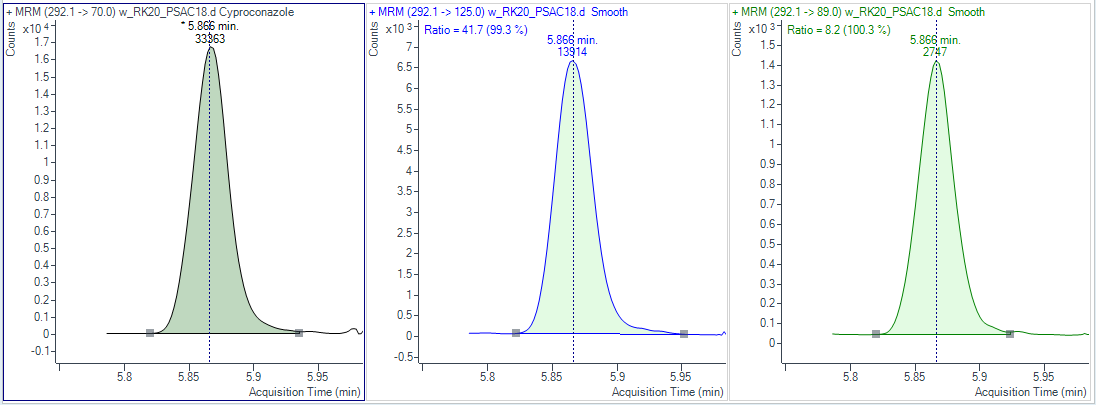


Cyproconazole-D3 (ISTD)


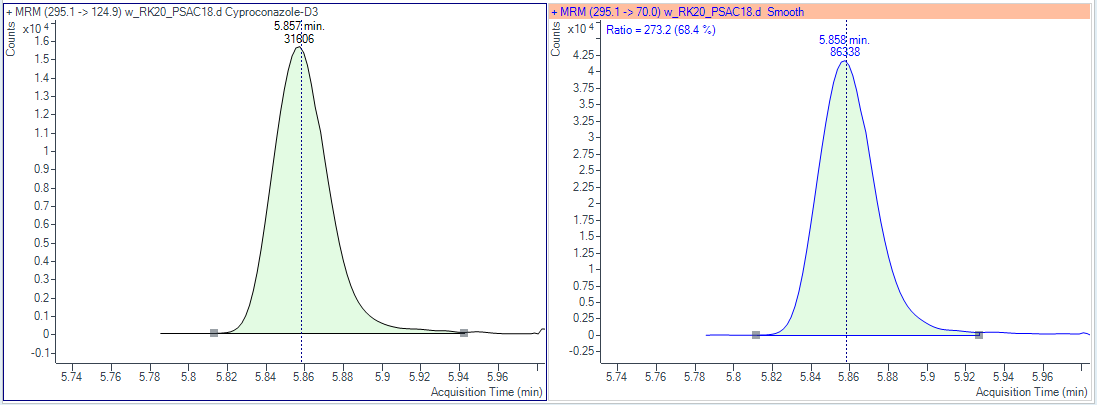


Cyprodinil (method M3) 20 µg/kg


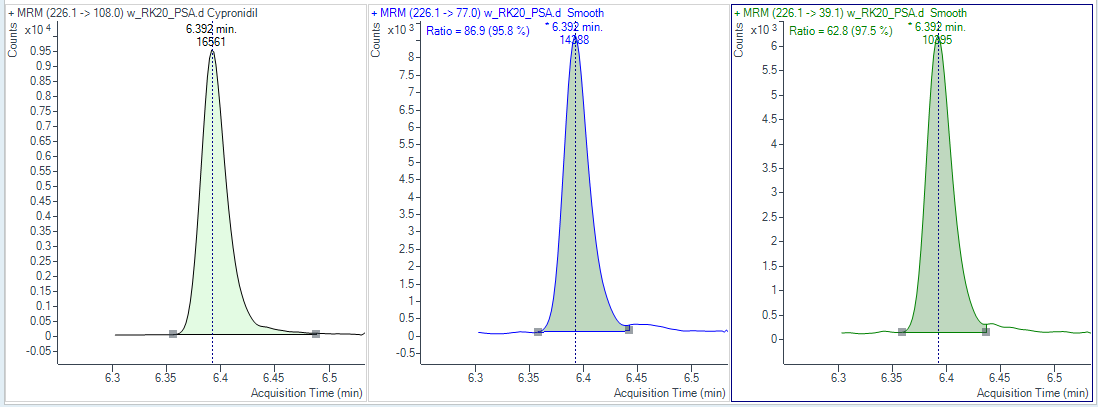


Deltamethrin (method M1) 1000 µg/kg


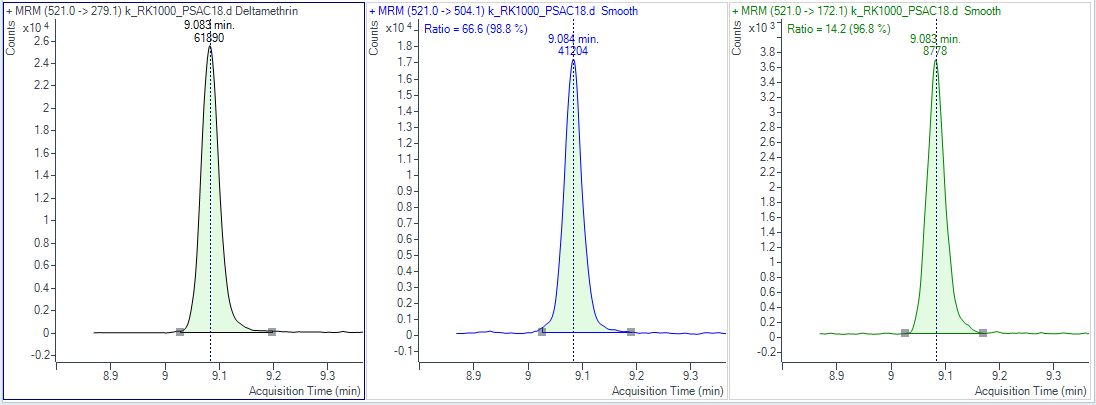


Difenoconazole (method M3) 20 µg/kg


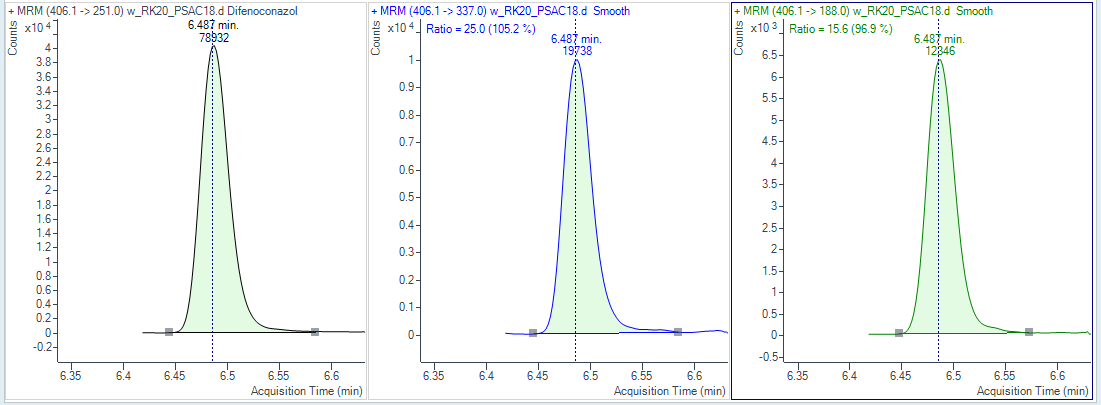


Dimethoate (method M3) 20 µg/kg


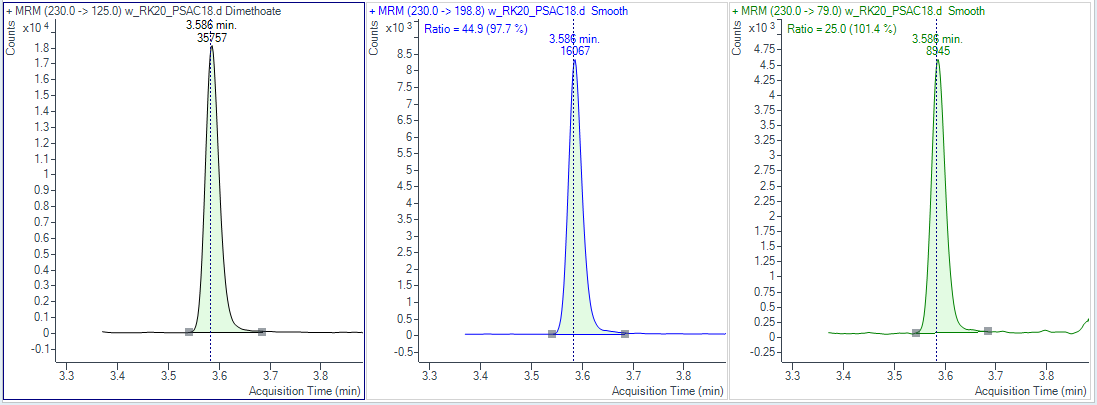


Dimoxystrobin (method M3) 20 µg/kg


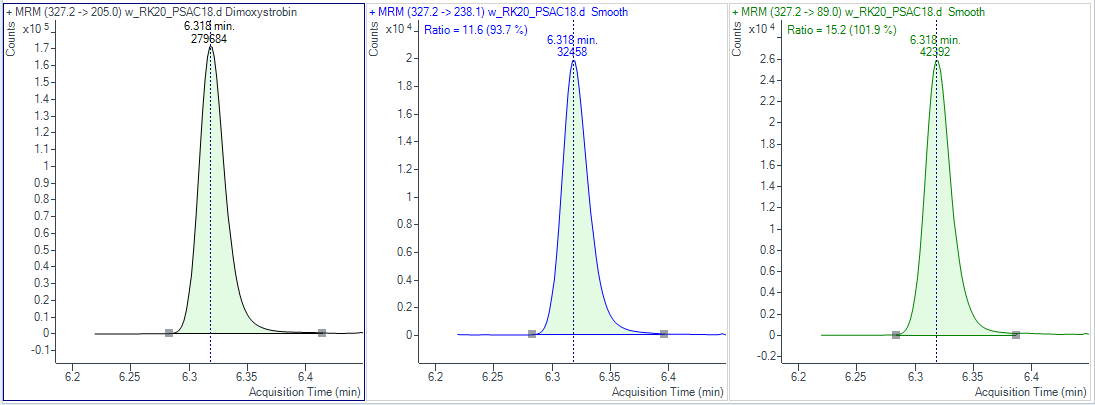


DMF (Amitraz metabolite; method M3) 20 µg/kg


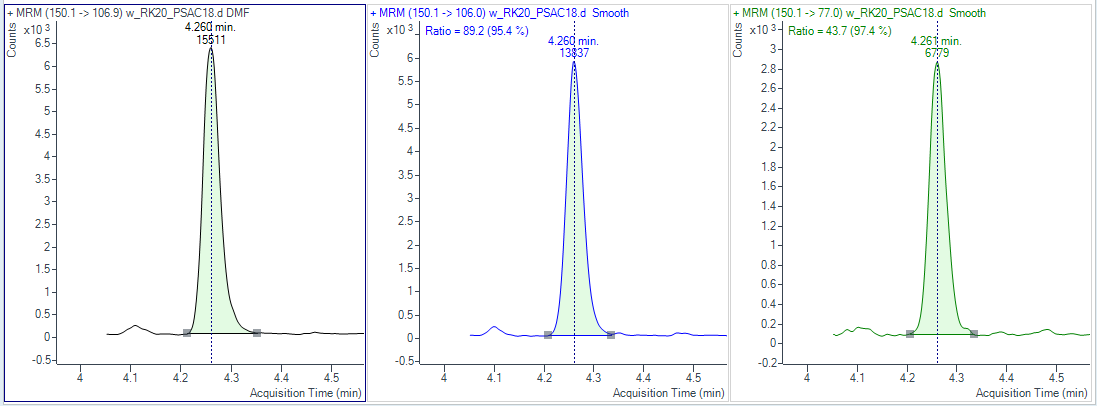


Fenhexamid (method M3) 1000 µg/kg


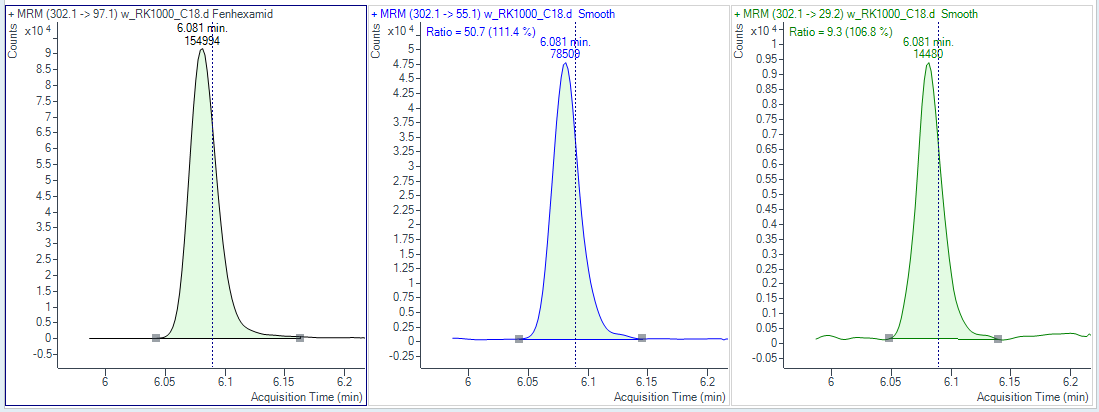


Fenitrothion (method M1) 20 µg/kg


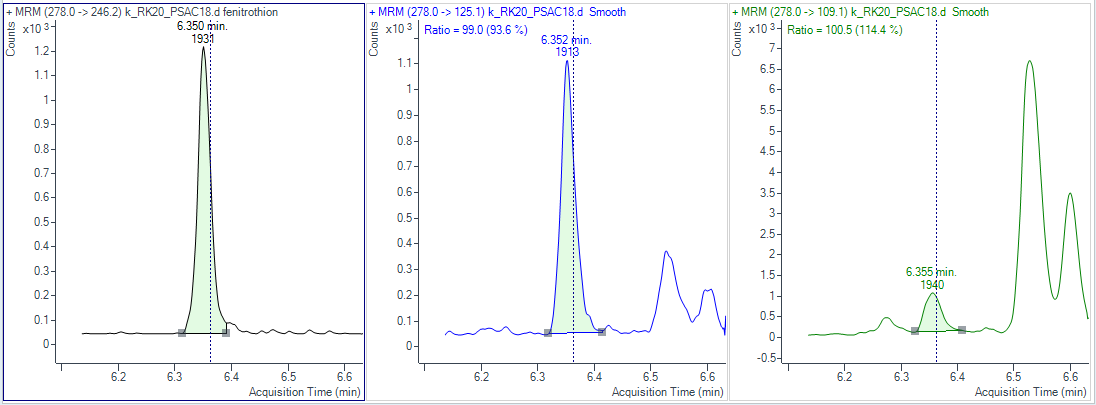


(E)-Fenpyroximate (method M3) 20 µg/kg


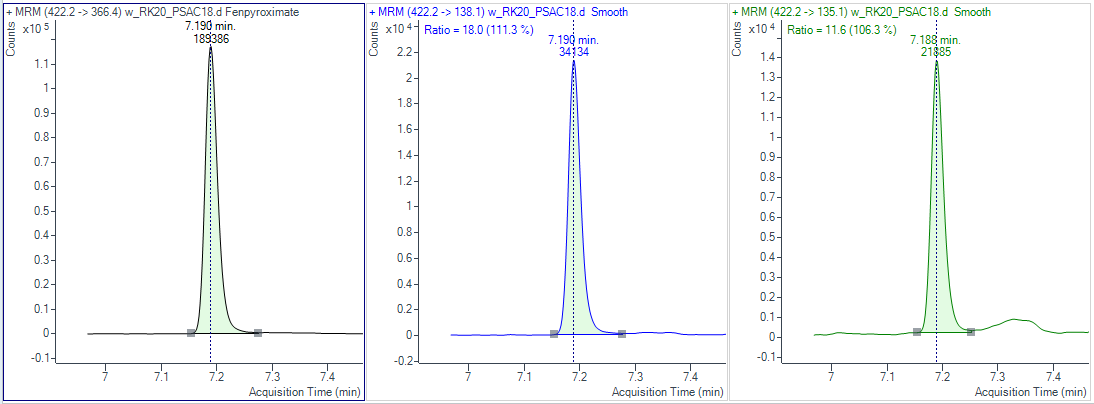


Fipronil (method M2) 20 µg/kg


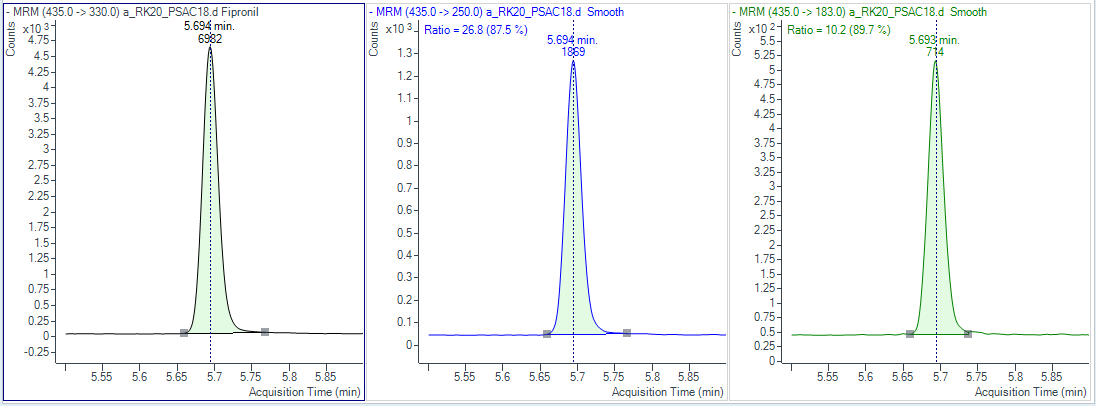


Fludioxonil (method M1) 20 µg/kg


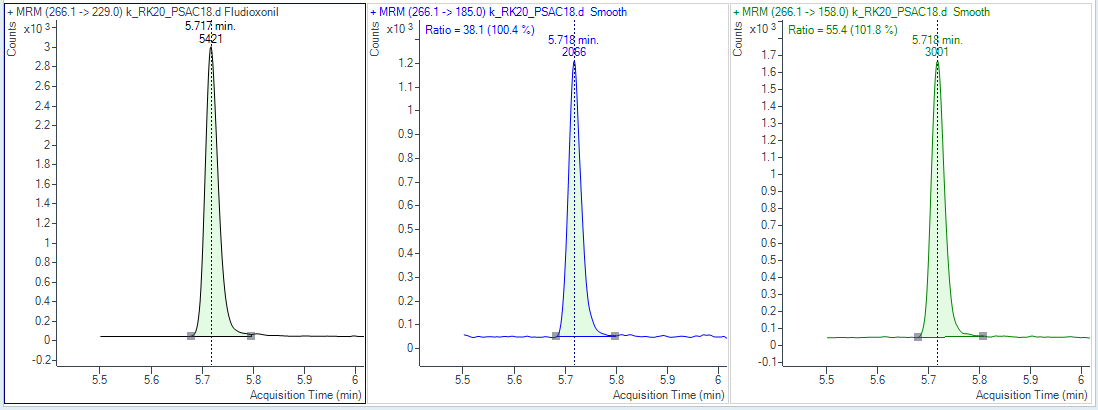


Flufenacet (method M1) 20 µg/kg


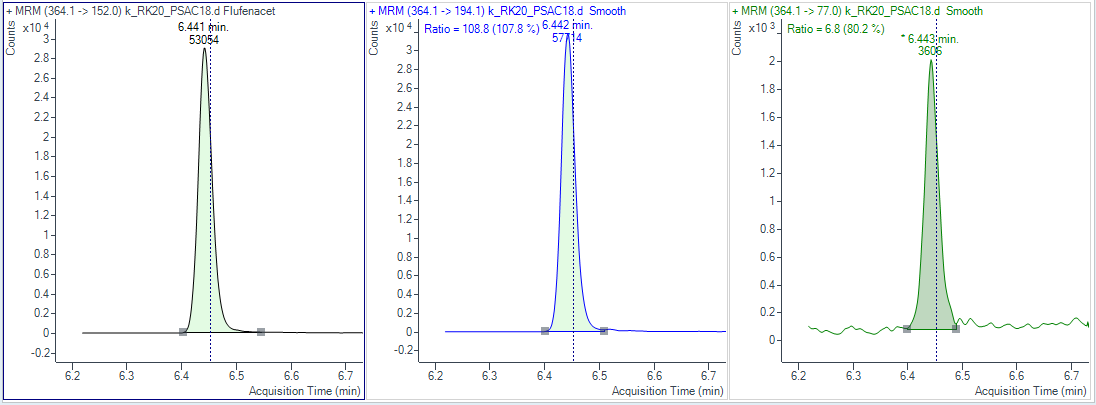


Fluopyram (method M3) 20 µg/kg


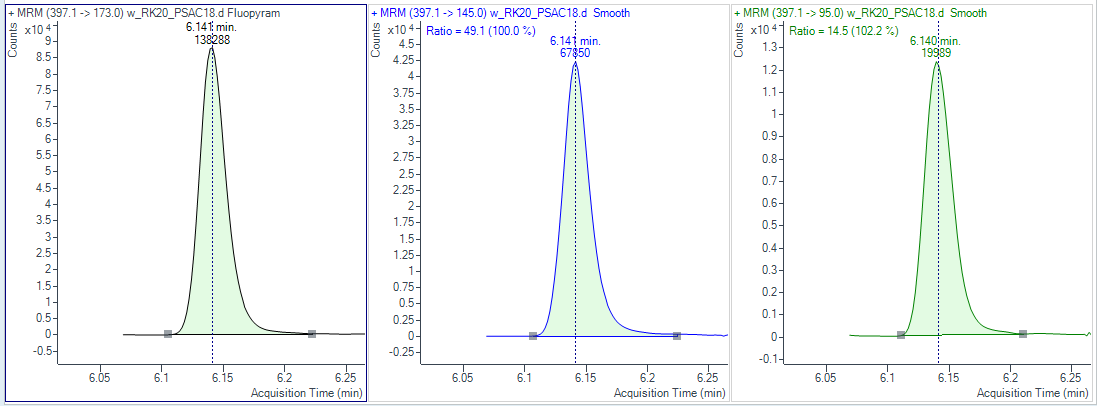


Fluopyram-D4 (ISTD)


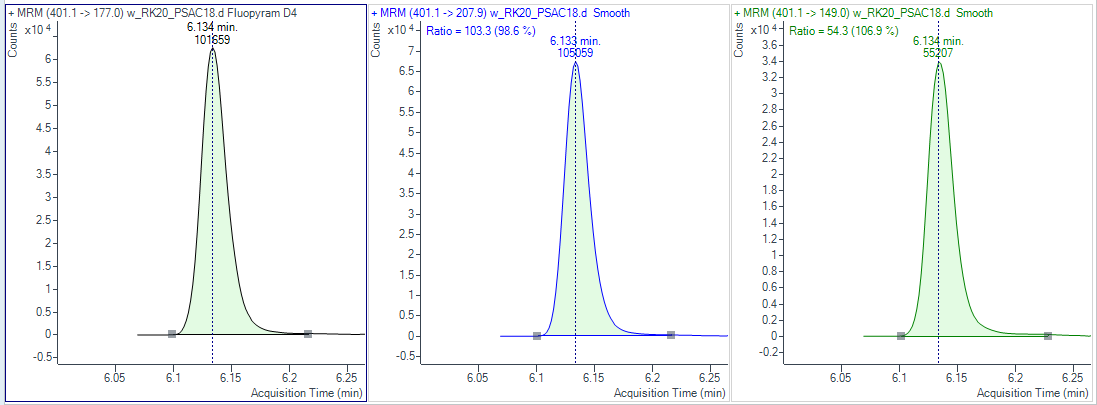


Flupyradifurone (method M3) 20 µg/kg


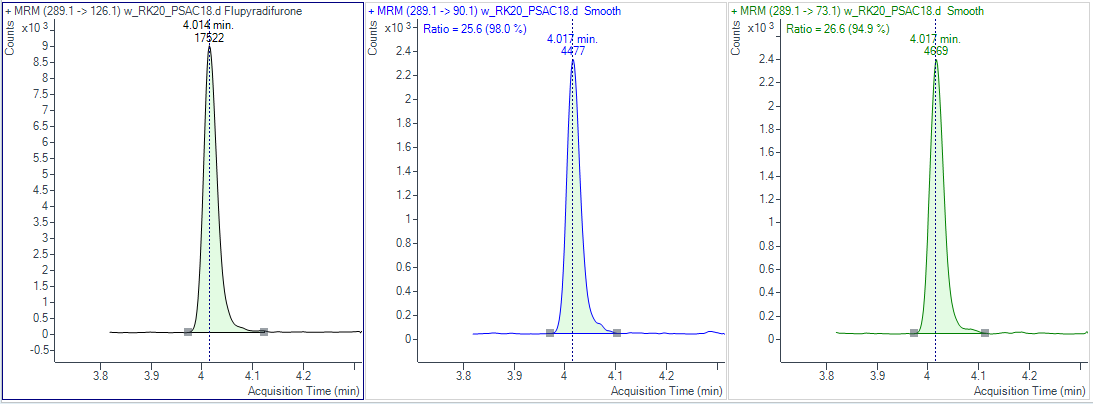


Tau-fluvalinate (method M2) 20 µg/kg


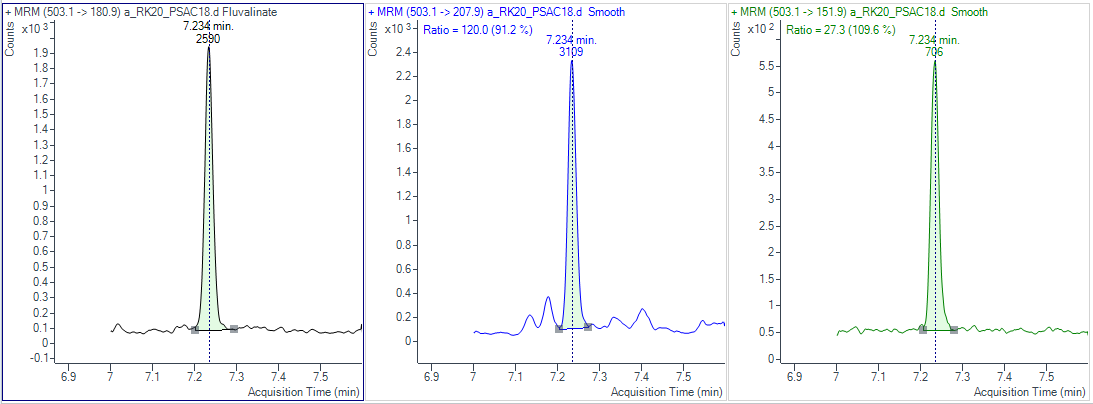


Hexythiazox (method M3) 20 µg/kg


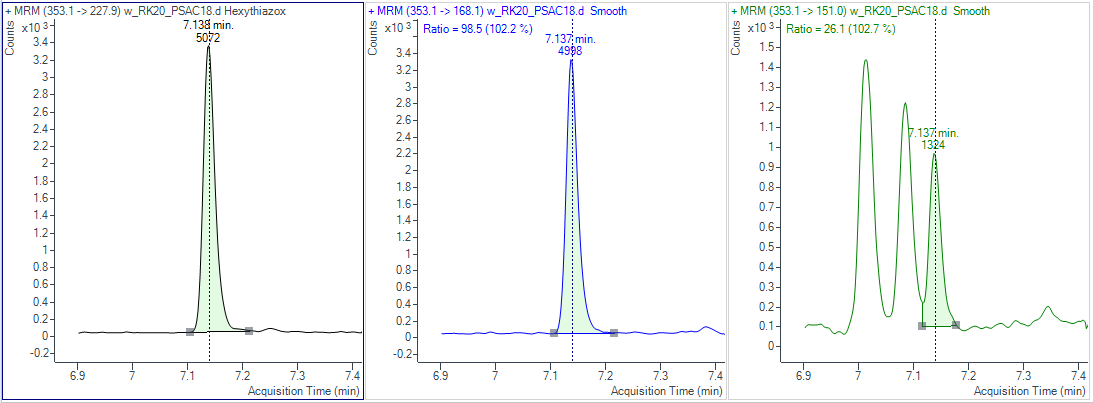


Imidacloprid (method M3) 20 µg/kg


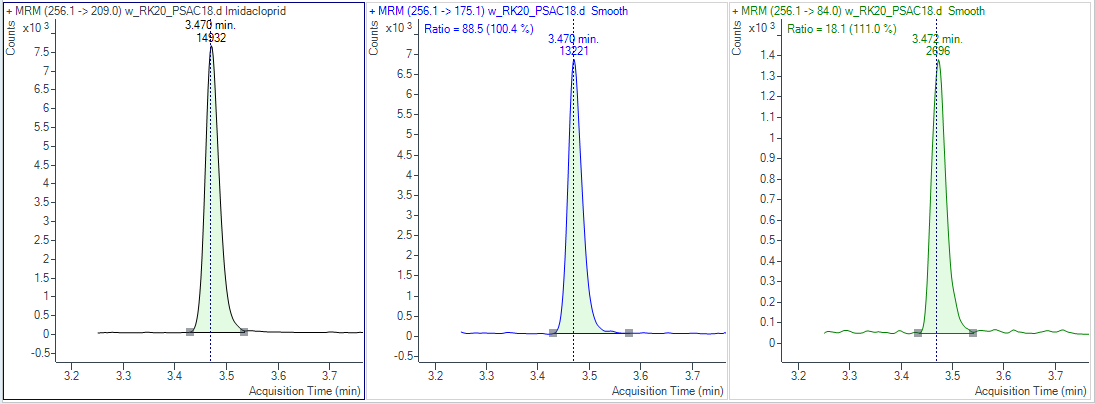


Indoxacarb (method M3) 20 µg/kg


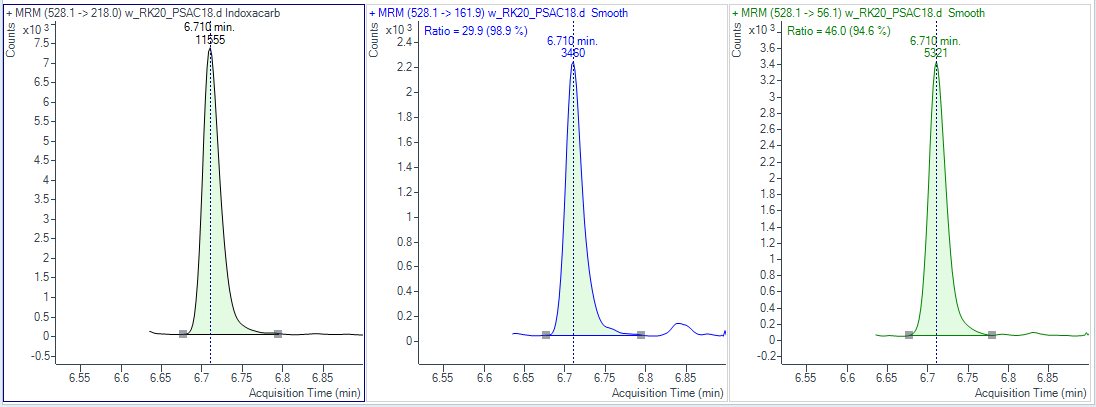


Iprovalicarb (method M3) 20 µg/kg


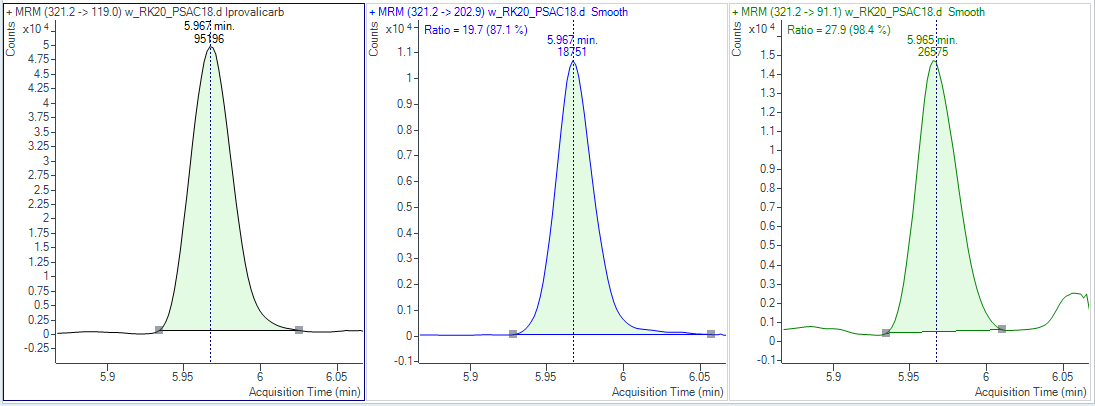


Mandipropamid (method M3) 20 µg/kg


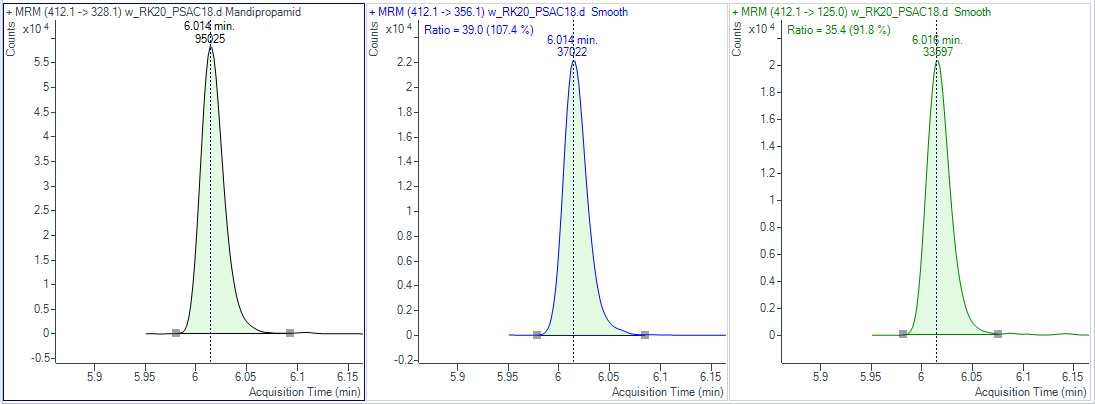


Mepanipyrim (method M3) 20 µg/kg


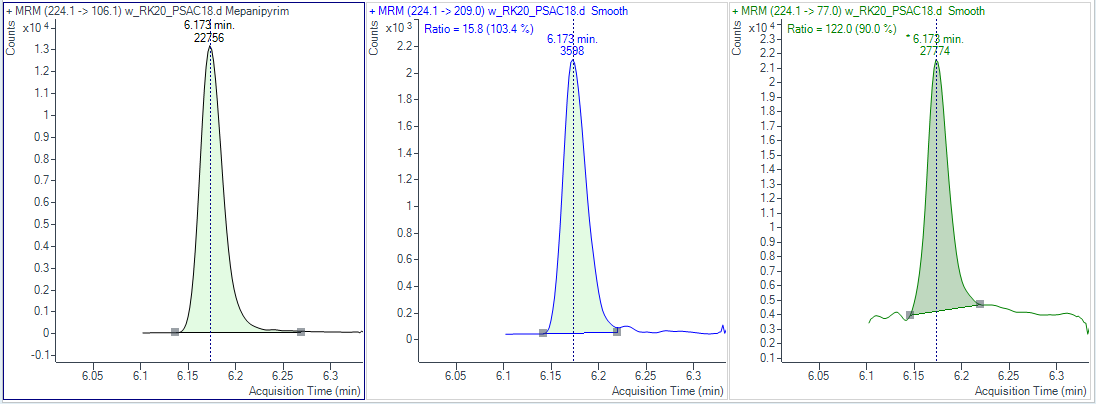


Metconazole (method M3) 20 µg/kg


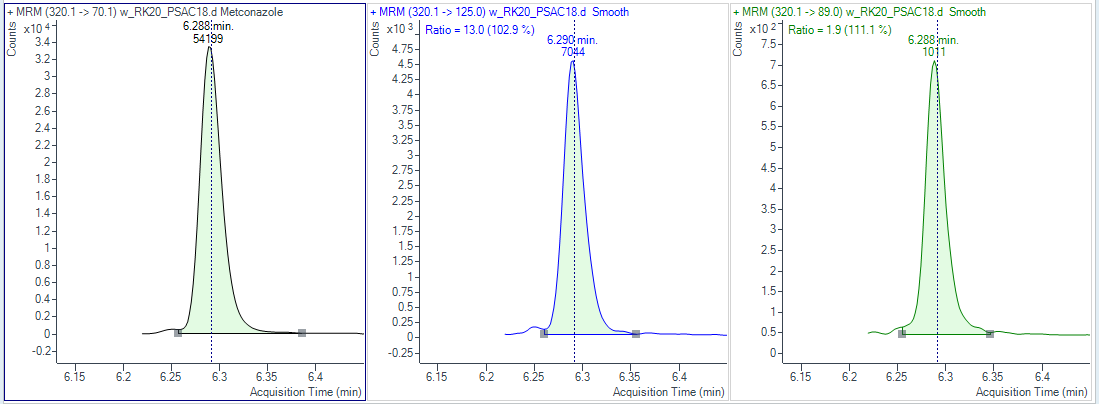


Methoxyfenozide (method M1) 20 µg/kg


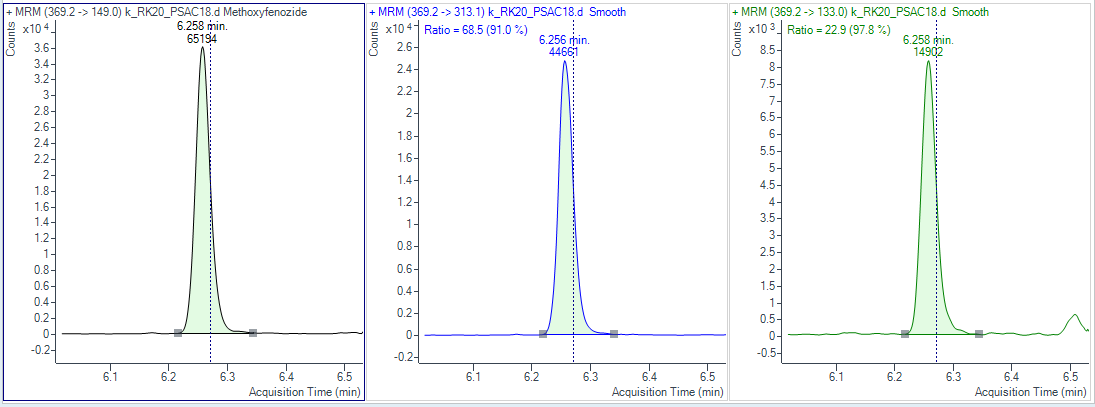


Permethrin (method M1) 20 µg/kg


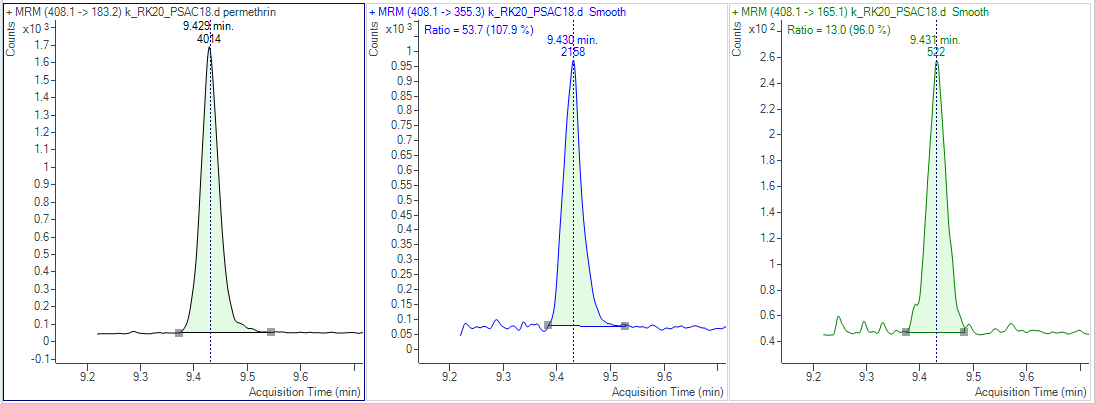


Piperonyl butoxide (method M3) 20 µg/kg


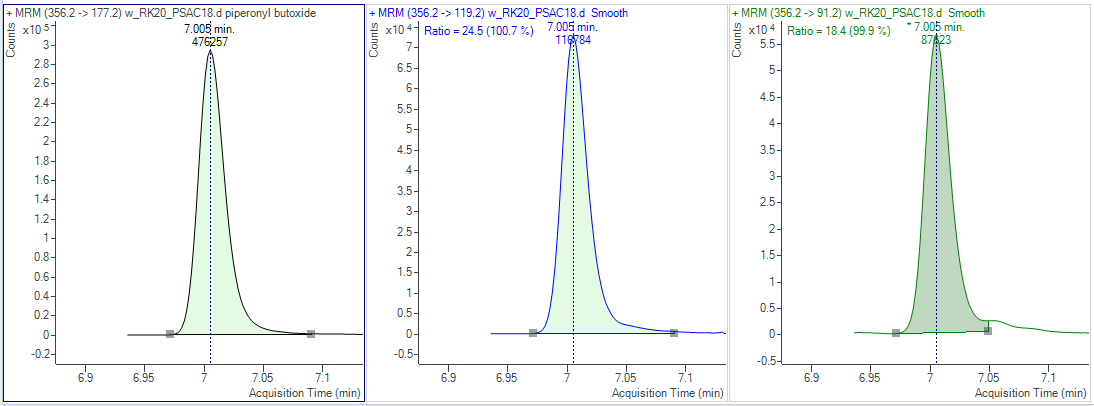


Propoxur (method M3) 20 µg/kg


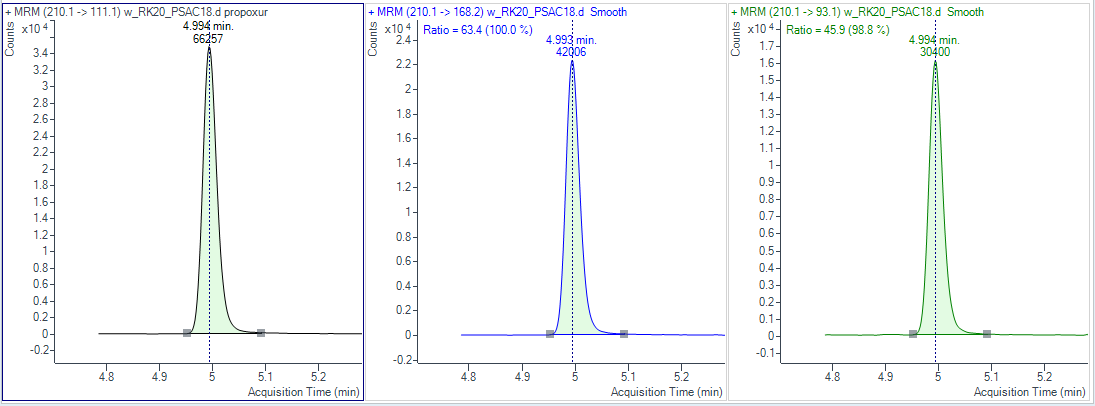


Prosulfocarb (method M3) 20 µg/kg


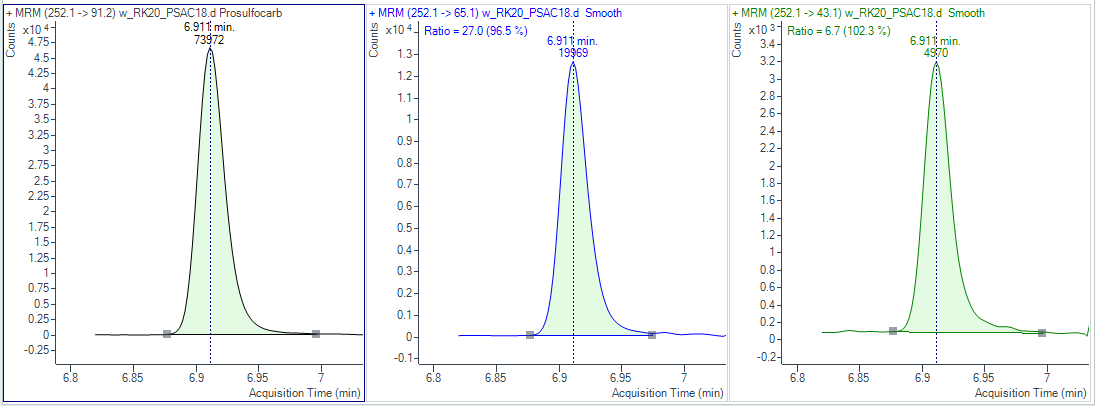


Desthio-prothioconazole (method M3) 20 µg/kg


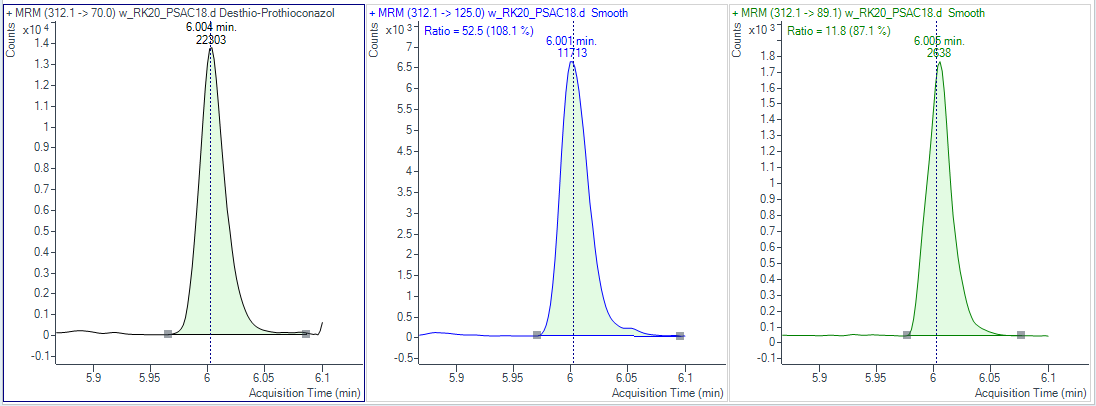


Pyraclostrobin (method M3) 20 µg/kg


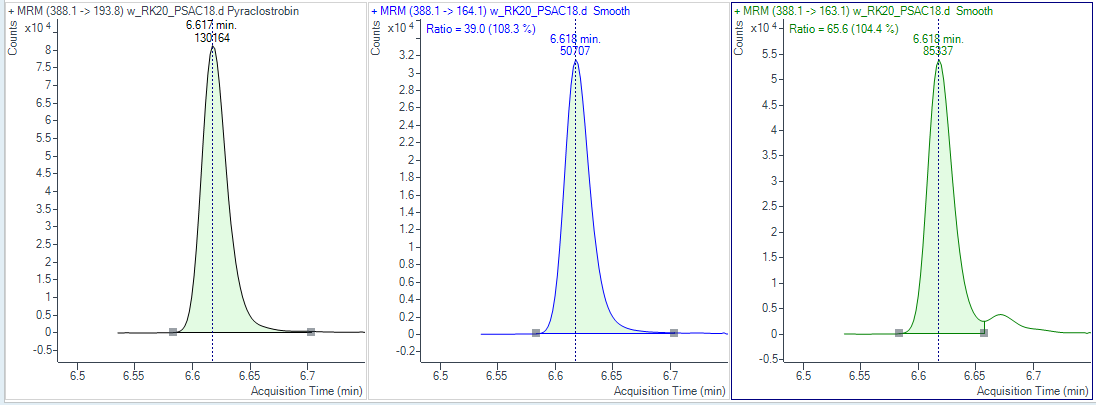


Spinosad (Spinosyn A; method M3) 16.8 µg/kg


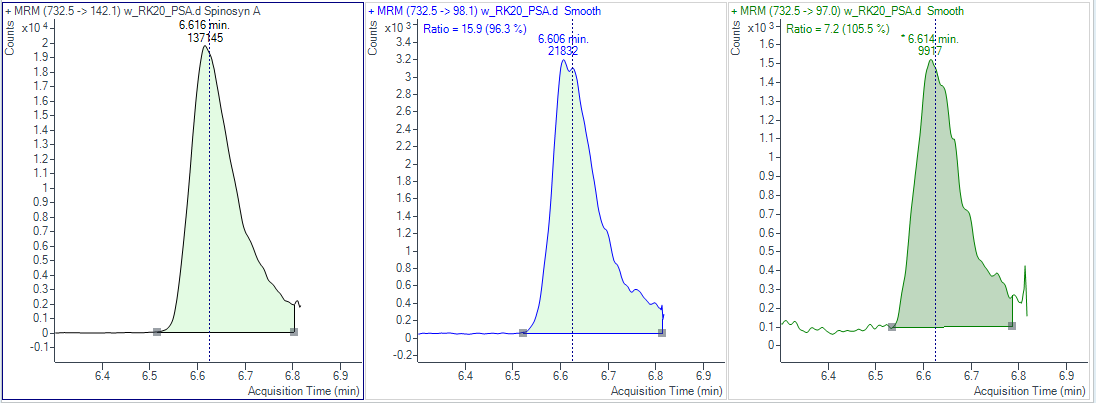


Spinosad (Spinosyn D; method M3) 160 µg/kg


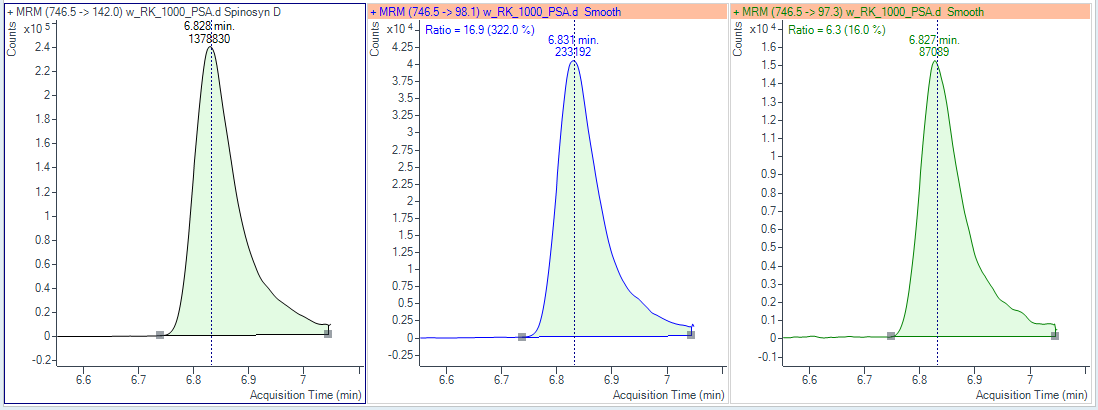


Spirodiclofen (method M3) 20 µg/kg


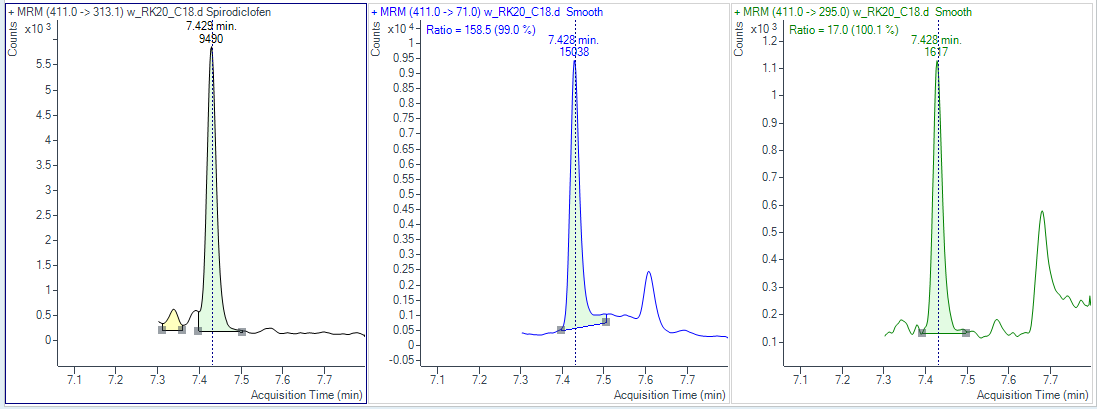


Tebuconazole (method M1) 20 µg/kg


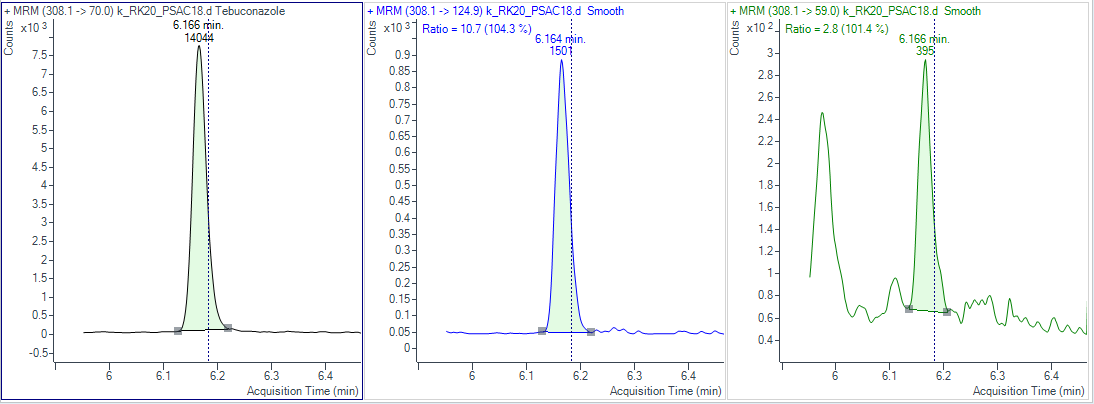


Terbuthylazine (method M3) 20 µg/kg


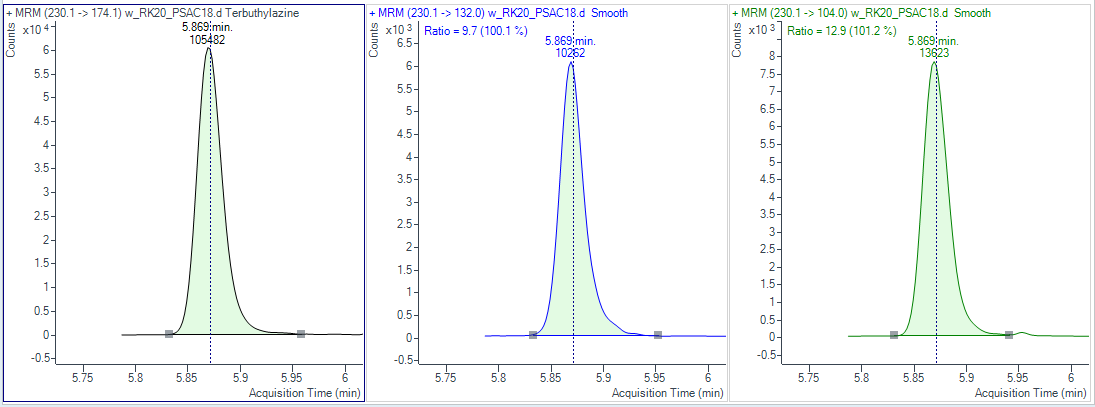


Terbuthylazine-D5 (ISTD)


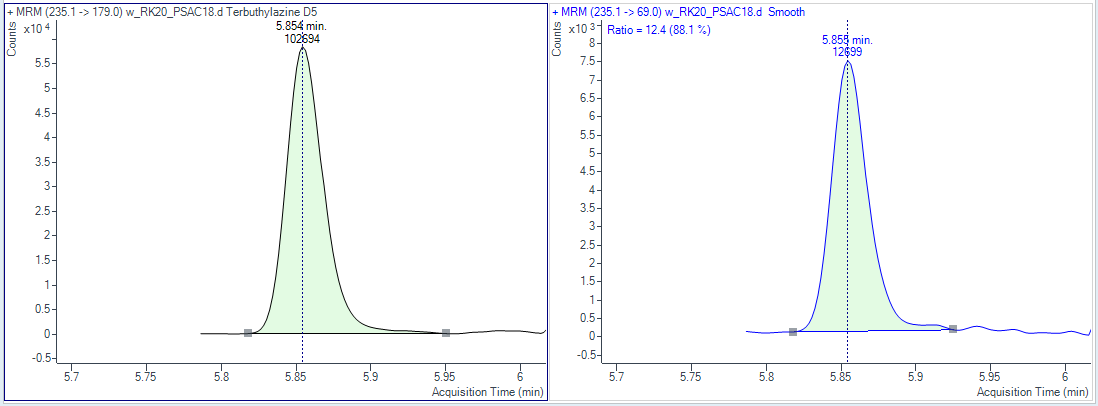


Thiacloprid (method M3) 20 µg/kg


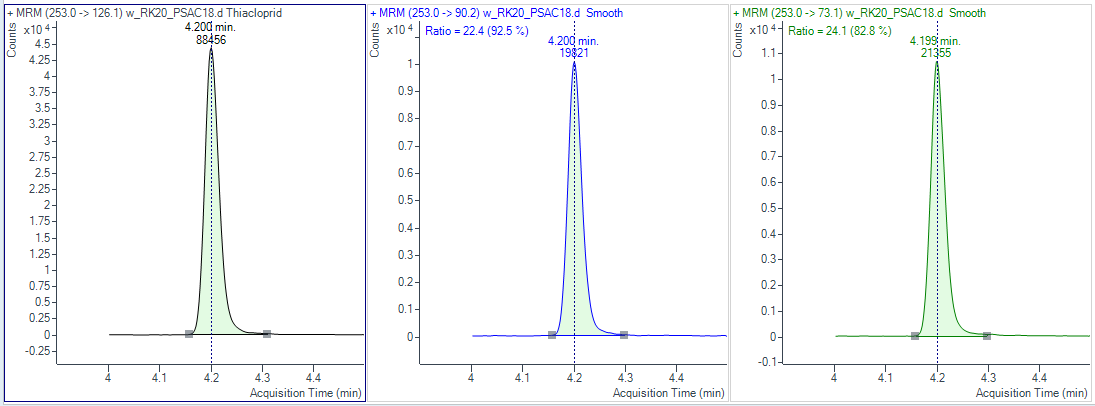


Thiacloprid-D4 (ISTD)


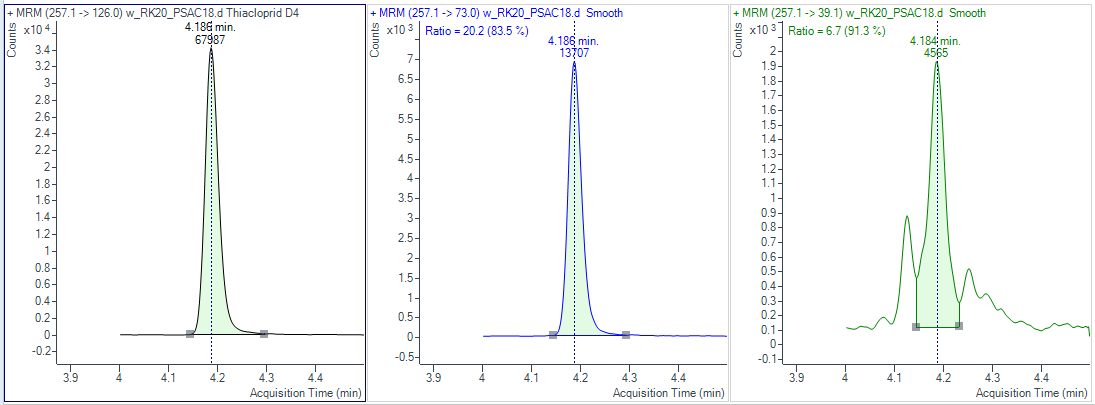


Thiamethoxam (method M3) 20 µg/kg


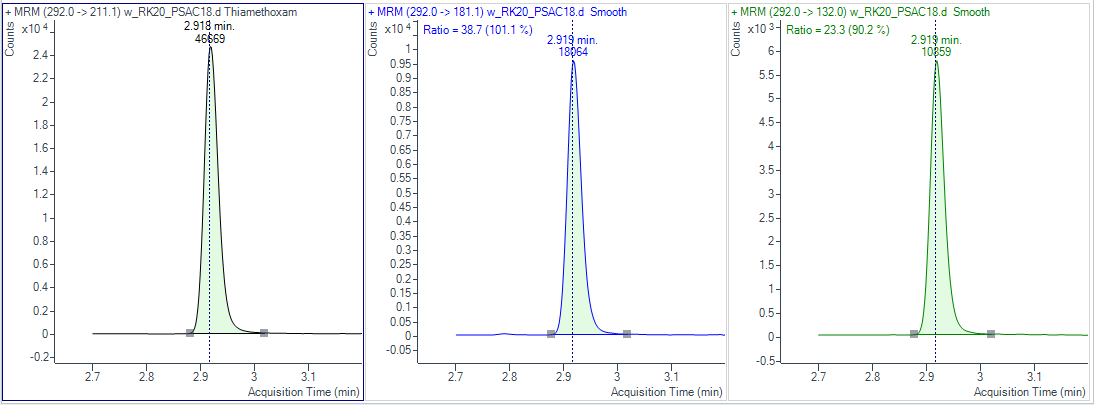


Trifloxystrobin (method M3) 20 µg/kg


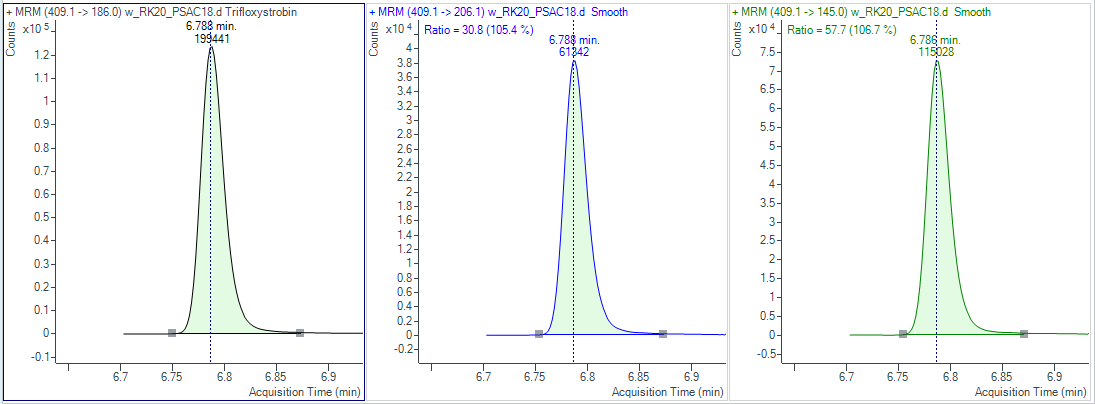

Supplement: Supplementary file 1 — Supplementary file1 (DOCX 2.44 MB) [file 11356_2024_35224_MOESM1_ESM.docx]
